# Prenatal Exposure to Triclocarban Impairs ESR1 Signaling and Disrupts Epigenetic Status in Sex-Specific Ways as Well as Dysregulates the Expression of Neurogenesis- and Neurotransmitter-Related Genes in the Postnatal Mouse Brain

**DOI:** 10.3390/ijms222313121

**Published:** 2021-12-04

**Authors:** Agnieszka Wnuk, Joanna Rzemieniec, Karolina Przepiórska, Bernadeta Angelika Pietrzak, Marzena Maćkowiak, Małgorzata Kajta

**Affiliations:** 1 Laboratory of Neuropharmacology and Epigenetics, Department of Pharmacology, Maj Institute of Pharmacology, Polish Academy of Sciences, Smetna Street 12, 31-343 Krakow, Poland; rzemien@if-pan.krakow.pl (J.R.); przepior@if-pan.krakow.pl (K.P.); pietrzak@if-pan.krakow.pl (B.A.P.); 2 Laboratory of Pharmacology and Brain Biostructure, Department of Pharmacology, Maj Institute of Pharmacology, Polish Academy of Sciences, Smetna Street 12, 31-343 Krakow, Poland; mackow@if-pan.krakow.pl

**Keywords:** DNA methylation, environmentally pervasive chemicals, estrogen receptors, microarrays, xenobiotic receptors

## Abstract

Triclocarban is a highly effective and broadly used antimicrobial agent. Humans are continually exposed to triclocarban, but the safety of prenatal exposure to triclocarban in the context of neurodevelopment remains unknown. In this study, we demonstrated for the first time that mice that had been prenatally exposed to environmentally relevant doses of triclocarban had impaired estrogen receptor 1 (ESR1) signaling in the brain. These mice displayed decreased mRNA and protein expression levels of ESR1 as well as hypermethylation of the *Esr1* gene in the cerebral cortex. Prenatal exposure to triclocarban also diminished the mRNA expression of *Esr2*, *Gper1*, *Ahr*, *Arnt*, *Cyp19a1*, *Cyp1a1*, and *Atg7*, and the protein levels of CAR, ARNT, and MAP1LC3AB in female brains and decreased the protein levels of BCL2, ARNT, and MAP1LC3AB in male brains. In addition, exposure to triclocarban caused sex-specific alterations in the methylation levels of global DNA and estrogen receptor genes. Microarray and enrichment analyses showed that, in males, triclocarban dysregulated mainly neurogenesis-related genes, whereas, in females, the compound dysregulated mainly neurotransmitter-related genes. In conclusion, our data identified triclocarban as a neurodevelopmental risk factor that particularly targets ESR1, affects apoptosis and autophagy, and in sex-specific ways disrupts the epigenetic status of brain tissue and dysregulates the postnatal expression of neurogenesis- and neurotransmitter-related genes.

## 1. Introduction

Triclocarban (3,4,4′-trichlorocarbanilide, TCC) is a broad-spectrum antimicrobial agent that is added to a wide variety of personal and health care products, medical devices, plastics, and fabrics [1]. Excessive use of compounds with antibacterial potential has resulted in antimicrobial resistance, which contributes to 700,000 deaths annually [2]. Currently, it is known that fetuses are susceptible to the potential effects of triclocarban since this compound possesses high placental transferability and lower excretion capacity from fetal bodies [3]. Research conducted over the past 6 years shows that triclocarban has a strong influence on embryonic and fetal development. Kennedy et al. [4] provided evidence that exposure to triclocarban during lactation influences the development of neonates and affects neonatal rat survival. Another in vivo study based on maternal exposure of mouse offspring through breastfeeding showed that triclocarban accumulates mainly in the brain, heart, and muscle tissue of the exposed offspring. The potential toxicity of triclocarban was recently supported by the demonstration of a strong correlation between high concentrations of triclocarban in urine and increased risk of preterm birth in a cohort of 922 pregnant women in Puerto Rico [5]. Although triclocarban has been detected in the human brain [6,7], data on the molecular mechanism through which triclocarban affects the developing mammalian nervous system are scarce. Our previous studies based on an in vitro model indicated that triclocarban has the ability to induce apoptosis, impair autophagy, and disrupt epigenetic status in brain neurons. These effects were accompanied by the disruption of estrogen receptors and AHR/CAR signaling pathways [8,9]. Therefore, based on our in vitro studies, we postulated that triclocarban should be categorized as a neurodevelopmental risk factor whose action may provide a fetal basis for the adult onset of neurological diseases.

Disturbed homeostasis/balance among apoptosis, autophagy, and the expression of genes related to neurodevelopment has already been recognized to underline several disorders, including attention-deficit/hyperactivity disorder (ADHD), autism spectrum disorder, and intellectual disabilities [10,11,12,13]. The correlation among these processes is also strongly modulated by steroid and xenobiotic receptors, which act mainly as transcription factors. Estrogen receptor (ER) signaling pathways have been found to play crucial roles during neurodevelopment and in the pathophysiology of psychiatric disorders [14]. ERs, i.e., ESR1, ESR2, and GPER1 (previously known as ERα, ERβ, GPR30), are widely expressed across the brain, and different ERs are dominant in individual brain regions, where they exert effects on key neurotransmitter systems and on control of the cell death/survival balance. In addition to ERs, aryl hydrocarbon receptor (AHR) and constitutive androstane receptor (CAR) signaling pathways are also pivotal for neurodevelopment since they contribute to cell proliferation, migration, and differentiation as well as to proper blood–brain barrier (BBB) formation [15,16]. The main regulatory mechanisms that coordinate brain development, including synaptic pruning and differentiation, by changing the expression of specific genes are epigenetic processes [17]. Epigenetic delay with particular emphasis on aberrant DNA methylation has been recognized to lead to neurodevelopmental disorders, and genome-wide DNA methylation analyses have demonstrated the usefulness of epigenome assessment in the clinical screening of patients with neurodevelopmental disorders [18].

Although triclocarban is more frequently identified as a neurodevelopmental risk factor, it is unknown what are the molecular (including epigenetic) mechanisms through which triclocarban affects the developing mammalian nervous system and evokes long-term effects that are revealed postnatally needs to be clarified. In this study, we aimed to explore the mechanisms through which prenatal exposure to triclocarban affects postnatal mice of both sexes, with a particular emphasis on apoptosis-, autophagy-, steroid/xenobiotic-receptor-related expression, and epigenetic modifications that occur in response to triclocarban. To evaluate the effects of triclocarban exposure on neurodevelopment, the expression measurements of neurogenesis- and neurotransmitters-related genes were followed by enrichment analyses.

## 2. Results

### 2.1. Triclocarban Downregulated Receptor Signaling in One-Month-Old Mice That Were Prenatally Exposed to Triclocarban

#### 2.1.1. Triclocarban Inhibited Esr1 mRNA Expression and Decreased the Protein Levels of ESR1 and ARNT in One-Month-Old Male Mice That Were Prenatally Exposed to the Chemical

**Figure 1:**
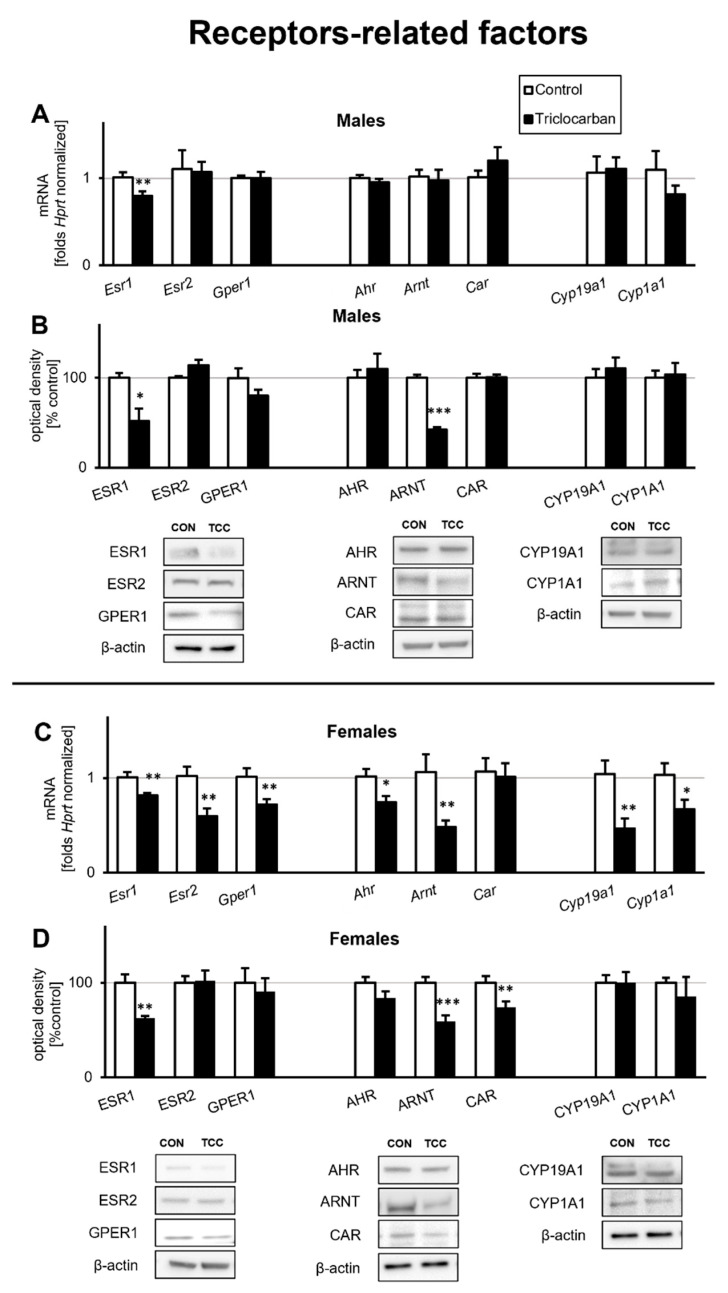
Triclocarban inhibited the mRNA (**A**) and protein (**B**) expression of *Esr1*/ESR1 and the protein expression of ARNT in one-month-old male mice that were prenatally exposed to triclocarban. Triclocarban also inhibited the mRNA (**C**) and protein (**D**) expression of ESR1 and inhibited the mRNA/protein expression of *Ahr*, *Arnt*, ARNT, CAR, *Cyp19a1*, and *Cyp1a1* in one-month-old female mice prenatally exposed to triclocarban. Each bar represents the mean ± SEM of a group of samples. Each control or experimental group consisted of animals from eight different litters, and the samples were collected from six animals per group. All results are expressed as mean ± SEM. * *p* < 0.05, ** *p* < 0.01, and *** *p* < 0.001 versus the control animals.

In one-month-old male mice, prenatal exposure to triclocarban resulted in a significant decrease in the level of *Esr1* mRNA (Figure 1A). In the prefrontal cortex, *Esr1* mRNA expression was reduced by more than 0.2-fold (21% decrease) compared with that in the controls. The expression of other studied mRNAs, i.e., *Esr2*, *Gper1*, *Ahr*, *Arnt*, *Car*, *Cyp19a1*, and *Cyp1a1*, did not change in response to prenatal exposure to triclocarban.

In one-month-old male mice, prenatal exposure to triclocarban reduced the protein levels of ESR1 and ARNT by approximately 50% to 60% (Figure 1B). However, prenatal exposure to the chemical did not affect the levels of ESR2, GPER1, AHR, or CAR receptors or the levels of cytochromes CYP19A1 and CYP1A1 in the prefrontal cortices of one-month-old animals.

#### 2.1.2. Triclocarban Inhibited the mRNA Expression of Esr1, Esr2, Gper1, Ahr, Arnt, Cyp19a1, and Cyp1a1, and Decreased ESR1, ARNT, and CAR Protein Levels in One-Month-Old Female Mice That Were Prenatally Exposed to the Chemical

The prefrontal cortices of one-month-old female mice that were prenatally exposed to triclocarban expressed decreased levels of *Esr1*, *Esr2*, *Gper1*, *Ahr*, *Arnt*, *Cyp19a1*, and *Cyp1a1* mRNAs, but the level of *Car* mRNA remained unchanged (Figure 1C). The mRNA levels of estrogen receptors ranged from 0.6- to 0.8-fold (57% to 81%) that of the controls. The expression levels of *Ahr* and *Arnt* mRNAs were 0.7- and 0.5-fold (73% and 45%) those of the controls, respectively. mRNA expression of cytochromes *Cyp19a1* and *Cyp1a1* was reduced by approximately 0.4–0.6-fold (45% to 65%) compared to the controls.

Immunoblotting confirmed that the prefrontal cortices of the female mice that were prenatally exposed to triclocarban exhibited decreased protein levels of ESR1, ARNT, and CAR (Figure 1D). The levels of ESR1 and ARNT were reduced by approximately 40% relative to the control. Additionally, in these animals, CAR protein levels were reduced by 27%. The protein levels of other studied receptors and cytochromes remained unchanged.

### 2.2. Prenatal Exposure to Triclocarban Did Not Affect the mRNA or Protein Expression of Apoptosis-Related Factors Other Than BCL2

**Figure 2:**
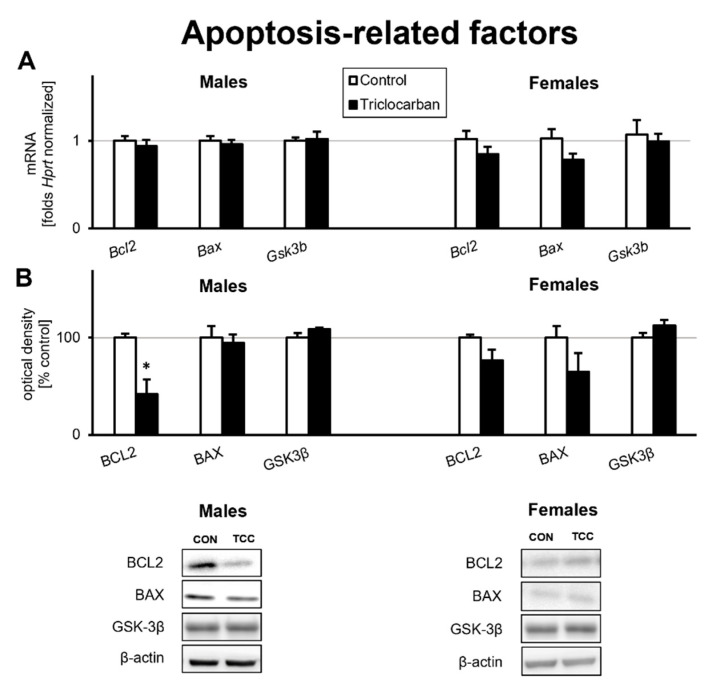
Triclocarban inhibited BCL2 protein expression in one-month-old male mice prenatally exposed to triclocarban. The mRNA expression level (**A**) and protein expression level (**B**) of apoptosis-related factors are presented. Each bar represents the mean ± SEM of a group of samples. Each control or experimental group consisted of animals from eight different litters, and the samples were collected from six animals per group. All results are expressed as mean ± SEM. * *p* < 0.05 versus the control animals.

The prefrontal cortices of one-month-old mice that were prenatally exposed to triclocarban expressed levels of *Bcl2*, *Bax*, and *Gsk3b* mRNAs that were similar to those observed in the control animals (Figure 2A).

In one-month-old male mice that were prenatally exposed to triclocarban, the protein level of BCL2 was reduced by over 60% compared to the control, but the protein levels of BAX and GSK3β remained unchanged. In one-month-old female mice, prenatal exposure to triclocarban did not change the protein levels of apoptosis-related factors (Figure 2B).

### 2.3. Prenatal Exposure to Triclocarban Did Not Affect the mRNA or Protein Expression of Autophagy-Related Factors Other Than Atg7 mRNA and MAP1LC3AB

**Figure 3:**
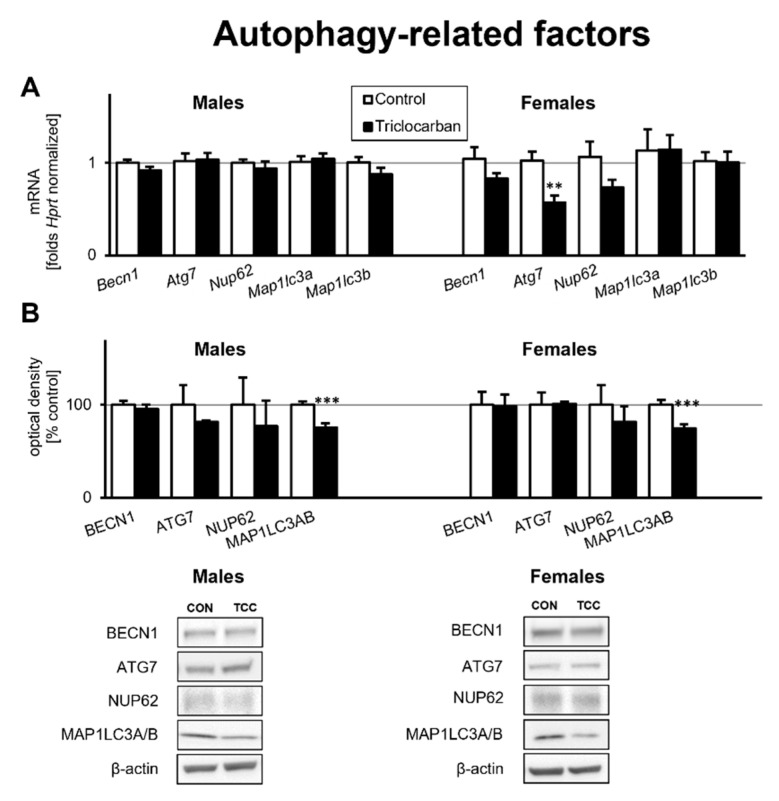
Triclocarban inhibited *Atg7* gene expression in one-month-old female mice and MAP1LC3AB expression in mice of both sexes prenatally exposed to triclocarban. The mRNA expression level (**A**) and protein expression level (**B**) of autophagy-related factors are shown. Each bar represents the mean ± SEM of a group of samples. Each control or experimental group consisted of animals from eight different litters, and the samples were collected from six animals per group. All results are expressed as mean ± SEM. ** *p* < 0.01 and *** *p* < 0.001 versus the control animals.

Prenatal exposure to triclocarban did not cause significant changes in the mRNA expression of autophagy-related factors except in the case of *Atg7* mRNA; its mRNA expression was reduced by 0.4-fold (44% decrease) in the prefrontal cortices of one-month-old female mice compared to the control (Figure 3A). mRNA expression of other genes encoding autophagy-related factors remained at the control level.

Western blot analyses demonstrated that the prefrontal cortices of one-month-old mice that were prenatally exposed to triclocarban exhibited unaltered levels of autophagy-related proteins such as BECN1, ATG7, and NUP62 compared to the control animals (Figure 3B). However, the protein level of MAP1LC3AB was decreased by 25% in male and female mice that had been prenatally exposed to triclocarban.

### 2.4. Prenatal Exposure to Triclocarban Caused Sex-Specific Disruption of the Methylation Statuses of Estrogen Receptor Genes

**Figure 4:**
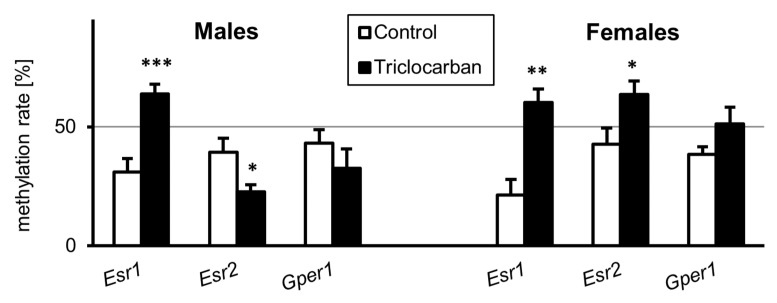
Prenatal exposure to triclocarban dysregulated DNA methylation of ER-specific genes in the prefrontal cortices of one-month-old animals. Each bar represents the mean ± SEM of a group of samples. Each control or experimental group consisted of animals from eight different litters, and the samples were collected from six animals per group. All results are expressed as mean ± SEM. * *p* < 0.05, ** *p* < 0.01, and *** *p* < 0.001 versus the control animals.

To extend our analysis of triclocarban-evoked changes in the expression levels of estrogen receptors, we analyzed the methylation statuses of specific genes, i.e., *Esr1*, *Esr2*, and *Gper1*, in the brains of one-month-old mice. In one-month-old male mice, prenatal exposure to triclocarban was associated with an approximately 2-fold increase in the methylation of *Esr1* and a 0.5-fold decrease in the methylation of *Esr2*, but it did not change the methylation status of the *Gper1* gene (Figure 4). In the brains of one-month-old female mice that were prenatally exposed to triclocarban, there was a nearly 3-fold increase in *Esr1* methylation and a 1.5-fold increase in *Esr2* methylation. In female brains, the level of methylation of *Gper1* remained unchanged.

### 2.5. Prenatal Exposure to Triclocarban Caused Sex-Dependent Alterations in Global DNA Methylation

**Figure 5:**
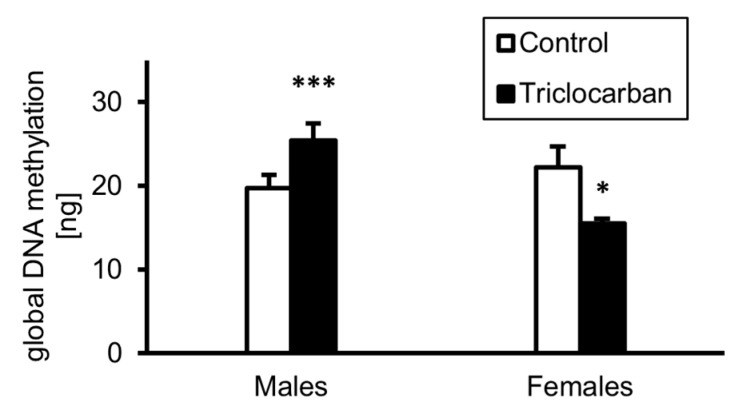
Prenatal exposure to triclocarban dysregulated global DNA methylation in the prefrontal cortices of one-month-old animals of both sexes. Each bar represents the mean ± SEM of a group of samples. Each control or experimental group consisted of animals from eight different litters, and the samples were collected from six animals per group. All results are expressed as mean ± SEM. * *p* < 0.05 and *** *p* < 0.001 versus the control animals.

Prenatal exposure to triclocarban changed the methylation status of the DNA in the prefrontal cortices of one-month-old mice (Figure 5). In control male mice, the level of DNA methylation reached a value of approximately 20 ng per 50 ng of DNA sample; this increased to more than 25 ng after prenatal exposure to triclocarban. In control female mice, global DNA methylation occurred at the level of approximately 22 ng per 50 ng of DNA sample, but prenatal treatment with triclocarban reduced the amount of methylated DNA to approximately 15 ng.

### 2.6. Triclocarban Dysregulated the Expression of Neurogenesis- and Neurotransmitter-Related Genes in One-Month-Old Mice That Were Prenatally Exposed to Triclocarban

#### 2.6.1. Triclocarban Dysregulated the Expression of Neurogenesis-Related Genes in One-Month-Old Male and Female Mice That Were Prenatally Exposed to the Chemical

**Figure 6:**
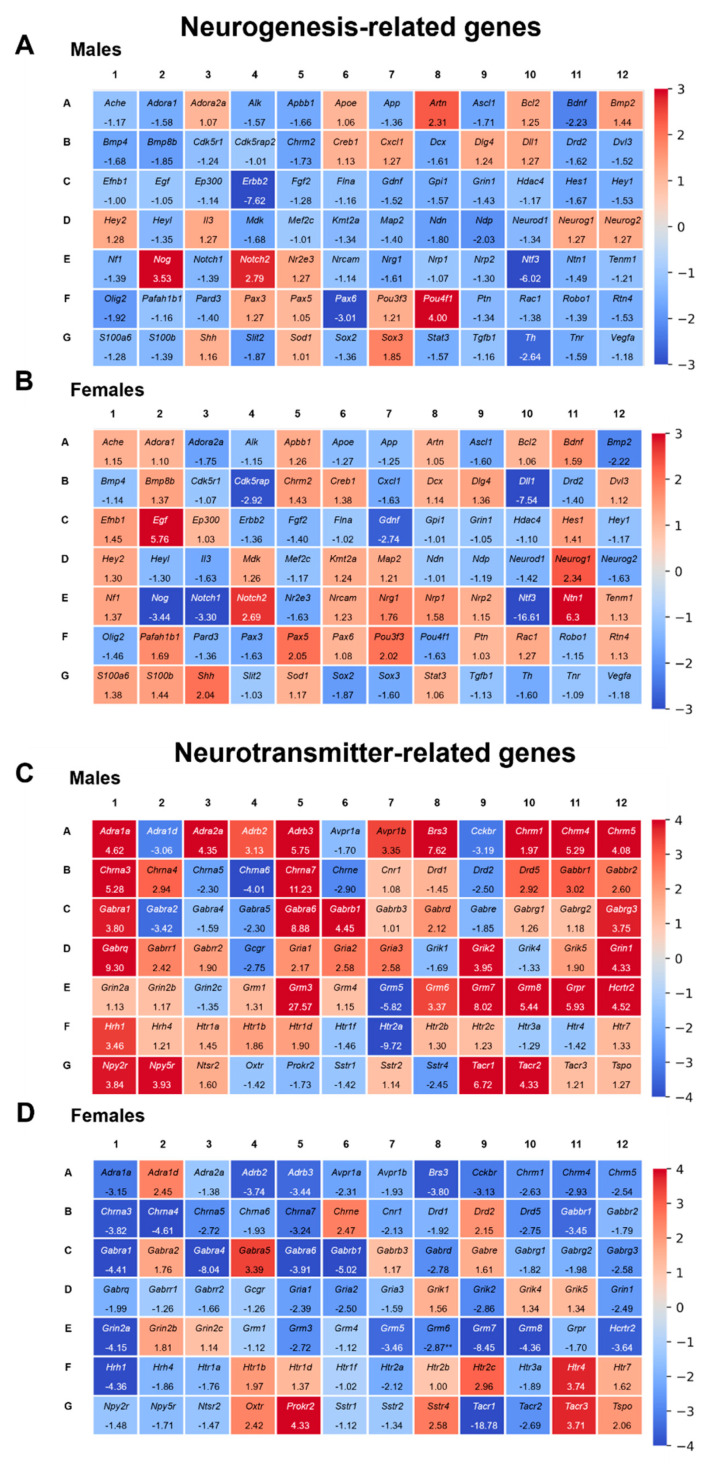
Prenatal exposure to triclocarban dysregulated the expression of neurogenesis- (**A**,**B**) and neurotransmitter-related (**C**,**D**) genes in the prefrontal cortices of one-month-old mice as determined by microarray analyses. The figure shows representative heatmaps of various genes in males and females. Downregulated genes are shown in red whereas upregulated genes are shown in blue.

To profile the expression of neurogenesis-related genes in one-month-old male mice that had been prenatally exposed to triclocarban, we used microarrays. A total of 84 key genes were analyzed using the RT^2^ Profiler™ PCR Array. Exposure to triclocarban dysregulated neurogenesis in the prefrontal cortices of male mice; 32 genes (shown in blue) were downregulated i.a. *Bdnf*, *Gdnf* and 6 genes (shown in red) were upregulated i.a. *Notch2*, *Sox3* (Figure 6A). Triclocarban dysregulated the expression of neurogenesis-related genes in female brains; it downregulated 22 genes (shown in blue), e.g., *Bmp2*, *Neurog2*, and upregulated 15 genes (shown in red), e.g., *Egf*, *Nrg1* (Figure 6B). A list of downregulated and upregulated genes is presented as a table in supplementary material (Supplementary Material Table S1, part a).

In mice of both sexes, triclocarban decreased the expression of *Ascl1*, *Drd2*, *Gdnf*, *Ntf3*, *Olig2*, and *Th* but increased the expression of *Notch2*. Among the triclocarban-affected genes, *Bdnf*, *Bmp2*, *Chrm2*, *Hes1*, *Nog*, *Nrg1*, *Ntn1*, *Pou4f1*, and *Sox3* were regulated in an opposite manner in males and females.

#### 2.6.2. Triclocarban Dysregulated the Expression of Neurotransmitter-Related Genes in One-Month-Old Male and Female Mice That Were Prenatally Exposed to the Chemical

In one-month-old male mice that were prenatally exposed to triclocarban, analysis of the expression profiles of 84 neurotransmission-related genes revealed that 54 genes were differentially expressed in the animals’ prefrontal cortices. Prenatal exposure to triclocarban resulted in the upregulation of 38 genes, e.g., *Grin1*, *Gria1*, and the downregulation of 16 genes, e.g., *Htr2a*, *Htr3a* (Figure 6C). In one-month-old female mouse prefrontal cortices, prenatal exposure to triclocarban caused a change in the expression levels of 49 genes, of which 11 were upregulated, e.g., *Adra1d*, *Htr2c*, and 38 were downregulated, e.g., *Gabra1*, *Grm3* (Figure 6D). A list of downregulated and upregulated genes is presented as a table in supplementary material (Supplementary Material Table S1, part b).

In mice of both sexes, triclocarban decreased the expression of *Avpr1a*, *Cckbr*, *Chrna5*, *Grm5*, and *Htr2a*. Among the triclocarban-affected genes, *Adra1a*, *Adra1d*, *Adra2a*, *Adrb2*, *Adrb3*, *Brs3*, *Chrm1*, *Chrm4*, *Chrm5*, *Chrna3*, *Chrna4*, *Chrna7*, *Chrne*, *Drd2*, *Drd5*, *Gabbr1*, *Gabra1*, *Gabra5*, *Gabra6*, *Gabrb1*, *Gabrd*, *Gabrg3*, *Gria1*, *Gria2*, *Grik2*, *Grin1*, *Grm3*, *Grm6*, *Grm7*, *Grm8*, *Hcrtr2*, *Hrh1*, *Htr4*, *Sstr4*, *Tacr1*, and *Tacr2* were divergently regulated in males and females.

### 2.7. Enrichment Analyses—GO Biological Process Analysis of Downregulated Genes in the Prefrontal Cortices of One-Month-Old Males and Females That Were Prenatally Exposed to Triclocarban

**Figure 7:**
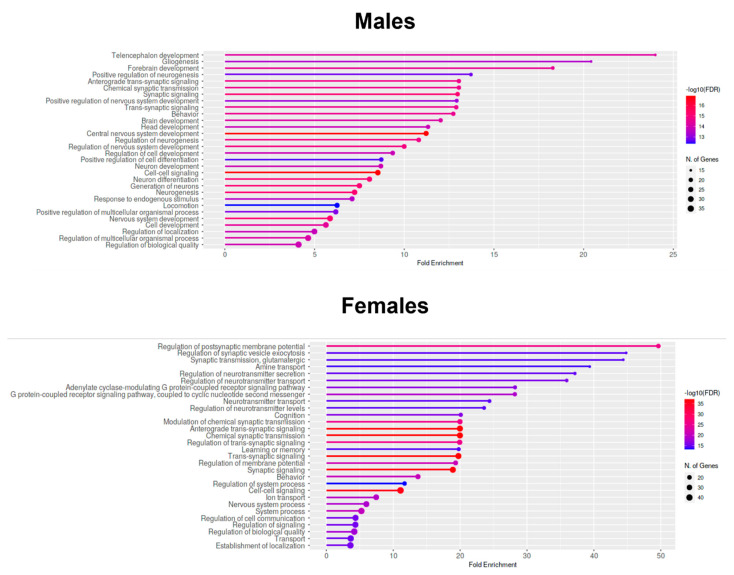
Enrichment analyses—GO Biological Process analysis of downregulated genes in the prefrontal cortices of one-month-old males and females that were prenatally exposed to triclocarban.

Web-based analysis applications ShinyGO and g:Profiler were used to perform an enrichment analysis of genes that were upregulated/downregulated in the prefrontal cortices of one-month-old mice in response to triclocarban. Here, in the main text, we provide the GO Biological Process analysis of downregulated genes in the prefrontal cortices of males and females (Figure 7). According to the analysis, the expression of downregulated genes in one-month-old male mice was highly associated with telencephalon development, gliogenesis, and forebrain development. In case of one-month-old female mice, the downregulated genes were mainly involved in synaptic development, particularly in the regulation of postsynaptic membrane potential, regulation of synaptic vesicle exocytosis, and glutamatergic synaptic transmission.

Other enrichment analyses (including g:GOSt, GO Cellular Component, GO Molecular Function, Curated Reactome, and KEGG) of upregulated and downregulated genes in the prefrontal cortices of one-month-old mice that were prenatally exposed to triclocarban are presented as Supplementary Materials S3–S6.

### 2.8. Triclocarban Crossed the Blood–Brain Barrier In Vitro

**Figure 8:**
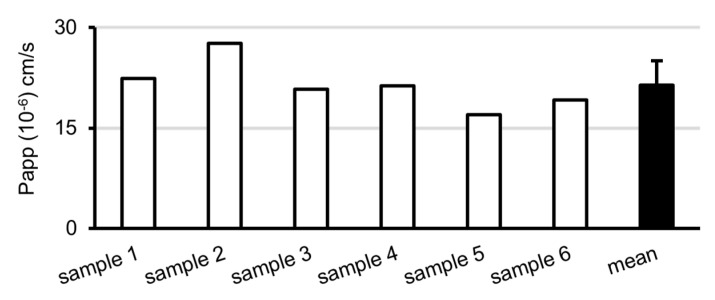
Triclocarban crossed the in vitro blood–brain barrier. The permeability coefficient (Papp) value (×10^−6^ cm/s) for triclocarban was estimated at approximately 21, indicating that the compound has a very good ability to penetrate the BBB. The number of replicates was 6.

The evaluation of triclocarban (10 µM) permeability with the use of the BBB Kit™ (RBT-24) revealed that the compound has the ability to cross the BBB (Figure 8). The permeability coefficient (Papp) value (×10^−6^ cm/s) of triclocarban was estimated at approximately 21; this indicates that the compound has a very good ability to penetrate the BBB, since, according to the kit manufacturer, compounds with Papp <2, 2–10, 10–20, and >20 are classified as having very low, low, good, and very good permeability, respectively.

## 3. Discussion

This in vivo study verified our previously formulated hypothesis that triclocarban is a neurodevelopmental risk factor and assessed the range of brain abnormalities and signaling dysregulations that occur following the prenatal exposure of mice to triclocarban. The estimated BBB permeability of triclocarban indicated that the compound has a very good ability to penetrate the barrier. We demonstrated for the first time that mRNA and protein expression of ESR1 was impaired in the brains of one-month-old mice that had been prenatally exposed to environmentally relevant doses of triclocarban. Decreased expression levels of *Esr1*/ESR1, as well as hypermethylation of the *Esr1* gene, were observed in both male and female brain cortices. Previously, we observed a similar effect in vitro, in which, exposure to triclocarban inhibited the expression of *Esr1*/ESR1 in mouse brain neurons in primary culture in a manner that was correlated with gene hypermethylation [9]. Triclocarban was also shown to decrease ERα/ESR1 expression in MCF-7 cells [19] and to downregulate ER signaling by inhibiting aromatase activity in JEG-3 cells [20]. Intriguingly, other studies have demonstrated stimulation of ER signaling in response to triclocarban, including an increase in the brain-specific expression of aromatase in zebrafish embryos [21] and activation of ERα/ESR1 detected in receptor-based assays [22]. These differences could be due to the triclocarban-evoked impairment of puberty as reported by Mandour et al. (2021) in female rats that were exposed to the compound in utero or during lactation [23]. One can assume that the effects of prenatal exposures to triclocarban observed in our study could be in part caused by comparing the control and experimental groups, which were exactly at the same age but differed with respect to the stage/advancement of puberty.

In the present study, in addition to impairment of *Esr1*/ESR1 expression, prenatal exposure to triclocarban diminished the mRNA expression levels of *Esr2*, *Gper1*, *Ahr*, *Arnt*, *Cyp19a1*, *Cyp1a1*, and *Atg7*, and the protein levels of CAR, ARNT, and MAP1LC3AB in female brains and decreased the protein levels of BCL2, ARNT, and MAP1LC3AB in male brains. The mRNA and protein expression of BAX, GSK3β, BECN1, and NUP62 remained unchanged. Therefore, taking into account mRNA and protein expression that have been changed in response to prenatal exposures to triclocarban, one may suggest that female mice are more vulnerable and susceptible to the disrupting effects of triclocarban than are male mice, particularly with respect to the expression of ER-related genes other than *Esr1* (*Esr2*, *Gper1*, *Cyp19a1*), AHR-related genes (*Ahr*, *Arnt*, *Cyp1a1*), and the autophagy-related *Atg7* gene. It is well known that the expression of apoptosis-related genes, e.g., *Bcl2*, is strongly controlled by ESR1 [24]. Moreover, the latest data have shown that overexpressing ESR1 results in significantly enhanced expression of autophagy-related proteins MAP1LC3 and ATG7 in human chondrocytes in vitro [25]. The ER and AHR signaling pathways are known to interact closely and to engage in crosstalk [26,27]. We suggest that in mouse brains, an impairment of ESR1, i.e., the receptor which is known to affect gene transcription, could modify expression levels of apoptosis- and autophagy-related factors, possibly due to DNA methylation, and we found this to be divergently regulated in triclocarban-treated female and male mice. Because mice were matched according to age and not puberty, triclocarban-induced alterations in female mice may appear to be more pronounced than in males; however, these differences may not be as prominent if mice were matched according to puberty.

The observed decreases in *Atg7* mRNA and protein levels of CAR, ARNT, BCL2, and MAP1LC3AB confirm the disruptive impact of triclocarban on AHR and CAR expression levels and indicate that apoptosis and autophagy may be involved in the brain response to triclocarban. Male-specific downregulation of antiapoptotic BCL2 and sex-independent downregulation of autophagy-related MAP1LC3AB suggest that apoptosis predominates in males whereas autophagy is impaired in both males and females. Xu et al. [28] reported abnormal expression of apoptosis-related genes in zebrafish embryos following exposure to triclocarban; this is consistent with the downregulation of BCL2 in male brains observed in the present study. Our previous in vitro study showed that triclocarban-induced apoptosis in mouse primary neurons is mediated by AHR and CAR and that impairment of autophagy involves the downregulation of *Atg7* [8]. In the present research, CAR expression was decreased in female brains, suggesting that the vulnerability of cells in the female brain to induction of apoptosis following prenatal exposure to triclocarban was diminished. Taking this into account, one may assume that these females suffered from impaired autophagy rather than from induced apoptosis, although abnormalities in autophagy can increase apoptosis.

In this study, we demonstrated that prenatal exposure to triclocarban disrupted epigenetic status via sex-specific alterations in the methylation levels of both global DNA and estrogen receptor genes. In male brains, global DNA hypermethylation corresponded to *Esr1* gene hypermethylation but not to *Esr2* gene hypomethylation. In female brains, global DNA hypomethylation did not correlate with hypermethylation of the *Esr1* and *Esr2* genes, suggesting the involvement of other triclocarban-regulated genes in females. Indeed, specific microarrays and enrichment analyses showed sex-specific dysregulation of neurogenesis- and neurotransmitter-related genes and processes. In males, triclocarban dysregulated mainly neurogenesis-related pathways, whereas in females, the compound dysregulated mainly neurotransmitter-related pathways. The microarrays followed by enrichment analyses showed that in one-month-old male mice, the expression of downregulated genes (such as *Bdnf*, *Gdnf*, *Map2*) was highly associated with telencephalon development, gliogenesis, and forebrain development. As for one-month-old female mice, it appeared that the downregulated genes (such as *Gabra1*, *Gria1*, *Grin2a*) were mainly involved in synaptic development, particularly in the regulation of postsynaptic membrane potential, regulation of synaptic vesicle exocytosis, and glutamatergic synaptic transmission.

## 4. Materials and Methods

### 4.1. Material

An imprint methylated DNA quantification kit and triclocarban (3,4,4′-trichlorocarbanilide) were provided by Sigma-Aldrich (St. Louis, MO, USA). Mini-PROTEAN TGX precast gels (10% and 7.5%) were purchased from Bio-Rad Laboratories (Hercules, CA, USA). The cDNA reverse transcription kit–high-capacity cDNA reverse transcription kit, RNAlater, TaqMan gene expression master mix, and TaqMan probes for the specific receptor genes *Esr1*, *Esr2*, *Gper1*, *Car*, *Ahr*, and AHR-related *Arnt*; the cytochrome genes *Cyp19a1* and *Cyp1a1*; the apoptosis-related genes *Bcl2*, *Bax* and *Gsk3b*; and the autophagy-related genes *Becn1*, *Map1lc3a*, *Map1lc3b*, *Nup62*, and *Atg7* were obtained from Thermo Fisher Scientific (Waltham, MA, USA). PVDF membranes were obtained from Merck Millipore (Billerica, MA, USA). BM chemiluminescence blotting substrate and lysis buffer were obtained from Roche Diagnostics GmbH (Mannheim, Germany). The RNeasy Mini Kit, RT^2^ Profiler PCR arrays, RT^2^ SYBR Green qPCR Mastermix, RT^2^ First Strand Kit, and EpiTect MethyLight PCR kit were obtained from Qiagen (Hilden, Germany). The EZ DNA Methylation-Gold kit and the Quick-gDNA™ MicroPrep kit were from Zymo Research (Irvine, CA, USA). Mouse IgG kappa binding protein conjugated to horseradish peroxidase (sc-516102), donkey anti-goat antibody conjugated to horseradish peroxidase (sc-2020), rabbit polyclonal anti-BECN1 antibody (sc-11427), mouse monoclonal anti-β-actin antibody (sc-47778), mouse monoclonal anti-MAP1LC3AB antibody (sc-398822), mouse monoclonal anti-nucleoporin p62 antibody (sc-48373), rabbit polyclonal anti-GPER1 antibody (sc-134576), mouse monoclonal anti-CYP1A1 antibody (sc-25304), rabbit polyclonal anti-GSK-3β antibody (sc-9166), rabbit polyclonal anti-ESR1 antibody (sc-7207), goat polyclonal anti-ESR2 antibody (sc-6822), goat polyclonal anti-AHR antibody (sc-8088), rabbit polyclonal anti-ARNT antibody (sc-5580), mouse monoclonal anti-BCL-2 antibody (sc-7382), mouse monoclonal anti-BAX antibody (sc-7480), and mouse monoclonal anti-ATG7 antibody (sc-376212) were purchased from Santa Cruz Biotechnology Inc. (Santa Cruz, CA, USA). A polyclonal rabbit anti-constitutive androstane receptor antibody (ab-228767) and rabbit polyclonal anti-aromatase antibody (ab-18995) were purchased from ABCAM (Cambridge, UK). Goat anti-rabbit HRP-conjugated secondary antibody (31460) was purchased from Invitrogen (Waltham, MA, USA). The BBB Kit™ was purchased from PharmaCo-Cell Company Ltd. (Nagasaki, Japan). All other chemicals were of analytical or laboratory grade and were purchased from standard suppliers.

### 4.2. Animals

The 15 pregnant Albino Swiss mice (Charles River Laboratories, Sulzfeld, Germany) used in this study were individually maintained in house cages. At the age of 21 days, the male and female offspring of the mice were separated and housed at a density of 8 animals per cage (57 × 35 × 20 cm). The mice were maintained at 21 ± 1 °C in an atmosphere with 40–50% humidity with free access to water and food. The artificial photoperiod, with lights on at 7 a.m., mimicked the natural sequence of day and night. The care provided to the animals conformed to governmental guidelines, and all experimental protocols were approved by the Committee for Laboratory Animal Welfare and by the Ethics Committee of the Maj Institute of Pharmacology PAS in Krakow, Poland (resolution no. 112/2016). Efforts were made to minimize the animals’ suffering and the number of animals used. The procedures met the requirements of the European Communities Council Directive for the Care and Use of Laboratory Animals (86/609/EEC).

### 4.3. Treatment

Triclocarban was administered to pregnant Swiss mice at a dose of 5 mg/kg at 15–18 days of gestation. The time and duration of prenatal exposure were similar to those used in a previous study [29]. The dose used did not cause any visible adverse effects and was environmentally relevant. The chosen concentration of triclocarban is environmentally relevant since it is similar to the levels that have been found in animal tissues [30,31,32]. Moreover, triclocarban has been found in white matter in the human brain at a concentration of 5 µg/kg [7]. The mean human topical exposure to triclocarban from soap use has been estimated to be 70 ± 15 mg triclocarban; this corresponds to a dose of 1 mg/kg body weight based on 40 mg/m^2^ body surface area [33]. Triclocarban was dissolved in corn oil and injected subcutaneously in a volume of 5 mL/kg body weight as previously described [29]. Control pregnant mice were injected with equal amounts of corn oil. The experiments were performed on exactly age-matched animals derived from 15 litters that were randomly and separately grouped into males and females.

### 4.4. Sampling

One-month-old animals were anesthetized in a CO_2_ atmosphere and sacrificed by spinal cord rupture. The animals’ brains were rapidly removed and placed on an ice-cold porcelain plate. The prefrontal cortex of each brain was dissected according to its anatomical borders using a visually guided procedure. The prefrontal cortex was chosen for analysis because this brain structure is known to be affected by prenatal exposure to environmentally pervasive chemicals [29,34]. The collected tissue was maintained at a temperature of −70 °C until analyses.

### 4.5. qPCR Analyses

In the prefrontal cortices of one-month-old animals, the expression levels of mRNAs specific to genes that encode the estrogen receptors *Esr1*, *Esr2*, and *Gper1*; the xenobiotic receptors *Ahr* and *Car*; the receptor-regulated factors *Arnt*, *Cyp19a1*, and *Cyp1a1*; the apoptosis-related factors *Bcl2*, *Bax*, and *Gsk3b*; and the autophagy-related factors *Becn1*, *Map1lc3a*, *Map1lc3b*, *Nup62*, and *Atg7* were analyzed.

From the brain tissue of each mouse, total RNA (approx. 3.5 µg per sample) was extracted using a Qiagen RNeasy mini kit (Hilden, Germany). The amounts of RNA were determined spectrophotometrically at 260 nm and 260/280 nm (ND/1000 UV/Vis; Thermo Fisher NanoDrop, Waltham, MA, USA). Reverse transcription (RT) and quantitative polymerase chain reaction (qPCR) were performed using the CFX96 Real-Time System (Bio-Rad, Hercules, CA, USA) as previously described [34,35,36]. High-capacity cDNA reverse transcription kits, the TaqMan Gene Expression Master Mix kit and TaqMan probes (Thermo Fisher Scientific, Waltham, MA, USA), were used to transcribe and amplify the specific genes. *Hprt*, the gene encoding hypoxanthine phosphoribosyltransferase, was used as the reference gene, and its expression was estimated using the RefFinder web-based comprehensive tool, which integrates the currently available major computational programs (geNorm, NormFinder, BestKeeper, and the comparative Delta-Ct method) to compare and rank the tested candidate reference genes [37].

### 4.6. Measurement of DNA Methylation of Specific Genes

High-quality, ultrapure genomic DNA was extracted from the prefrontal cortices of one-month-old mice using a Quick-gDNA™ MicroPrep kit (Zymo Research, Irvine, CA, USA) as previously described [38]. The amount of DNA obtained from each sample was spectrophotometrically determined by measuring the absorbance of the sample at 260 nm and at 280 nm (ND/1000 UV/Vis; Thermo Fisher NanoDrop, Waltham, MA, USA). This was followed by the complete bisulfite conversion of GC-rich DNA through the use of the EZ DNA Methylation-Gold™ kit (Zymo Research, Irvine, CA, USA), which integrates DNA denaturation and bisulfite conversion into one step. The samples were then eluted in a 10 µL volume and stored at −80 °C until needed. qPCR was performed using the EpiTect MethyLight PCR kit (Qiagen, Hilden, Germany). TaqMan probes were designed specifically for the bisulfite-converted DNA sequences. They included a set representing fully methylated and fully unmethylated probes for the *Esr1*, *Esr2*, and *Gper1* promoters and an internal reference set for the *Hprt* gene to control for input DNA.

For the EpiTect MethyLight assays, the specific TaqMan probes contained FAM™ as the reporter dye. The degree of methylation of each sample was calculated by taking the threshold cycles determined for each dye: percentage of methylation [%]: C_meth_ = 100/[1 + 2(ΔCt_meth_ − ΔCt_unmeth_)] according to previously published work [39].

### 4.7. Measurement of Global DNA Methylation

To determine the methylation status of the DNA in the prefrontal cortices of one-month-old mice that had been prenatally exposed to triclocarban, an Imprint Methylated DNA Quantification Kit was used. The methylated DNA was identified by capture and detection antibodies in the following way. First, purified DNA was added to a 96-well plate, allowed to bind, and incubated with the capture antibody. Developing solution was then added, and the wells were monitored for color changes and quantified colorimetrically as previously described [9]. The amount of methylated DNA was determined spectrophotometrically (ND/1000 UV/Vis; Thermo Fisher NanoDrop, Waltham, MA, USA), and the relative global methylation levels were calculated based on the absorbance of the samples at 450 nm per 50 ng of DNA sample.

### 4.8. Profiling Neurogenesis-Related Genes and Neurotransmitter-Related Genes Using Microarray Assays

To obtain high-quality RNA from mouse prefrontal cortices, an RNeasy mini kit (Qiagen, Hilden, Germany) was used, and the procedure was conducted according to the manufacturer’s protocol as previously described [40]. The RNA quantity was determined by measuring the absorbance of the sample at 260 nm and at 280 nm (ND/1000 UV/Vis; Thermo Fisher NanoDrop, Waltham, MA, USA). Reverse transcription to cDNA was performed using the RT^2^ First Strand kit (Qiagen, Hilden, Germany), and the signaling pathways were analyzed using the RT^2^ Profiler^TM^ PCR Array system (Qiagen, Hilden, Germany). Both RT and qPCR profiling were performed in a CFX96 Real-Time System (Bio-Rad, Hercules, CA, USA). Web-based software (RefFinder) was used to analyze the Ct values. The reference genes were *Actb* (β-actin), *B2m* (β-2 microglobulin), *Gapdh* (glyceraldehyde-3-phosphate dehydrogenase), *Guspb* (beta-glucuronidase), and *Hsp90ab1* (heat shock protein 90 alpha (cytosolic), class B member 1).

### 4.9. Western Blot Analysis

To prepare samples for Western blot analyses, mouse prefrontal cortices were lysed, sonicated, and centrifuged for 20 min. Individual samples containing 40 µg of total protein were reconstituted in appropriate amounts of sample buffer, denatured, and separated on 7.5% SDS-polyacrylamide gels using a Bio-Rad Mini-Protean II Electrophoresis Cell as previously described [36,41,42]. The separated proteins were electrotransferred to PVDF membranes using a Bio-Rad Mini Trans-Blot apparatus, and nonspecific binding sites were blocked with 5% dry milk and 0.2% Tween-20 in 0.02 M TBS (Tris-buffered saline) for 2 h with shaking. In the next step of the procedure, the membrane was incubated for 12 h at 4 °C with one of the following primary antibodies diluted in TBS/Tween 20: rabbit polyclonal anti-BECN1 antibody (1:700), mouse monoclonal anti-β-actin antibody (1:3500 and 1:4000), mouse monoclonal anti-MAPLC3AB antibody (1:700), mouse monoclonal anti-NUP62 antibody (1:500), rabbit polyclonal anti-GPR30 antibody (1:200), mouse monoclonal anti-CYP1A1 antibody (1:700), rabbit polyclonal anti-GSK-3β antibody (1:500), rabbit polyclonal anti-ERα antibody (1:300), goat polyclonal anti-ERβ antibody (1:300), goat polyclonal anti-AHR antibody (1:150 and 1:200), rabbit polyclonal anti-ARNT antibody (1:1000), mouse monoclonal anti-BCL2 antibody (1:1000), mouse monoclonal anti-BAX antibody (1:1000), mouse monoclonal anti-ATG7 antibody (1:1000), polyclonal rabbit anti-CAR (1:500), and rabbit polyclonal anti-aromatase antibody (1:500). After being washed, the membranes were incubated for 1 h with horseradish peroxidase–conjugated IgG (goat anti-rabbit, donkey anti-goat and mouse IgG kappa binding protein) diluted 1:1000 (or 1:3500 in the case of β-actin) in TBS/Tween 20. To confirm that equal amounts of denatured protein had been loaded onto the gels, the membranes were stripped and reprobed with an anti-β-actin antibody. The protein-specific signals were developed by chemiluminescence, visualized with a Luminescent Image Analyzer Fuji-Las 4000 (Fuji, Japan), and normalized to β-actin specific band. An image analyzer (Science Lab, Multi Gauge V3.0) was used to quantify the immunoreactive bands.

### 4.10. Measurement of Blood–Brain Barrier Permeability

To evaluate the ability of triclocarban (10 µM) to cross the BBB, a BBB Kit™ (PharmaCo-Cell Company Ltd., Nagasaki, Japan) consisting of primary cultures of brain capillary endothelial cells, pericytes, and astrocytes obtained from Wistar rats was used [34,43,44]. The procedure was performed according to the manufacturer’s protocol: triclocarban was added to the upper (luminal, blood-side) insert for 30 min, and the samples were then collected from the lower (abluminal, brain-side) compartment. To each collected sample of 900 µL, 300 µL of brine and 500 µL of ethyl acetate were added. The samples were then stirred to separate the phases, and the triclocarban concentrations were evaluated by measuring, in triplicate, the total ion current (TIC) for the molecular mass of triclocarban on a TQD Waters mass spectrometer with ESI+ ionization coupled to an H-class UPLC. The samples were separated on an ACQUITY UPLC BEH C18 column (1.7 μm, 2.1 × 50 mm) using a 4 min gradient (0.3 mL/min) increasing from 80% H_2_O–20% ACN to 100% ACN at 2.5 min, followed by 100% ACN for 0.5 min and decreasing back to 80% H_2_O–20% ACN at 4 min. Triclocarban was eluted at 3.3 min. The ionization parameters were as follows: cone voltage 30 V, capillary voltage 3.95 kV, extractor voltage 2.2 V, RF 0.1 V, source temperature 150 °C, and desolvation temperature 250 °C. The number of replicates was 5. The apparent permeability coefficient (Papp) was calculated as follows:

$$Papp\;\left(\frac{cm}{min}\right)=\frac{Va}{A\times \left[C\right]luminal}\times \frac{\Delta \;\left[C\right]abluminal}{\Delta t}$$

where *A* represents the culture area (cm^2^), *Va* represents the volume of assay buffer on the luminal side, Δ[*C*] *abluminal* is the concentration of sample on the abluminal side, [*C*] *luminal* is the initial concentration of sample added to the luminal side, and Δ*t* is the duration of the assay in minutes.

### 4.11. Data Analysis

The raw data obtained in this study are expressed in the following ways: fluorescence units per 350 ng of RNA (qPCR), mean optical density per 40 µg of protein (Western blot), ng of methylated DNA per 50 ng of DNA sample (global DNA methylation), 150 ng of DNA sample for specific gene methylation, and 500 ng RNA for microarray analysis. Each control or experimental group consisted of animals from eight different litters, and the samples were collected from six animals in each group. All results are expressed as mean ± SEM. To compare the effects of triclocarban in male and female mice and its effect on various receptors, the results obtained in the DNA methylation and Western blot analyses are presented as percentages of the controls. Levene’s test of the homogeneity of variances was used to determine the overall significance and was followed by one-way analysis of variance (ANOVA). The differences between the control and experimental groups were assessed using a post hoc Newman–Keuls test. Significant differences are indicated as follows: * *p* < 0.05; ** *p* < 0.01; and *** *p* < 0.001.

### 4.12. Enrichment Analysis

Enrichment analysis and conversions of gene lists have been made using web-based analysis applications—ShinyGO and g: Profiler. ShinyGO is an in-depth analysis of gene lists, with graphical visualization of enrichment, pathway, gene characteristics, and protein interactions using which enrichment analysis can link these gene lists with underlying molecular pathways and functional categories such as gene ontology (GO) and other databases such as KEGG and STRING [45]. g:Profiler is a collection of tools that are commonly used in standard pipelines of biological-entity (gene/protein)-centered computational analysis. Here, we used g:GOSt to perform the functional enrichment analysis of gene lists [46]. g:GOSt performs functional enrichment analysis known as over-representation analysis (ORA) on the input gene list. It maps genes to known functional information sources and detects statistically significantly enriched terms.

## 5. Conclusions

In conclusion, our original data identify triclocarban as a neurodevelopmental risk factor that particularly targets ESR1 and affects apoptosis- and autophagy-related factors in sex-specific ways. Microarray and enrichment analyses showed that, in males, triclocarban dysregulated mainly neurogenesis-related genes, whereas, in females, the compound dysregulated mainly neurotransmitter-related genes. Since the above processes and signaling pathways are crucial for proper brain development and neurotransmission, prenatal exposure to triclocarban could predispose to neurodevelopmental abnormalities, which would be revealed in the postnatal mammalian brain; thus representing a fetal basis for adult-onset disease.

## Data Availability

The data generated during this study is available upon request.

## Supplementary Materials

The following are available online at https://www.mdpi.com/article/10.3390/ijms222313121/s1.

## Abbreviations

ADHD	Attention-deficit hyperactivity disorder
*Ahr*/AHR	Aryl hydrocarbon receptor
*Arnt*/ARNT	Aryl hydrocarbon receptor nuclear translocator
ASD	Autism spectrum disorder
*Atg7*/ATG7	Autophagy-related 7
BBB	Blood–brain barrier
*Car*/CAR	Constitutive androstane receptor
ERs	Estrogen receptors
*Esr1*/ESR1	Estrogen receptor 1; estrogen receptor alpha (ERα)
*Esr2*/ESR2	Estrogen receptor 2; estrogen receptor beta (ERβ)
*Gper1*/GPER1	G-protein-coupled estrogen receptor 1; G-protein-coupled receptor 30 (GPR30)
*Map1lc3a/b*/MAP1LC3AB	Microtubule associated protein 1 light chain 3 alpha/beta
TCC	Triclocarban (3,4,4′-trichlorocarbanilide)

## References

[1] Yun H., Liang B., Kong D., Li X., Wang A. (2020). Fate, risk and removal of triclocarban: A critical review. J. Hazard Mater..

[2] Iacopetta D., Catalano A., Ceramella J., Saturnino C., Salvagno L., Ielo I., Drommi D., Scali E., Plutino M.R., Rosace G. (2021). The Different Facets of Triclocarban: A Review. Molecules.

[3] Bai X., Zhang B., He Y., Hong D., Song S., Huang Y., Zhang T. (2020). Triclosan and triclocarbon in maternal-fetal serum, urine, and amniotic fluid samples and their implication for prenatal exposure. Environ. Pollut..

[4] Kennedy R.C., Menn F.M., Healy L., Fecteau K.A., Hu P., Bae J., Gee N.A., Lasley B.L., Zhao L., Chen J. (2015). Early life triclocarban exposure during lactation affects neonate rat survival. Reprod. Sci..

[5] Aker A., McConnell R.E.R., Loch-Caruso R., Park S.K., Mukherjee B., Rosario Z.Y., Vélez-Vega C.M., Huerta-Montanez G., Alshawabkeh A.N., Cordero J.F. (2020). Interactions between chemicals and non-chemical stressors: The modifying effect of life events on the association between triclocarban, phenols and parabens with gestational length in a Puerto Rican cohort. Sci. Total Environ..

[6] Pycke B.F., Geer L.A., Dalloul M., Abulafia O., Jenck A.M., Halden R.U. (2014). Human fetal exposure to triclosan and triclocarban in an urban population from Brooklyn, New York. Environ. Sci. Technol..

[7] Van Der Meer T.P., Artacho-Cordón F., Swaab D.F., Struik D., Makris K.C., Wolffenbuttel B.H.R., Frederiksen H., Van Vliet-Ostaptchouk J.V. (2017). Distribution of Non-Persistent Endocrine Disruptors in Two Different Regions of the Human Brain. Int. J. Environ. Res. Public Health.

[8] Kajta M., Wnuk A., Rzemieniec J., Lason W., Mackowiak M., Chwastek E., Staniszewska M., Nehring I., Wojtowicz A.K. (2019). Triclocarban Disrupts the Epigenetic Status of Neuronal Cells and Induces AHR/CAR-Mediated Apoptosis. Mol. Neurobiol..

[9] Kajta M., Rzemieniec J., Wnuk A., Lasoń W. (2020). Triclocarban impairs autophagy in neuronal cells and disrupts estrogen receptor signaling via hypermethylation of specific genes. Sci. Total Environ..

[10] Dong N., Zhu J., Han W., Wang S., Yan Z., Ma D., Goh E.L.K., Chen T. (2018). Maternal methamphetamine exposure causes cognitive impairment and alteration of neurodevelopment-related genes in adult offspring mice. Neuropharmacology.

[11] La Barbera L., Vedele F., Nobili A., D’Amelio M., Krashia P. (2019). Neurodevelopmental Disorders: Functional Role of Ambra1 in Autism and Schizophrenia. Mol. Neurobiol..

[12] Fleming A., Rubinsztein D.C. (2020). Autophagy in Neuronal Development and Plasticity. Trends Neurosci..

[13] Hess J.L., Radonjić N.V., Patak J., Glatt S.J., Faraone S.V. (2020). Autophagy, apoptosis, and neurodevelopmental genes might underlie selective brain region vulnerability in attention-deficit/hyperactivity disorder. Mol. Psychiatry.

[14] Hwang W.J., Lee T.Y., Kim N.S., Kwon J.S. (2020). The Role of Estrogen Receptors and Their Signaling across Psychiatric Disorders. Int. J. Mol. Sci..

[15] Juricek L., Coumoul X. (2018). The Aryl Hydrocarbon Receptor and the Nervous System. Int. J. Mol. Sci..

[16] Oliviero F., Lukowicz C., Boussadia B., Forner-Piquer I., Pascussi J.M., Marchi N., Mselli-Lakhal L. (2020). Constitutive Androstane Receptor: A Peripheral and a Neurovascular Stress or Environmental Sensor. Cells.

[17] Corley M.J., Vargas-Maya N., Pang A.P.S., Lum-Jones A., Li D., Khadka V., Sultana R., Blanchard D.C., Maunakea A.K. (2019). Epigenetic Delay in the Neurodevelopmental Trajectory of DNA Methylation States in Autism Spectrum Disorders. Front. Genet..

[18] Aref-Eshghi E., Kerkhof J., Pedro V.P., Groupe D.I.F., Barat-Houari M., Ruiz-Pallares N., Andrau J.C., Lacombe D., Van-Gils J., Fergelot P. (2020). Evaluation of DNA Methylation Episignatures for Diagnosis and Phenotype Correlations in 42 Mendelian Neurodevelopmental Disorders. Am. J. Hum. Genet..

[19] Huang H., Du G., Zhang W., Hu J., Wu D., Song L., Xia Y., Wang X. (2014). The in vitro estrogenic activities of triclosan and triclocarban. J. Appl. Toxicol..

[20] Li H., Zhao Y., Chen L., Su Y., Li X., Jin L., Ge R.S. (2017). Triclocarban and Triclosan Inhibit Human Aromatase via Different Mechanisms. Biomed. Res. Int..

[21] Chung E., Genco M.C., Megrelis L., Ruderman J.V. (2011). Effects of bisphenol A and triclocarban on brain-specific expression of aromatase in early zebrafish embryos. Proc. Natl. Acad. Sci. USA.

[22] Li Z.L., Ueki K., Kumagai K., Araki R., Otsuki Y. (2014). Regulation of bcl-2 transcription by estrogen receptor-α and c-Jun in human endometrium. Med. Mol. Morphol..

[23] Mandour D.A., Aidaros A.A., Mohamed S. (2021). Potential long-term developmental toxicity of in utero and lactational exposure to Triclocarban (TCC) in hampering ovarian folliculogenesis in rat offspring. Acta Histochem..

[24] Liu M., Xie W., Zheng W., Yin D., Luo R., Guo F. (2019). Targeted binding of estradiol with ESR1 promotes proliferation of human chondrocytes in vitro by inhibiting activation of ERK signaling pathway. Nan Fang Yi Ke Da Xue Xue Bao.

[25] Yueh M.F., Li T., Evans R.M., Hammock B., Tukey R.H. (2012). Triclocarban mediates induction of xenobiotic metabolism through activation of the constitutive androstane receptor and the estrogen receptor alpha. PLoS ONE.

[26] Kajta M., Wójtowicz A.K., Maćkowiak M., Lasoń W. (2009). Aryl hydrocarbon receptor-mediated apoptosis of neuronal cells: A possible interaction with estrogen receptor signaling. Neuroscience.

[27] Tarnow P., Tralau T., Hunecke D., Luch A. (2013). Effects of triclocarban on the transcription of estrogen, androgen and aryl hydrocarbon receptor responsive genes in human breast cancer cells. Toxicol. Vitr..

[28] Xu J., Qian Q., Xia M., Wang X., Wang H. (2021). Trichlorocarban induces developmental and immune toxicity to zebrafish (Danio rerio) by targeting TLR4/MyD88/NF-κB signaling pathway. Environ. Pollut..

[29] Kajta M., Wnuk A., Rzemieniec J., Litwa E., Lason W., Zelek-Molik A., Nalepa I., Rogóż Z., Grochowalski A., Wojtowicz A.K. (2017). Depressive-like effect of prenatal exposure to DDT involves global DNA hypomethylation and impairment of GPER1/ESR1 protein levels but not ESR2 and AHR/ARNT signaling. J. Steroid. Biochem. Mol. Biol..

[30] Schebb N.H., Flores I., Kurobe T., Franze B., Ranganathan A., Hammock B.D., The S.J. (2011). Bioconcentration, metabolism and excretion of triclocarban in larval Qurt medaka (*Oryzias latipes*). Aquat. Toxicol..

[31] Yin J., Wei L., Shi Y., Zhang J., Wu Q., Shao B. (2016). Chinese population exposure to triclosan and triclocarban as measured via human urine and nails. Environ. Geochem. Health.

[32] Geer L.A., Pycke B.F.G., Waxenbaum J., Sherer D.M., Abulafia O., Halden R.U. (2017). Association of birth outcomes with fetal exposure to parabens, triclosan and triclocarban in an immigrant population in Brooklyn, New York. J. Hazard Mater..

[33] Schebb N.H., Inceoglu B., Ahn K.C., Morisseau C., Gee S.J., Hammock B.D. (2011). Investigation of human exposure to triclocarban after showering and preliminary evaluation of its biological effects. Environ. Sci. Technol..

[34] Wnuk A., Rzemieniec J., Staroń J., Litwa E., Lasoń W., Bojarski A., Kajta M. (2019). Prenatal Exposure to Benzophenone-3 Impairs Autophagy, Disrupts RXRs/PPARγ Signaling, and Alters Epigenetic and Post-Translational Statuses in Brain Neurons. Mol. Neurobiol..

[35] Rzemieniec J., Bratek E., Wnuk A., Przepiórska K., Salińska E., Kajta M. (2020). Neuroprotective effect of *3,3′*-Diindolylmethane against perinatal asphyxia involves inhibition of the AhR and NMDA signaling and hypermethylation of specific genes. Apoptosis.

[36] Wnuk A., Rzemieniec J., Przepiórska K., Wesołowska J., Wójtowicz A.K., Kajta M. (2020). Autophagy-related neurotoxicity is mediated via AHR and CAR in mouse neurons exposed to DDE. Sci. Total Environ..

[37] Xie F., Xiao P., Chen D., Xu L., Zhang B. (2012). miRDeepFinder: A miRNA analysis tool for deep sequencing of plant small RNAs. Plant Mol. Biol..

[38] Wnuk A., Rzemieniec J., Litwa E., Lasoń W., Kajta M. (2018). Prenatal exposure to benzophenone-3 (BP-3) induces apoptosis, disrupts estrogen receptor expression and alters the epigenetic status of mouse neurons. J. Steroid Biochem. Mol. Biol..

[39] Eads C.A., Danenberg K.D., Kawakami K., Saltz L.B., Blake C., Shibata D., Danenberg P.V., Laird P.W. (2000). MethyLight: A high-throughput assay to measure DNA methylation. Nucleic Acids Res..

[40] Wnuk A., Rzemieniec J., Lasoń W., Krzeptowski W., Kajta M. (2018). Apoptosis Induced by the UV Filter Benzophenone-3 in Mouse Neuronal Cells Is Mediated *via* Attenuation of Erα/Pparγ and Stimulation of Erβ/Gpr30 Signaling. Mol. Neurobiol..

[41] Rzemieniec J., Wnuk A., Lasoń W., Bilecki W., Kajta M. (2019). The neuroprotective action of *3,3′*-diindolylmethane against ischemia involves an inhibition of apoptosis and autophagy that depends on HDAC and AhR/CYP1A1 but not ERα/CYP19A1 signaling. Apoptosis.

[42] Wnuk A., Przepiórska K., Rzemieniec J., Pietrzak B., Kajta M. (2020). Selective Targeting of Non-nuclear Estrogen Receptors with PaPE-1 as a New Treatment Strategy for Alzheimer’s Disease. Neurotox. Res..

[43] Nakagawa S., Deli M.A., Nakao S., Honda M., Hayashi K., Nakaoke R., Kataoka Y., Niwa M. (2007). Pericytes from brain microvessels strengthen the barrier integrity in primary cultures of rat brain endothelial cells. Cell. Mol. Neurobiol..

[44] Nakagawa S., Deli M.A., Kawaguchi H., Shimizudani T., Shimono T., Kittel A., Tanaka K., Niwa M. (2009). A new blood-brain barrier model using primary rat brain endothelial cells, pericytes and astrocytes. Neurochem. Int..

[45] Ge S.X., Jung D., Yao R. (2020). ShinyGO: A graphical gene-set enrichment tool for animals and plants. Bioinformatics.

[46] Raudvere U., Kolberg L., Kuzmin I., Arak T., Adler P., Peterson H., Vilo J. (2019). g:Profiler: A web server for functional enrichment analysis and conversions of gene lists (2019 update). Nucleic Acids Res..

