# Supplementary material for: Prenatal Exposure to Triclocarban Impairs ESR1 Signaling and Disrupts Epigenetic Status in Sex-Specific Ways as Well as Dysregulates the Expression of Neurogenesis- and Neurotransmitter-Related Genes in the Postnatal Mouse Brain
Source: Int J Mol Sci. 2021 Dec 4;22(23):13121. doi: 10.3390/ijms222313121 (PMC8658534; doi:10.3390/ijms222313121)
Supplement: Supplementary file 1 [file ijms-22-13121-s001.zip › ijms-1448680-supplementary.pdf]

Supplementary material 1 – S1

Table S1. Prenatal exposure to triclocarban dysregulated the expression of neurogenesis- and neurotransmitter-related genes in the prefrontal cortices of one-month-old male (part a) and female (part b) mice that as determined by microarray analyses.

a. Males

Gene ontology	Set of genes
**Upregulated genes**	
neurogenesis-related genes	*Artn, Bmp2, Nog, Notch2, Pou4f1* and *Sox3*
neurotransmitter-related genes	*Adra1a, Adra2a, Adrb2, Adrb3, Avpr1b, Brs3, Chrm1, Chrm4, Chrm5, Chrna3, Chrna4, Chrna7, Drd5, Gabbr1, Gabbr2, Gabra1, Gabra6, Gabrb1, Gabrd, Gabrg3, Gabrq, Gabrr1, Gria1, Gria2, Gria3, Grik2, Grin1, Grm3, Grm6, Grm7, Grm8, Grpr, Hcrtr2, Hrh1, Npy2r, Npy5r, Tacr1,* and *Tacr2*
**Downregulated genes**	
neurogenesis-related genes	*Adora1, Alk, Apbb1, Ascl1, Bdnf, Bmp4, Bmp8b, Chrm2, Dcx, Drd2, Dvl3, Erbb2, Gdnf, Gpi1, Grin1, Hes1, Hey1, Mdk, Map2, Ndn, Ndp, Nrg1, Ntf3, Ntn1, Olig2, Pard3, Pax6, Rtn4, Slit2, Stat3, Th* and *Tnr*
neurotransmitter-related genes	*Adra1d, Avpr1a, Cckbr, Chrna5, Chrna6, Chrne, Drd2, Gabra2, Gabra5, Gabre, Gcgr, Grm5, Htr2a, Htr3a, Htr4* and *Sstr4*

b. Females

Gene ontology	Set of genes
**Upregulated genes**	
neurogenesis-related genes	*Bdnf, Chrm2, Efnb1, Egf, Hes1, Neurog1, Notch2, Nrg1, Nrp1, Ntn1, Pafah1b1, Pax5, Pou3f3, S100b,* and *Shh*
neurotransmitter-related genes	*Adra1d, Chrne, Drd2, Gabra5, Htr2c, Htr4, Oxtr, Prokr2, Sstr4, Tacr3,* and *Tspo*
**Downregulated genes**	
neurogenesis-related genes	*Adora2a, Ascl1, Bmp2, Cdk5rap2, Cxcl1, Dll1, Drd2, Fgf2, Gdnf, Il3, Neurod1, Neurog2, Nog, Notch1, Nr2e3, Ntf3, Olig2, Pax3, Pou4f1, Sox2, Sox3,* and *Th*
neurotransmitter-related genes	*Adra1a, Adra2a, Adrb2, Adrb3, Avpr1a, Brs3, Cckbr, Chrm1, Chrm4, Chrm5, Chrna3, Chrna4, Chrna5, Chrna7, Cnr1, Drd5, Gabbr1, Gabra1, Gabra4, Gabra6, Gabrb1, Gabrd, Gabrg3, Gria1, Gria2, Grik2, Grin1, Grin2a, Grm3, Grm5, Grm6, Grm7, Grm8, Hcrtr2, Hrh1, Htr2a, Tacr1* and *Tacr2*

Supplementary material 1 – S1

Neurogenesis-related genes

**Symbol**	**Description**
Adra1a	Adrenergic, alpha-1A-, receptor
Adra1d	Adrenergic, alpha-1D-, receptor
Adra2a	Adrenergic, alpha-2A-, receptor
Adrb2	Adrenergic, beta-2-, receptor, surface
Adrb3	Adrenergic, beta-3-, receptor
Avpr1a	Arginine vasopressin receptor 1A
Avpr1b	Arginine vasopressin receptor 1B
Brs3	Bombesin-like receptor 3
Cckbr	Cholecystokinin B receptor
Chrm1	Cholinergic receptor, muscarinic 1
Chrm4	Cholinergic receptor, muscarinic 4
Chrm5	Cholinergic receptor, muscarinic 5
Chrna3	Cholinergic receptor, nicotinic, alpha 3
Chrna4	Cholinergic receptor, nicotinic, alpha 4
Chrna5	Cholinergic receptor, nicotinic, alpha 5
Chrna6	Cholinergic receptor, nicotinic, alpha 6
Chrna7	Cholinergic receptor, nicotinic, alpha 7
Chrne	Cholinergic receptor, nicotinic, epsilon
Cnr1	Cannabinoid receptor 1 (brain)
Drd1	Dopamine receptor D1
Drd2	Dopamine receptor D2
Drd5	Dopamine receptor D5
Gabbr1	Gamma-aminobutyric acid (GABA) B receptor, 1
Gabbr2	Gamma-aminobutyric acid (GABA) B receptor, 2
Gabra1	Gamma-aminobutyric acid (GABA) A receptor, alpha 1
Gabra2	Gamma-aminobutyric acid (GABA) A receptor, alpha 2
Gabra4	Gamma-aminobutyric acid (GABA) A receptor, alpha 4
Gabra5	Gamma-aminobutyric acid (GABA) A receptor, alpha 5
Gabra6	Gamma-aminobutyric acid (GABA) A receptor, alpha 6
Gabrb1	Gamma-aminobutyric acid (GABA) A receptor, beta 1
Gabrb3	Gamma-aminobutyric acid (GABA) A receptor, beta 3
Gabrd	Gamma-aminobutyric acid (GABA) A receptor, delta
Gabre	Gamma-aminobutyric acid (GABA) A receptor, epsilon
Gabrg1	Gamma-aminobutyric acid (GABA) A receptor, gamma 1
Gabrg2	Gamma-aminobutyric acid (GABA) A receptor, gamma 2
Gabrg3	Gamma-aminobutyric acid (GABA) A receptor, gamma 3
Gabrq	Gamma-aminobutyric acid (GABA) receptor, theta
Gabrr1	Gamma-aminobutyric acid (GABA) receptor, rho 1
Gabrr2	Gamma-aminobutyric acid (GABA) receptor, rho 2
Gcgr	Glucagon receptor
Gria1	Glutamate receptor, ionotropic, AMPA 1
Gria2	Glutamate receptor, ionotropic, AMPA 2
Gria3	Glutamate receptor, ionotrophic, AMPA 3
Grik1	Glutamate receptor, ionotropic, kainate 1
Grik2	Glutamate receptor, ionotropic, kainate 2
Grik4	Glutamate receptor, ionotropic, kainate 4
Grik5	Glutamate receptor, ionotropic, kainate 5
Grin1	Glutamate receptor, ionotropic, N-methyl D-aspartate 1
Grin2a	Glutamate receptor, ionotropic, N-methyl D-aspartate 2A
Grin2b	Glutamate receptor, ionotropic, N-methyl D-aspartate 2B

Supplementary material 1 – S1

Grin2c	Glutamate receptor, ionotropic, N-methyl D-aspartate 2C
Grm1	Glutamate receptor, metabotropic 1
Grm3	Glutamate receptor, metabotropic 3
Grm4	Glutamate receptor, metabotropic 4
Grm5	Glutamate receptor, metabotropic 5
Grm6	Glutamate receptor, metabotropic 6
Grm7	Glutamate receptor, metabotropic 7
Grm8	Glutamate receptor, metabotropic 8
Grpr	Gastrin-releasing peptide receptor
Hcrtr2	Hypocretin (orexin) receptor 2
Hrh1	Histamine receptor H1
Hrh4	Histamine receptor H4
Htr1a	5-hydroxytryptamine (serotonin) receptor 1A
Htr1b	5-hydroxytryptamine (serotonin) receptor 1B
Htr1d	5-hydroxytryptamine (serotonin) receptor 1D
Htr1f	5-hydroxytryptamine (serotonin) receptor 1F
Htr2a	5-hydroxytryptamine (serotonin) receptor 2A
Htr2c	5-hydroxytryptamine (serotonin) receptor 2C
Htr3a	5-hydroxytryptamine (serotonin) receptor 3A
Htr4	5-hydroxytryptamine (serotonin) receptor 4
Htr7	5-hydroxytryptamine (serotonin) receptor 7 (adenylate cyclase-coupled)
Npy2r	Neuropeptide Y receptor Y2
Npy5r	Neuropeptide Y receptor Y5
Ntsr2	Neurotensin receptor 2
Oxtr	Oxytocin receptor
Prokr2	Prokineticin receptor 2
Sctr	Secretin receptor
Sstr1	Somatostatin receptor 1
Sstr2	Somatostatin receptor 2
Sstr4	Somatostatin receptor 4
Tacr1	Tachykinin receptor 1
Tacr2	Tachykinin receptor 2
Tacr3	Tachykinin receptor 3
Tspo	Translocator protein (18kDa)

Neurotransmitter-related genes

**Symbol**	**Description**
Ache	Acetylcholinesterase
Adora1	Adenosine A1 receptor
Adora2a	Adenosine A2a receptor
Alk	Anaplastic lymphoma kinase
Apbb1	Amyloid beta (A4) precursor protein-binding, family B, member 1
Apoe	Apolipoprotein E
App	Amyloid beta (A4) precursor protein
Artn	Artemin
Ascl1	Achaete-scute complex homolog 1 (Drosophila)
Bcl2	B-cell leukemia/lymphoma 2
Bdnf	Brain derived neurotrophic factor
Bmp2	Bone morphogenetic protein 2

Supplementary material 1 – S1

Bmp4	Bone morphogenetic protein 4
Bmp8b	Bone morphogenetic protein 8b
Cdk5r1	Cyclin-dependent kinase 5, regulatory subunit 1 (p35)
Cdk5rap2	CDK5 regulatory subunit associated protein 2
Chrm2	Cholinergic receptor, muscarinic 2, cardiac
Creb1	CAMP responsive element binding protein 1
Cxcl1	Chemokine (C-X-C motif) ligand 1
Dcx	Doublecortin
Dlg4	Discs, large homolog 4 (Drosophila)
Dll1	Delta-like 1 (Drosophila)
Drd2	Dopamine receptor D2
Dvl3	Dishevelled 3, dsh homolog (Drosophila)
Efnb1	Ephrin B1
Egf	Epidermal growth factor
Ep300	E1A binding protein p300
Erbb2	V-erb-b2 erythroblastic leukemia viral oncogene homolog 2, neuro/glioblastoma derived oncogene homolog (avian)
Fgf2	Fibroblast growth factor 2
Flna	Filamin, alpha
Gdnf	Glial cell line derived neurotrophic factor
Gpi1	Glucose phosphate isomerase 1
Grin1	Glutamate receptor, ionotropic, NMDA1 (zeta 1)
Hdac4	Histone deacetylase 4
Hes1	Hairy and enhancer of split 1 (Drosophila)
Hey1	Hairy/enhancer-of-split related with YRPW motif 1
Hey2	Hairy/enhancer-of-split related with YRPW motif 2
Heyl	Hairy/enhancer-of-split related with YRPW motif-like
Il3	Interleukin 3
Mdk	Midkine
Mef2c	Myocyte enhancer factor 2C
Kmt2a	Myeloid/lymphoid or mixed-lineage leukemia 1
Map2	Microtubule-associated protein 2
Ndn	Necdin
Ndp	Norrie disease (pseudoglioma) (human)
Neurod1	Neurogenic differentiation 1
Neurog1	Neurogenin 1
Neurog2	Neurogenin 2
Nf1	Neurofibromatosis 1
Nog	Noggin
Notch1	Notch gene homolog 1 (Drosophila)
Notch2	Notch gene homolog 2 (Drosophila)
Nr2e3	Nuclear receptor subfamily 2, group E, member 3
Nrcam	Neuron-glia-CAM-related cell adhesion molecule
Nrg1	Neuregulin 1
Nrp1	Neuropilin 1

Nrp2	Neuropilin 2
Ntf3	Neurotrophin 3
Ntn1	Netrin 1
Tenm1	Odd Oz/ten-m homolog 1 (Drosophila)
Olig2	Oligodendrocyte transcription factor 2
Pafah1b1	Platelet-activating factor acetylhydrolase, isoform 1b, subunit 1
Pard3	Par-3 (partitioning defective 3) homolog (C. elegans)
Pax3	Paired box gene 3
Pax5	Paired box gene 5
Pax6	Paired box gene 6
Pou3f3	POU domain, class 3, transcription factor 3
Pou4f1	POU domain, class 4, transcription factor 1
Ptn	Pleiotrophin
Rac1	RAS-related C3 botulinum substrate 1
Robo1	Roundabout homolog 1 (Drosophila)
Rtn4	Reticulon 4
S100a6	S100 calcium binding protein A6 (calcyclin)
S100b	S100 protein, beta polypeptide, neural
Shh	Sonic hedgehog
Slit2	Slit homolog 2 (Drosophila)
Sod1	Superoxide dismutase 1, soluble
Sox2	SRY-box containing gene 2
Sox3	SRY-box containing gene 3
Stat3	Signal transducer and activator of transcription 3
Tgfb1	Transforming growth factor, beta 1
Th	Tyrosine hydroxylase
Tnr	Tenascin R
Vegfa	Vascular endothelial growth factor A
Actb	Actin, beta
B2m	Beta-2 microglobulin
Gapdh	Glyceraldehyde-3-phosphate dehydrogenase
Gusb	Glucuronidase, beta
Hsp90ab1	Heat shock protein 90 alpha (cytosolic), class B member 1

Supplementary material 2 – S2

Table S2: Primer sets

Primer sets

Target Gene	Source
Ahr	Thermo Fisher Scientific, USA - Mm01291777_m1
Arnt	Thermo Fisher Scientific, USA - Mm00507836_m1
Atg7	Thermo Fisher Scientific, USA - Mm00512209_m1
B2m	Thermo Fisher Scientific, USA - Mm00437762_m1
Bax	Thermo Fisher Scientific, USA - Mm00432051_m1
Bcl2	Thermo Fisher Scientific, USA - Mm00477631_m1
Becn1	Thermo Fisher Scientific, USA - Mm01265461_m1
Car	Thermo Fisher Scientific, USA - Mm01283978_m1
Cyp19a1	Thermo Fisher Scientific, USA - Mm00484049_m1
Cyp1a1	Thermo Fisher Scientific, USA - Mm00487218_m1
Esr1	Thermo Fisher Scientific, USA - Mm00433149_m1
Esr2	Thermo Fisher Scientific, USA - Mm00599821_m1
Gapdh	Thermo Fisher Scientific, USA - Mm05724508_g1
Gper1	Thermo Fisher Scientific, USA - Mm01194815_m1
Gsk3b	Thermo Fisher Scientific, USA - Mm00444911_m1
Hprt	Thermo Fisher Scientific, USA - Mm03024075_m1
Map1lc3a	Thermo Fisher Scientific, USA - Mm00458724_m1
Map1lc3b	Thermo Fisher Scientific, USA - Mm00782868_sH
Nup62	Thermo Fisher Scientific, USA - Mm01700766_m1

Gene-specific primers

Target Gene	Source
Esr1	Thermo Fisher Scientific, USA<br>M Probe – CAACAAATTAACCGAAACCCGCTAACCTAA<br>U Probe - CAACAAATTAACCAAAACCCACTAACCTAA
Esr2	Thermo Fisher Scientific, USA<br>M Probe - TCCAATAAATCTAATTACACGACGCGAAAA<br>U Probe - TCCAATAAATCTAATTACACAACACAAAAA
Gper1	Thermo Fisher Scientific, USA<br>M Probe – AAAACAAAAACACCCGCCCGATACCTAAAA<br>U Probe - AAAACAAAAACACCCACCCAATACCTAAAA

Supplementary material 3 – S3

Enrichment analyzes – downregulated genes in the prefrontal cortices of one-month-old males that were prenatally exposed to the triclocarban

Curated reactome

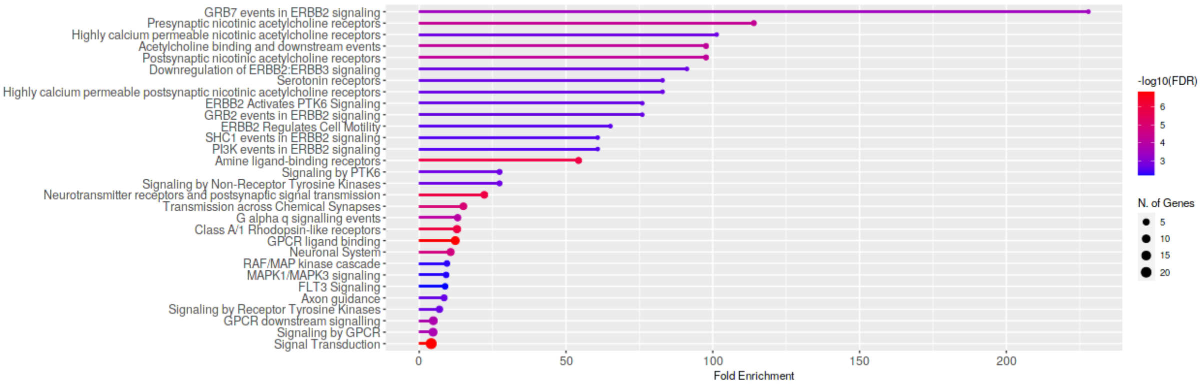

GO Cellular Component

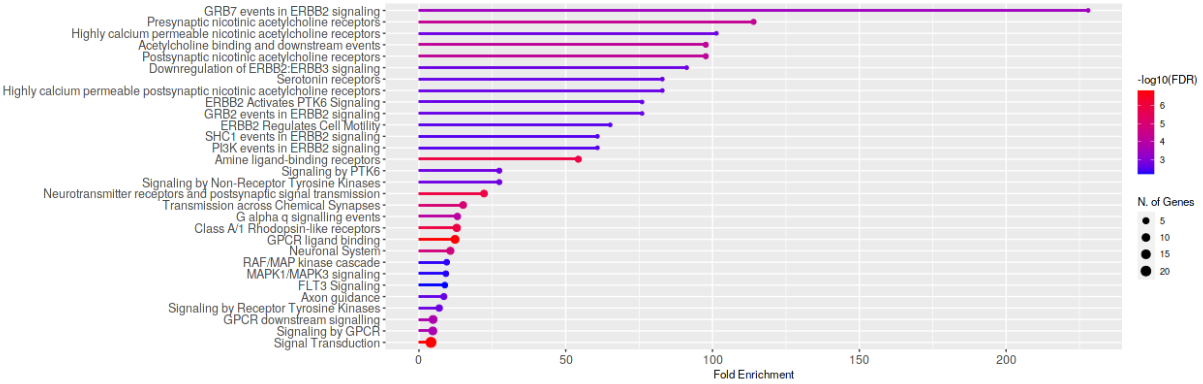

GO Molecular Function

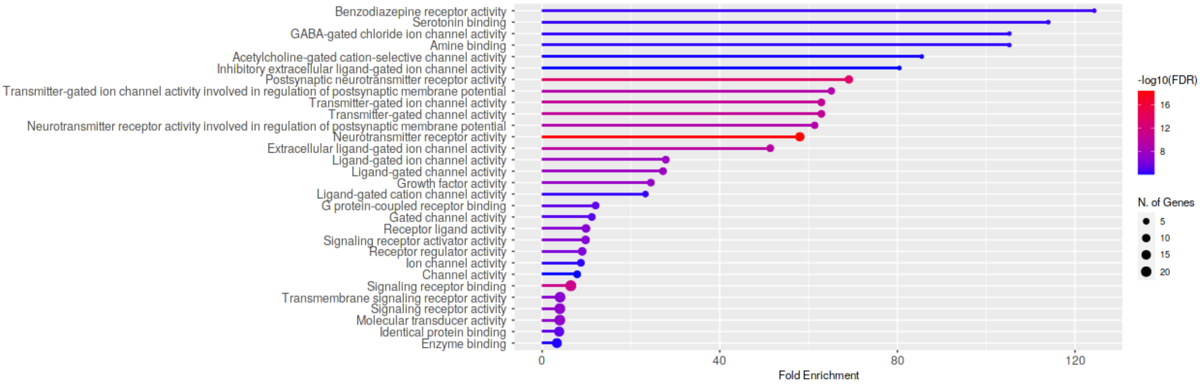

Supplementary material 3 – S3

KEGG

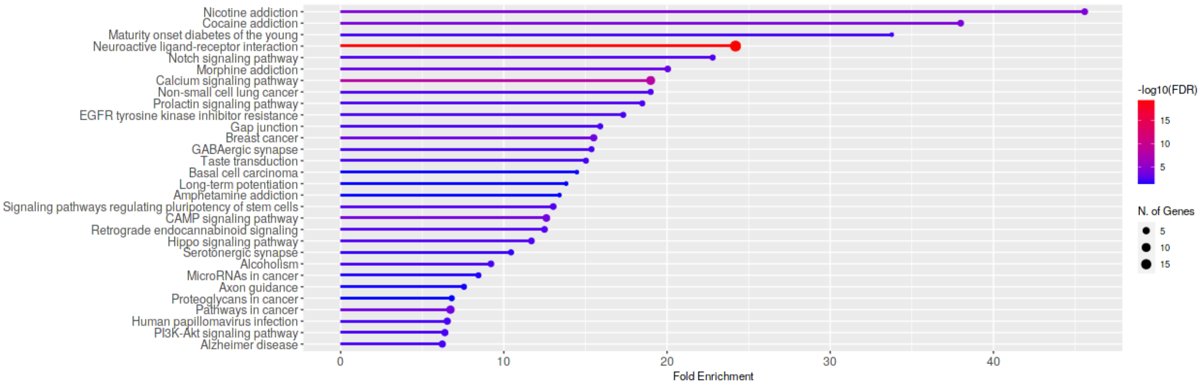

KEGG pathway – Neuroactive ligand-receptor interaction

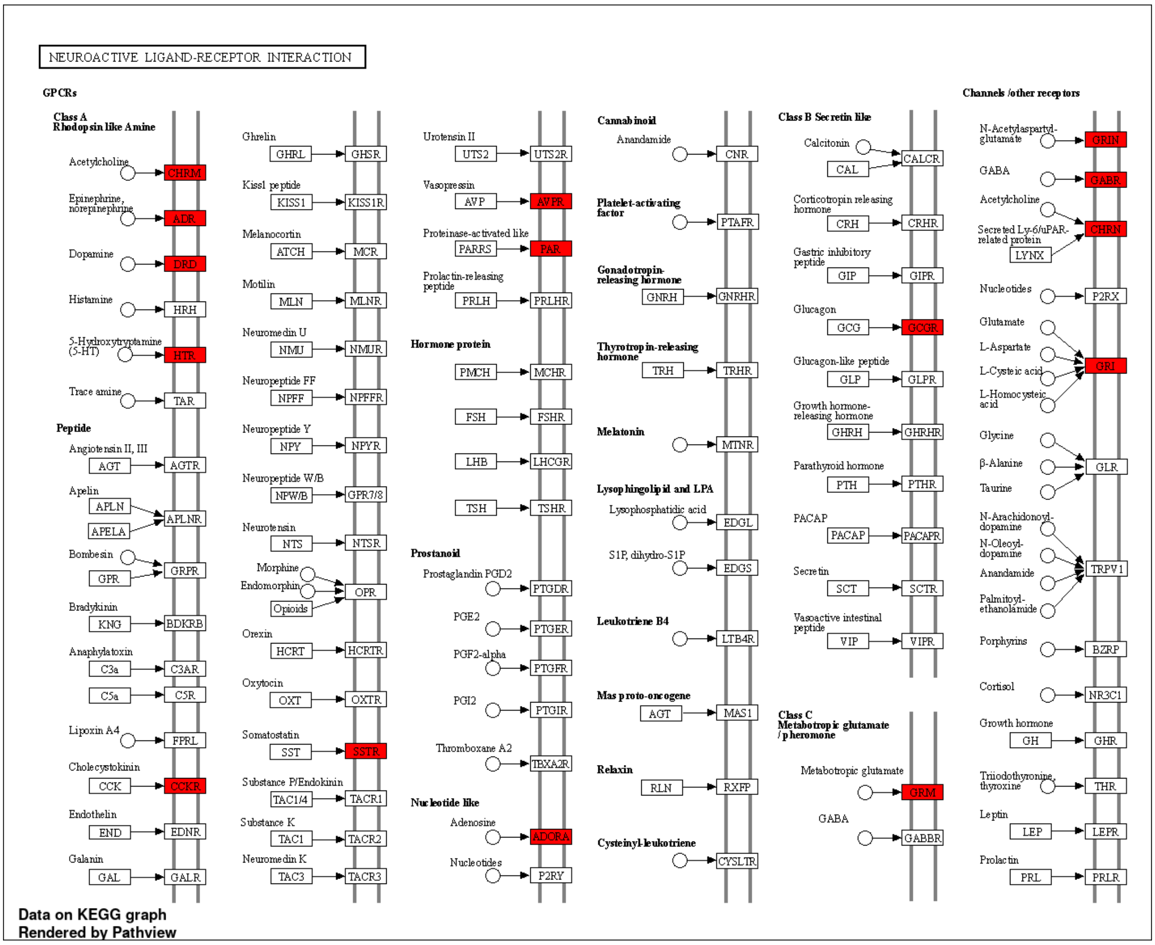

Supplementary material 3 – S3

g:Profiler - g:GOSt

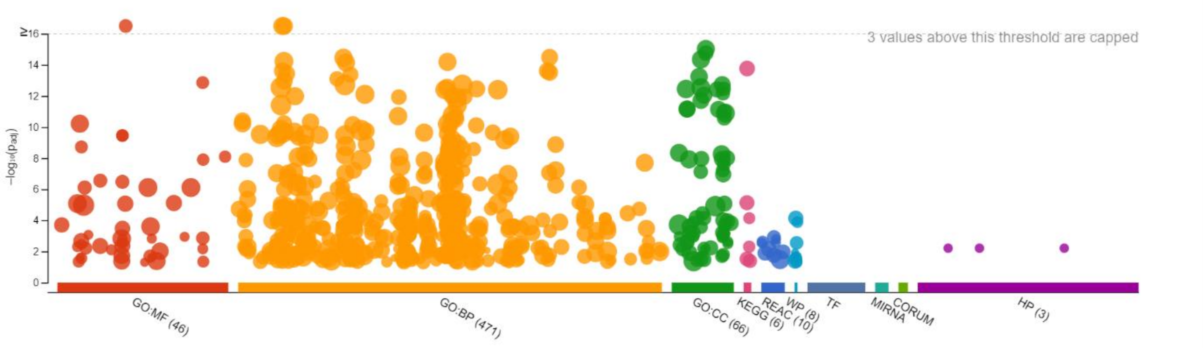

version e104_eg51_p15_3922dba
date 8.11.2021, 14:08:00
organism mmusculus

g:Profiler

GO:MF Term name	Term ID	stats p_{adj}
neurotransmitter receptor activity	GO:0030594	1.082×10^{-18}
postsynaptic neurotransmitter receptor activity	GO:0098960	1.382×10^{-13}
signaling receptor binding	GO:0005102	6.094×10^{-11}
transmitter-gated channel activity	GO:0022835	3.507×10^{-10}
transmitter-gated ion channel activity	GO:0022824	3.507×10^{-10}
extracellular ligand-gated ion channel activity	GO:0005230	1.904×10^{-9}
transmitter-gated ion channel activity involved in regulation...	GO:1904315	8.076×10^{-9}
neurotransmitter receptor activity involved in regulation of ...	GO:0099529	1.251×10^{-8}
ligand-gated ion channel activity	GO:0015276	2.793×10^{-7}
ligand-gated channel activity	GO:0022834	3.347×10^{-7}
molecular transducer activity	GO:0060089	7.767×10^{-7}
signaling receptor activity	GO:0038023	7.767×10^{-7}
growth factor activity	GO:0008083	7.790×10^{-7}
receptor ligand activity	GO:0048018	7.731×10^{-6}
transmembrane signaling receptor activity	GO:0004888	8.348×10^{-6}
signaling receptor activator activity	GO:0030546	8.546×10^{-6}
protein binding	GO:0005515	1.058×10^{-5}
G protein-coupled receptor binding	GO:0001664	1.977×10^{-4}
identical protein binding	GO:0042802	2.508×10^{-4}
gated channel activity	GO:0022836	3.350×10^{-4}
benzodiazepine receptor activity	GO:0008503	8.615×10^{-4}
serotonin binding	GO:0051378	1.147×10^{-3}
ligand-gated cation channel activity	GO:0099094	1.401×10^{-3}
amine binding	GO:0043176	1.488×10^{-3}
GABA-gated chloride ion channel activity	GO:0022851	1.488×10^{-3}
enzyme binding	GO:0019899	1.519×10^{-3}
ion channel activity	GO:0005216	2.129×10^{-3}
acetylcholine-gated cation-selective channel activity	GO:0022848	2.900×10^{-3}
inhibitory extracellular ligand-gated ion channel activity	GO:0005237	3.516×10^{-3}
passive transmembrane transporter activity	GO:0022803	4.493×10^{-3}
channel activity	GO:0015267	4.493×10^{-3}
GABA-A receptor activity	GO:0004890	4.994×10^{-3}
G protein-coupled amine receptor activity	GO:0008227	6.169×10^{-3}
ligand-gated anion channel activity	GO:0099095	6.832×10^{-3}
GABA receptor activity	GO:0016917	7.898×10^{-3}
protein dimerization activity	GO:0046983	9.692×10^{-3}
protein kinase binding	GO:0019901	1.846×10^{-2}
hormone binding	GO:0042562	2.020×10^{-2}
ErbB-3 class receptor binding	GO:0043125	2.381×10^{-2}
nerve growth factor receptor binding	GO:0005163	2.381×10^{-2}
excitatory extracellular ligand-gated ion channel activity	GO:0005231	2.749×10^{-2}
protein-containing complex binding	GO:0044877	4.182×10^{-2}
kinase binding	GO:0019900	4.246×10^{-2}
G protein-coupled serotonin receptor activity	GO:0004993	4.560×10^{-2}
serotonin receptor activity	GO:0099589	4.560×10^{-2}
dopamine binding	GO:0035240	4.986×10^{-2}

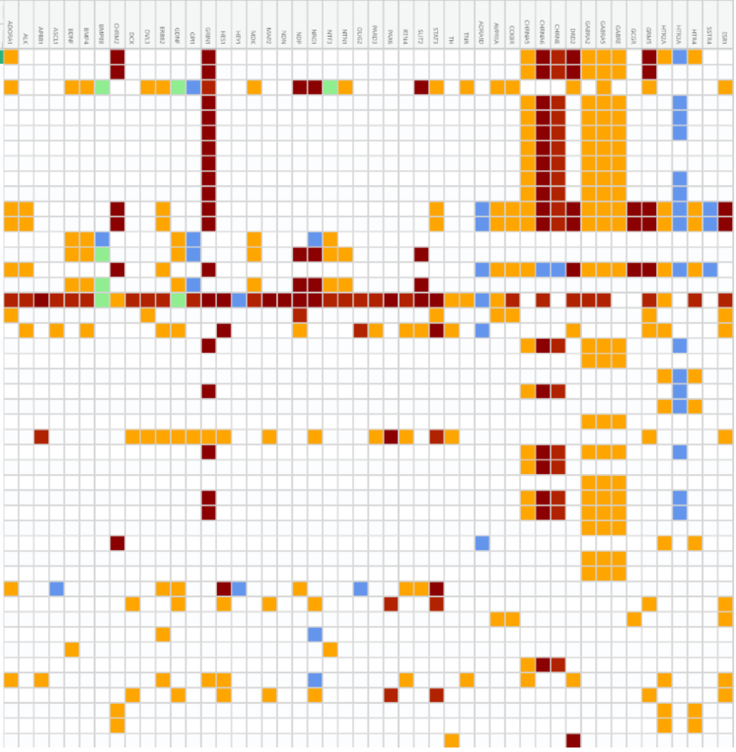

Supplementary material 3 – S3

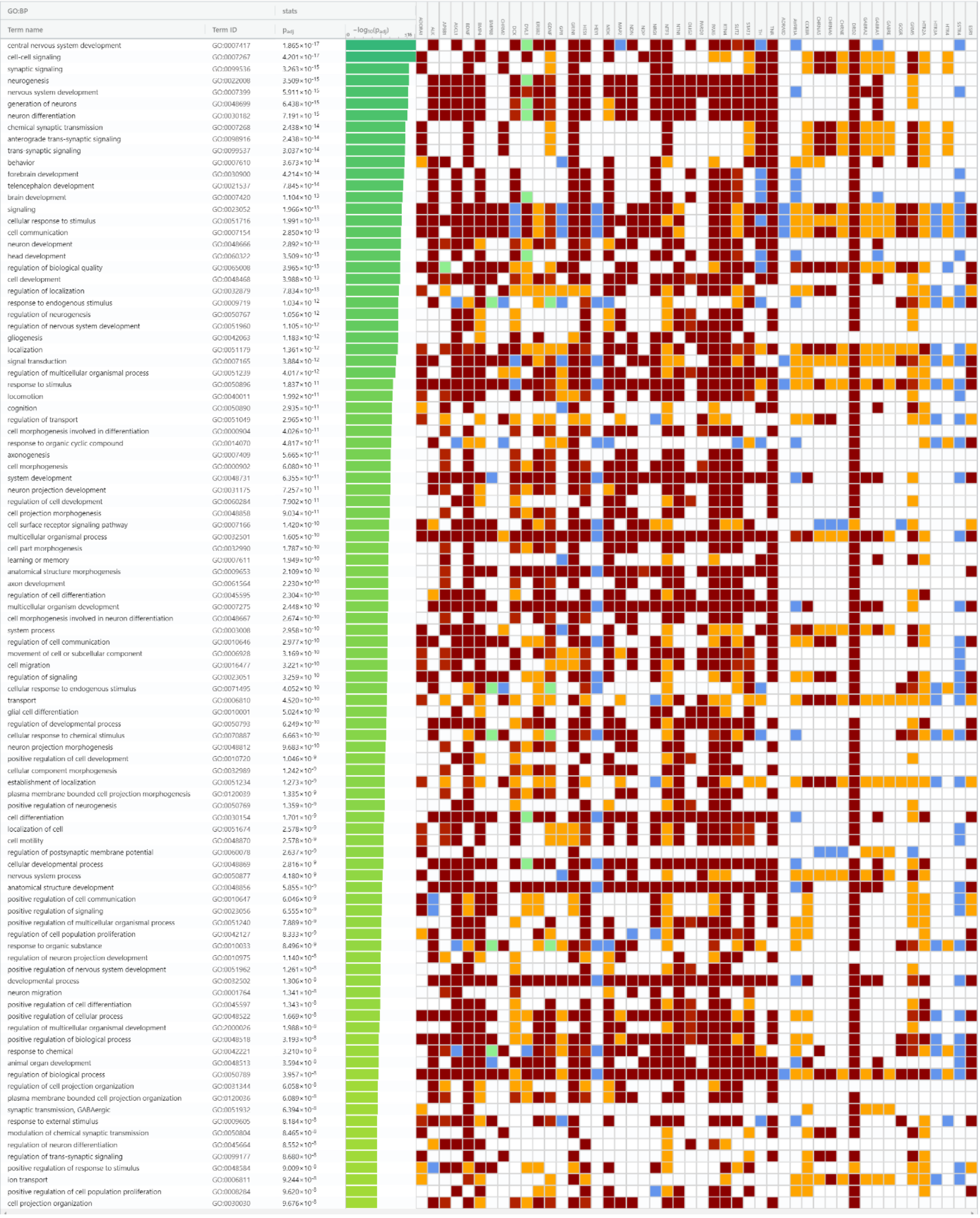

Supplementary material 3 – S3

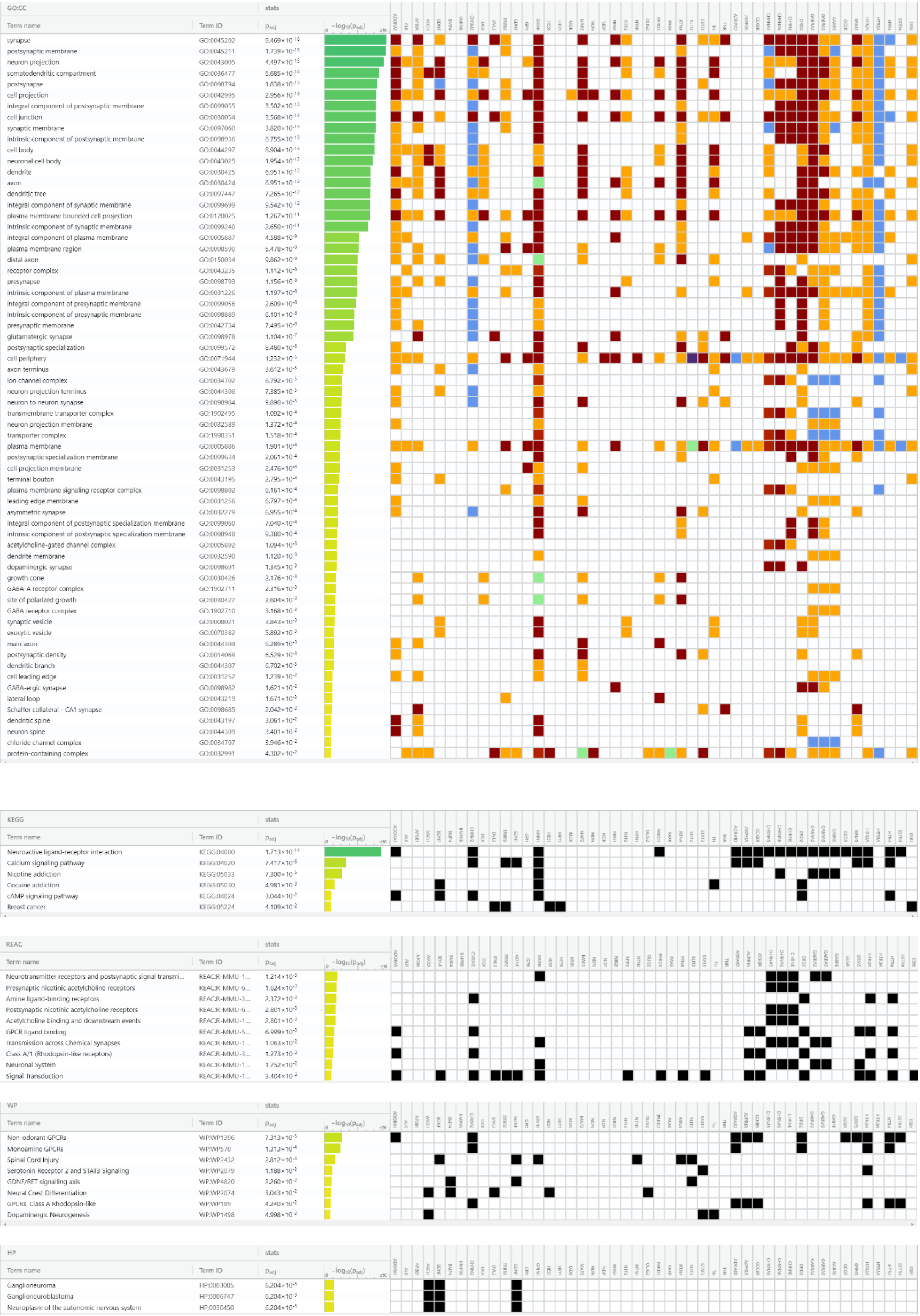

GO:CC

Term name	Term ID	p_{adj}
synapse	GO:0045202	9.469×10^{-16}
postsynaptic membrane	GO:0045211	1.739×10^{-15}
neuron projection	GO:0043005	4.497×10^{-15}
somatodendritic compartment	GO:0036477	5.685×10^{-14}
postsynapse	GO:0098794	1.838×10^{-13}
cell projection	GO:0042995	2.956×10^{-13}
integral component of postsynaptic membrane	GO:0099055	3.502×10^{-13}
cell junction	GO:0030054	3.568×10^{-13}
synaptic membrane	GO:0097060	3.820×10^{-13}
intrinsic component of postsynaptic membrane	GO:0098936	6.755×10^{-13}
cell body	GO:0044297	8.904×10^{-13}
neuronal cell body	GO:0043025	1.954×10^{-12}
dendrite	GO:0030425	6.951×10^{-12}
axon	GO:0030424	6.951×10^{-12}
dendritic tree	GO:0097447	7.265×10^{-12}
integral component of synaptic membrane	GO:0099699	9.542×10^{-12}
plasma membrane bounded cell projection	GO:0120025	1.267×10^{-11}
intrinsic component of synaptic membrane	GO:0099240	2.650×10^{-11}
integral component of plasma membrane	GO:0005887	4.588×10^{-9}
plasma membrane region	GO:0098590	5.478×10^{-9}
distal axon	GO:0150034	9.862×10^{-9}
receptor complex	GO:0043235	1.112×10^{-8}
presynapse	GO:0098793	1.156×10^{-8}
intrinsic component of plasma membrane	GO:0031226	1.197×10^{-8}
integral component of presynaptic membrane	GO:0099056	2.609×10^{-8}
intrinsic component of presynaptic membrane	GO:0098889	6.101×10^{-8}
presynaptic membrane	GO:0042734	7.495×10^{-8}
glutamatergic synapse	GO:0098978	1.104×10^{-7}
postsynaptic specialization	GO:0099572	8.480×10^{-6}
cell periphery	GO:0071944	1.232×10^{-5}
axon terminus	GO:0043679	3.612×10^{-5}
ion channel complex	GO:0034702	6.792×10^{-5}
neuron projection terminus	GO:0044306	7.385×10^{-5}
neuron to neuron synapse	GO:0098984	9.890×10^{-5}
transmembrane transporter complex	GO:1902495	1.092×10^{-4}
neuron projection membrane	GO:0032589	1.372×10^{-4}
transporter complex	GO:1990351	1.518×10^{-4}
plasma membrane	GO:0005886	1.901×10^{-4}
postsynaptic specialization membrane	GO:0099634	2.061×10^{-4}
cell projection membrane	GO:0031253	2.476×10^{-4}
terminal bouton	GO:0043195	2.795×10^{-4}
plasma membrane signaling receptor complex	GO:0098802	6.161×10^{-4}
leading edge membrane	GO:0031256	6.797×10^{-4}
asymmetric synapse	GO:0032279	6.955×10^{-4}
integral component of postsynaptic specialization membrane	GO:0099060	7.040×10^{-4}
intrinsic component of postsynaptic specialization membrane	GO:0098948	9.380×10^{-4}
acetylcholine-gated channel complex	GO:0005892	1.094×10^{-3}
dendrite membrane	GO:0032590	1.120×10^{-3}
dopaminergic synapse	GO:0098691	1.345×10^{-3}
growth cone	GO:0030426	2.176×10^{-3}
GABA-A receptor complex	GO:1902711	2.316×10^{-3}
site of polarized growth	GO:0030427	2.604×10^{-3}
GABA receptor complex	GO:1902710	3.168×10^{-3}
synaptic vesicle	GO:0008021	3.843×10^{-3}
exocytic vesicle	GO:0070382	5.892×10^{-3}
main axon	GO:0044304	6.289×10^{-3}
postsynaptic density	GO:0014069	6.529×10^{-3}
dendritic branch	GO:0044307	6.702×10^{-3}
cell leading edge	GO:0031252	1.239×10^{-2}
GABA-ergic synapse	GO:0098982	1.621×10^{-2}
lateral loop	GO:0043219	1.671×10^{-2}
Schaffer collateral - CA1 synapse	GO:0098685	2.042×10^{-2}
dendritic spine	GO:0043197	3.061×10^{-2}
neuron spine	GO:0044309	3.401×10^{-2}
chloride channel complex	GO:0034707	3.946×10^{-2}
protein-containing complex	GO:0032991	4.302×10^{-2}

KEGG

Term name	Term ID	p_{adj}
Neuroactive ligand-receptor interaction	KEGG:04080	1.713×10^{-14}
Calcium signaling pathway	KEGG:04020	7.417×10^{-6}
Nicotine addiction	KEGG:05033	7.300×10^{-5}
Cocaine addiction	KEGG:05030	4.981×10^{-3}
cAMP signaling pathway	KEGG:04024	3.044×10^{-2}
Breast cancer	KEGG:05224	4.109×10^{-2}

REAC

Term name	Term ID	p_{adj}
Neurotransmitter receptors and postsynaptic signal transmi...	REAC:R-MMU-1...	1.214×10^{-3}
Presynaptic nicotinic acetylcholine receptors	REAC:R-MMU-6...	1.624×10^{-3}
Amine ligand-binding receptors	REAC:R-MMU-3...	2.372×10^{-3}
Postsynaptic nicotinic acetylcholine receptors	REAC:R-MMU-6...	2.801×10^{-3}
Acetylcholine binding and downstream events	REAC:R-MMU-1...	2.801×10^{-3}
GPCR ligand binding	REAC:R-MMU-5...	6.999×10^{-3}
Transmission across Chemical Synapses	REAC:R-MMU-1...	1.063×10^{-2}
Class A/1 (Rhodopsin-like receptors)	REAC:R-MMU-3...	1.273×10^{-2}
Neuronal System	REAC:R-MMU-1...	1.752×10^{-2}
Signal Transduction	REAC:R-MMU-1...	3.404×10^{-2}

WP

Term name	Term ID	p_{adj}
Non-odorant GPCRs	WP:WP1396	7.313×10^{-5}
Monoamine GPCRs	WP:WP570	1.313×10^{-4}
Spinal Cord Injury	WP:WP2432	2.812×10^{-3}
Serotonin Receptor 2 and STAT3 Signaling	WP:WP2079	1.188×10^{-2}
GDNF/RET signalling axis	WP:WP4820	2.260×10^{-2}
Neural Crest Differentiation	WP:WP2074	3.043×10^{-2}
GPCRs, Class A Rhodopsin-like	WP:WP189	4.240×10^{-2}
Dopaminergic Neurogenesis	WP:WP1498	4.998×10^{-2}

HP

Term name	Term ID	p_{adj}
Ganglioneuroma	HP:0003005	6.204×10^{-3}
Ganglioneuroblastoma	HP:0006747	6.204×10^{-3}
Neuroplasm of the autonomic nervous system	HP:0030450	6.204×10^{-3}

Supplementary material 4 – S4

Enrichment analyzes – downregulated genes in the prefrontal cortices of one-month-old females that were prenatally exposed to the triclocarban

Curated reactome

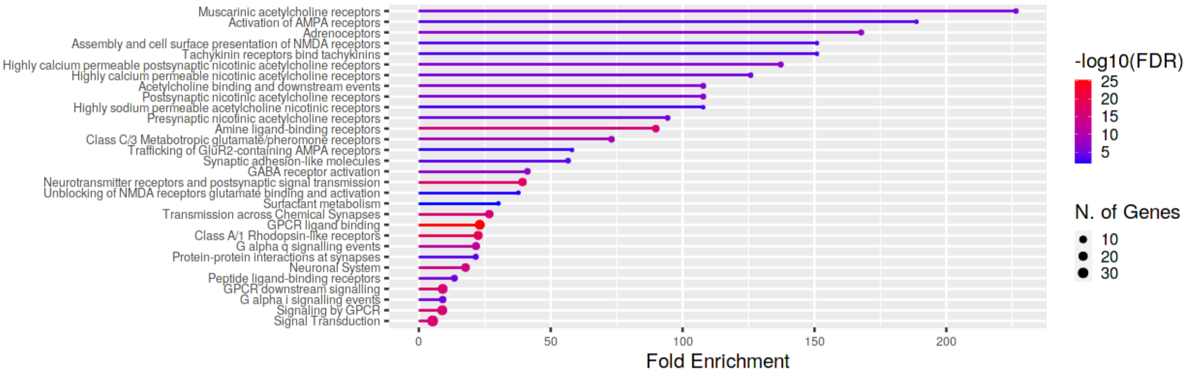

GO Cellular Component

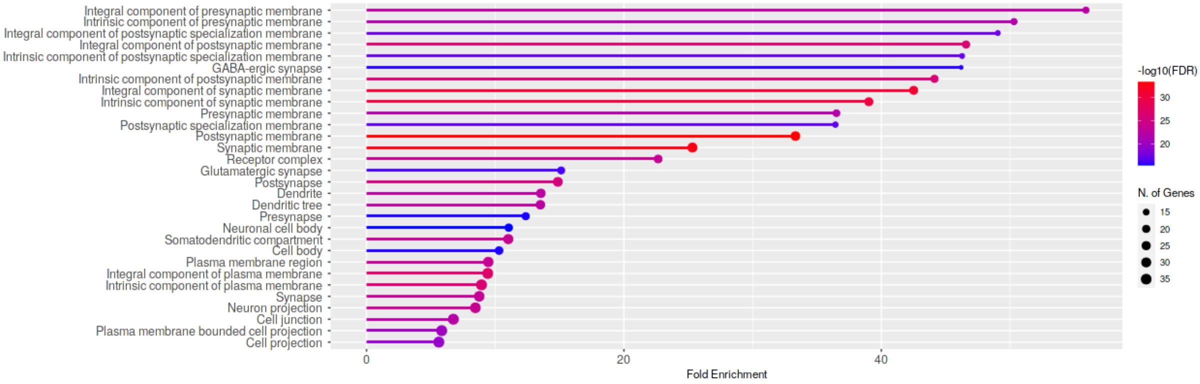

GO Molecular Function

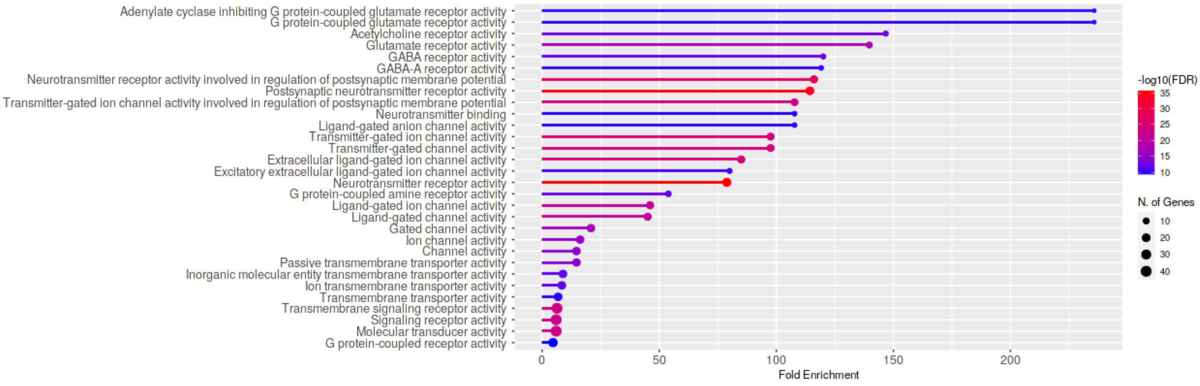

Supplementary material 4 – S4

KEGG

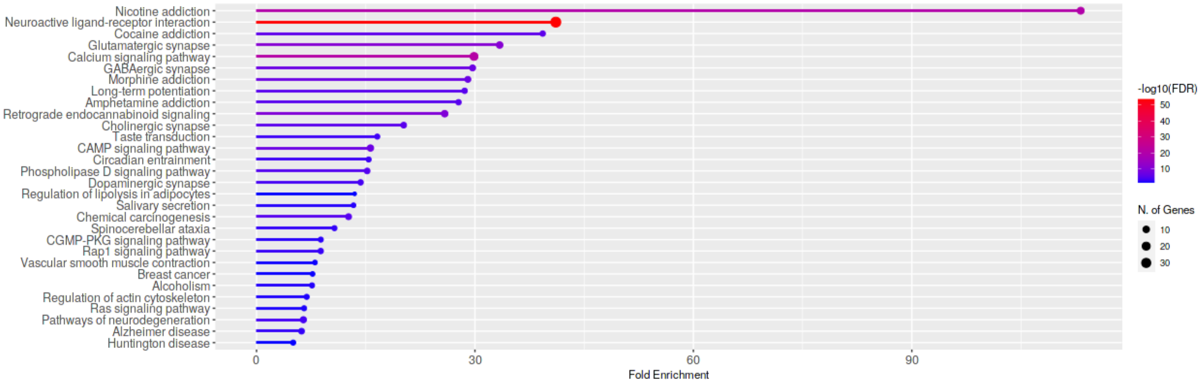

KEGG pathway – Neuroactive ligand-receptor interaction

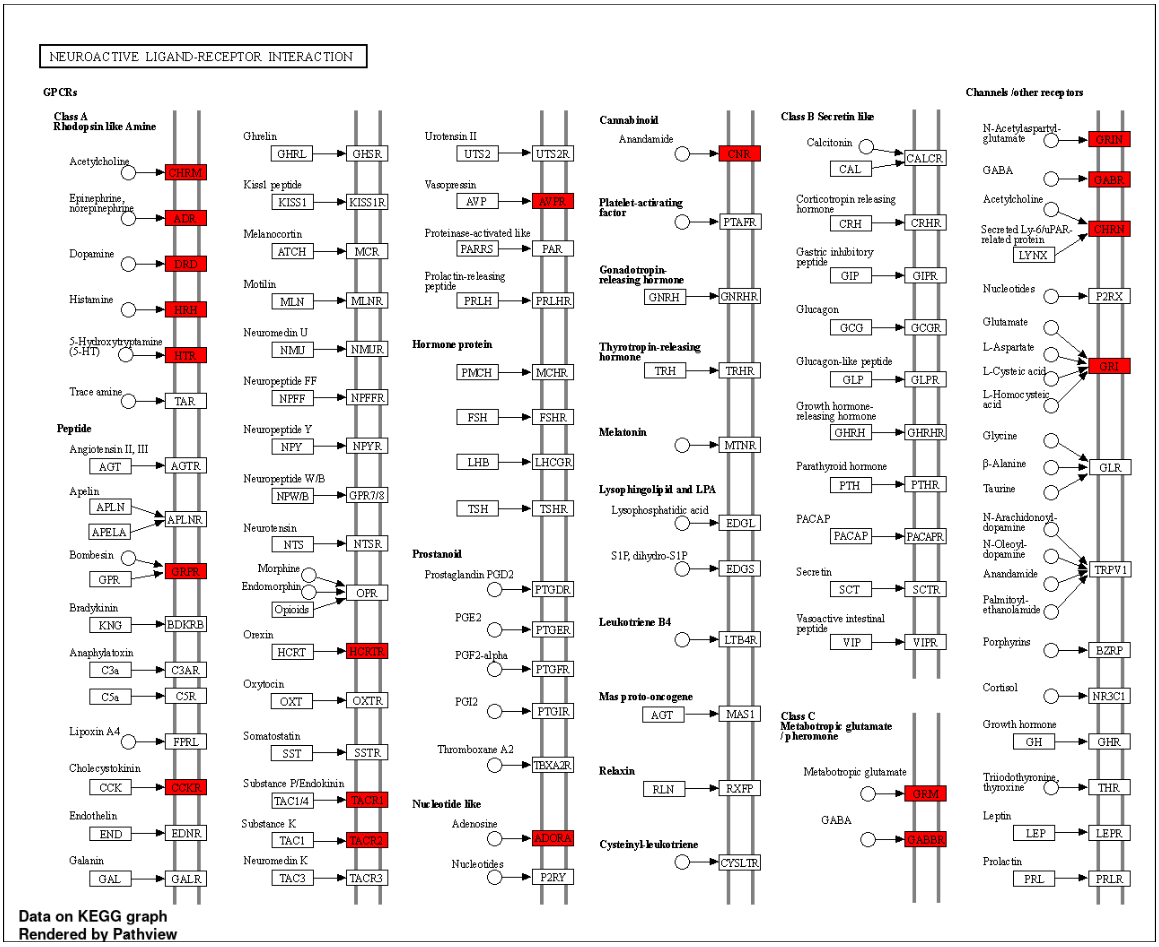

Supplementary material 4 – S4

KEGG pathway – Glutaminergic synapse

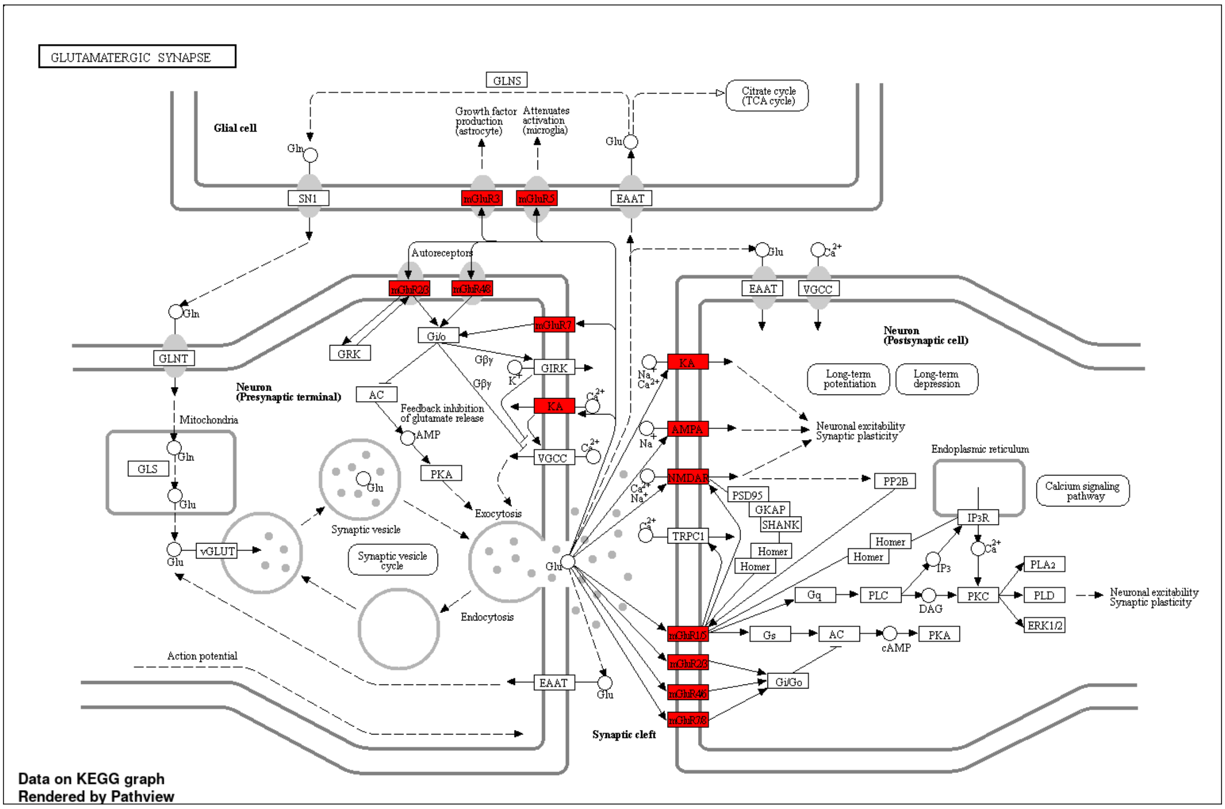

g:Profiler - g:GOSt

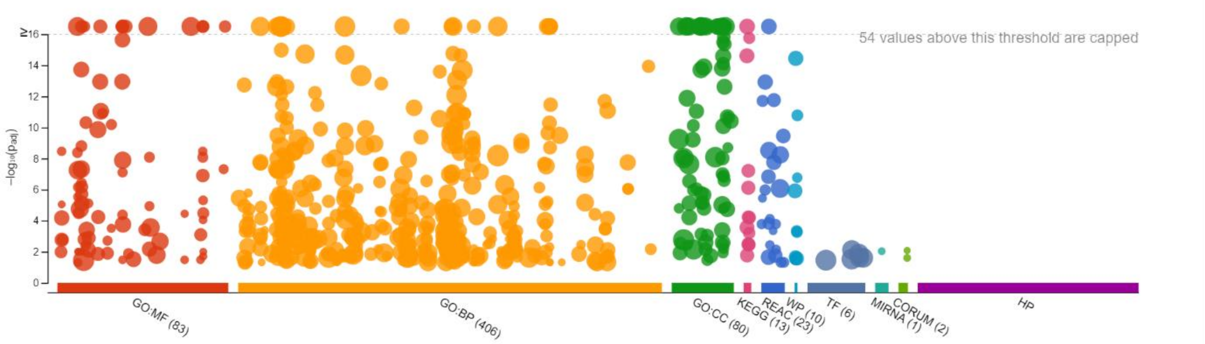

Supplementary material 4 – S4

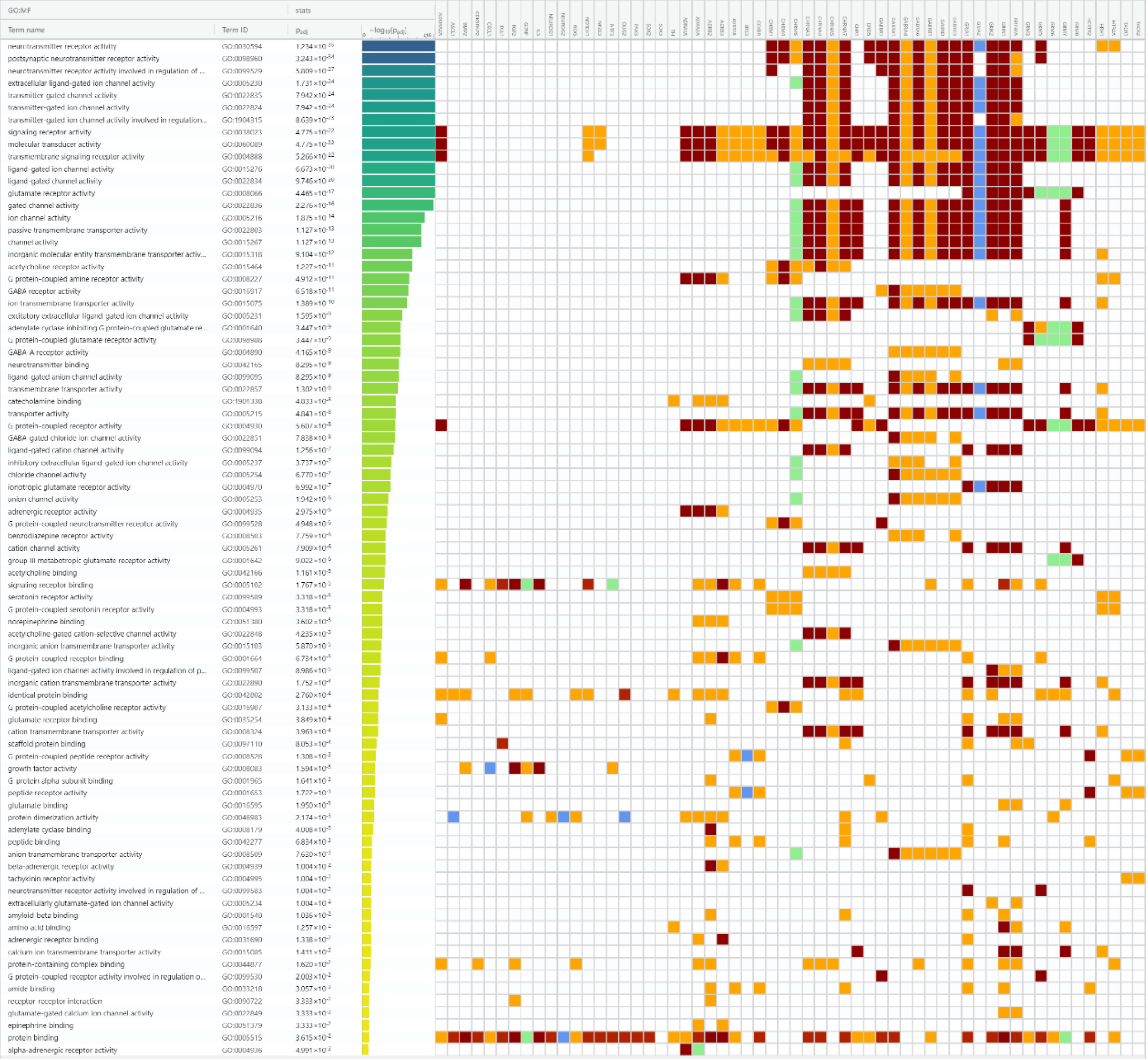

GO:MF		stats
Term name	Term ID	P_{adj}
neurotransmitter receptor activity	GO:0030594	1.234×10^{-35}
postsynaptic neurotransmitter receptor activity	GO:0098960	3.243×10^{-34}
neurotransmitter receptor activity involved in regulation of ...	GO:0099529	5.809×10^{-27}
extracellular ligand-gated ion channel activity	GO:0005230	1.731×10^{-24}
transmitter gated channel activity	GO:0022835	7.942×10^{-24}
transmitter-gated ion channel activity	GO:0022824	7.942×10^{-24}
transmitter-gated ion channel activity involved in regulation...	GO:1904315	8.639×10^{-23}
signaling receptor activity	GO:0038023	4.775×10^{-22}
molecular transducer activity	GO:0060089	4.775×10^{-22}
transmembrane signaling receptor activity	GO:0004888	5.266×10^{-22}
ligand-gated ion channel activity	GO:0015276	6.673×10^{-20}
ligand-gated channel activity	GO:0022834	9.746×10^{-20}
glutamate receptor activity	GO:0008066	4.465×10^{-17}
gated channel activity	GO:0022836	2.276×10^{-16}
ion channel activity	GO:0005216	1.875×10^{-14}
passive transmembrane transporter activity	GO:0022803	1.127×10^{-13}
channel activity	GO:0015267	1.127×10^{-13}
inorganic molecular entity transmembrane transporter activ...	GO:0015318	9.104×10^{-12}
acetylcholine receptor activity	GO:0015464	1.227×10^{-11}
G protein-coupled amine receptor activity	GO:0008227	4.912×10^{-11}
GABA receptor activity	GO:0016917	6.518×10^{-11}
ion transmembrane transporter activity	GO:0015075	1.389×10^{-10}
excitatory extracellular ligand gated ion channel activity	GO:0005231	1.595×10^{-9}
adenylate cyclase inhibiting G protein-coupled glutamate re...	GO:0001640	3.447×10^{-9}
G protein-coupled glutamate receptor activity	GO:0098988	3.447×10^{-9}
GABA-A receptor activity	GO:0004890	4.165×10^{-9}
neurotransmitter binding	GO:0042165	8.295×10^{-9}
ligand gated anion channel activity	GO:0099095	8.295×10^{-9}
transmembrane transporter activity	GO:0022857	1.302×10^{-8}
catecholamine binding	GO:1901338	4.833×10^{-8}
transporter activity	GO:0005215	4.843×10^{-8}
G protein-coupled receptor activity	GO:0004930	5.607×10^{-8}
GABA gated chloride ion channel activity	GO:0022851	7.838×10^{-8}
ligand-gated cation channel activity	GO:0099094	1.256×10^{-7}
inhibitory extracellular ligand-gated ion channel activity	GO:0005237	3.737×10^{-7}
chloride channel activity	GO:0005254	6.770×10^{-7}
ionotropic glutamate receptor activity	GO:0004970	6.992×10^{-7}
anion channel activity	GO:0005253	1.942×10^{-6}
adrenergic receptor activity	GO:0004935	2.975×10^{-6}
G protein-coupled neurotransmitter receptor activity	GO:0099528	4.948×10^{-6}
benzodiazepine receptor activity	GO:0008503	7.759×10^{-6}
cation channel activity	GO:0005261	7.909×10^{-6}
group III metabotropic glutamate receptor activity	GO:0001642	9.022×10^{-6}
acetylcholine binding	GO:0042166	1.161×10^{-5}
signaling receptor binding	GO:0005102	1.767×10^{-5}
serotonin receptor activity	GO:0099589	3.318×10^{-5}
G protein-coupled serotonin receptor activity	GO:0004993	3.318×10^{-5}
norepinephrine binding	GO:0051380	3.602×10^{-5}
acetylcholine-gated cation-selective channel activity	GO:0022848	4.235×10^{-5}
inorganic anion transmembrane transporter activity	GO:0015103	5.870×10^{-5}
G protein coupled receptor binding	GO:0001664	6.734×10^{-5}
ligand-gated ion channel activity involved in regulation of p...	GO:0099507	8.986×10^{-5}
inorganic cation transmembrane transporter activity	GO:0022890	1.752×10^{-4}
identical protein binding	GO:0042802	2.760×10^{-4}
G protein-coupled acetylcholine receptor activity	GO:0016907	3.133×10^{-4}
glutamate receptor binding	GO:0035254	3.849×10^{-4}
cation transmembrane transporter activity	GO:0008324	3.963×10^{-4}
scaffold protein binding	GO:0097110	8.053×10^{-4}
G protein-coupled peptide receptor activity	GO:0008528	1.308×10^{-3}
growth factor activity	GO:0008083	1.594×10^{-3}
G protein alpha subunit binding	GO:0001965	1.641×10^{-3}
peptide receptor activity	GO:0001653	1.722×10^{-3}
glutamate binding	GO:0016595	1.950×10^{-3}
protein dimerization activity	GO:0046983	2.174×10^{-3}
adenylate cyclase binding	GO:0008179	4.008×10^{-3}
peptide binding	GO:0042277	6.834×10^{-3}
anion transmembrane transporter activity	GO:0008509	7.630×10^{-3}
beta-adrenergic receptor activity	GO:0004939	1.004×10^{-2}
tachykinin receptor activity	GO:0004995	1.004×10^{-2}
neurotransmitter receptor activity involved in regulation of ...	GO:0099583	1.004×10^{-2}
extracellularly glutamate-gated ion channel activity	GO:0005234	1.004×10^{-2}
amyloid-beta binding	GO:0001540	1.036×10^{-2}
amino acid binding	GO:0016597	1.257×10^{-2}
adrenergic receptor binding	GO:0031690	1.338×10^{-2}
calcium ion transmembrane transporter activity	GO:0015085	1.411×10^{-2}
protein-containing complex binding	GO:0044877	1.620×10^{-2}
G protein-coupled receptor activity involved in regulation o...	GO:0099530	2.003×10^{-2}
amide binding	GO:0033218	3.057×10^{-2}
receptor receptor interaction	GO:0090722	3.333×10^{-2}
glutamate-gated calcium ion channel activity	GO:0022849	3.333×10^{-2}
epinephrine binding	GO:0051379	3.333×10^{-2}
protein binding	GO:0005515	3.615×10^{-2}
alpha-adrenergic receptor activity	GO:0004936	4.991×10^{-2}

Supplementary material 4 – S4

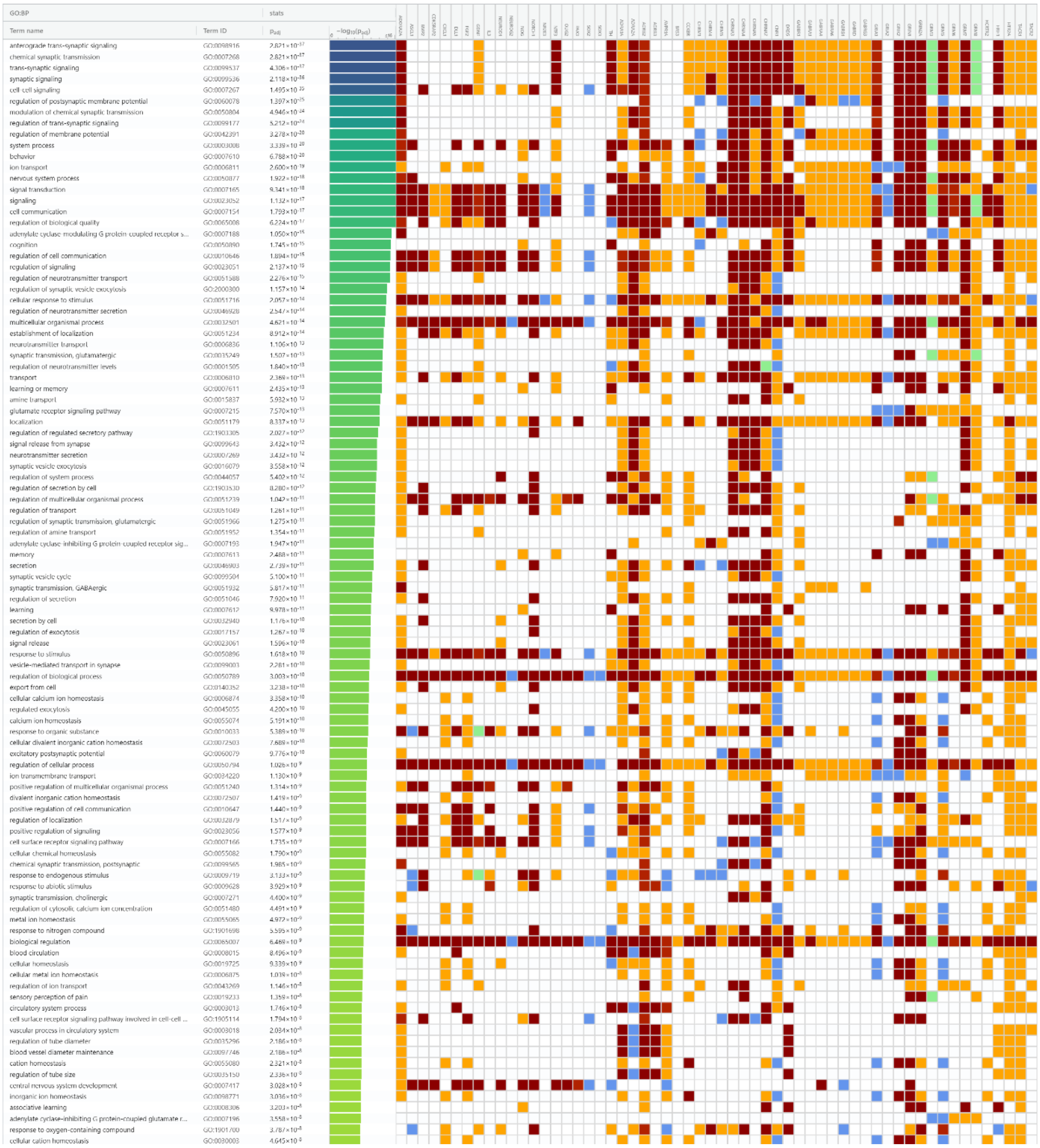

Supplementary material 4 – S4

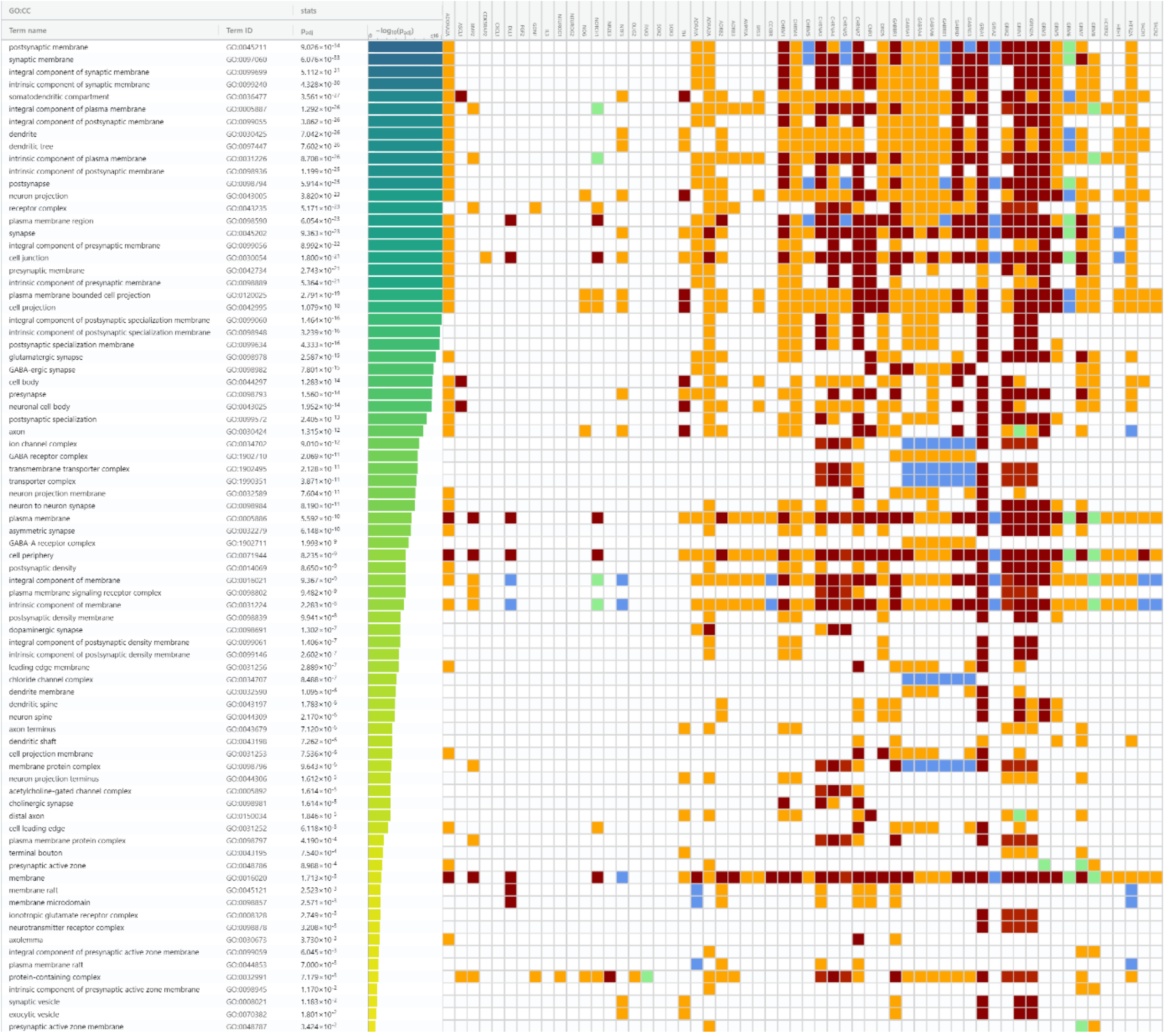

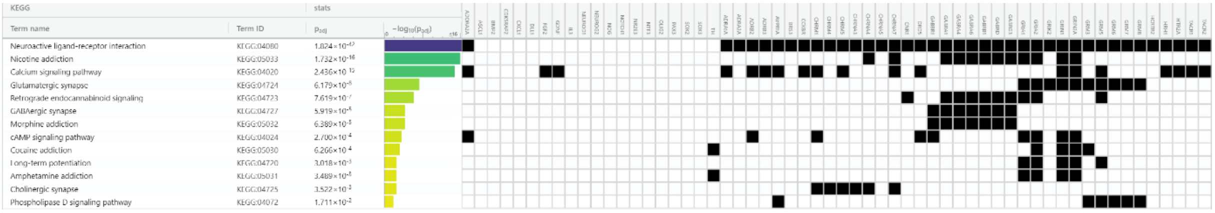

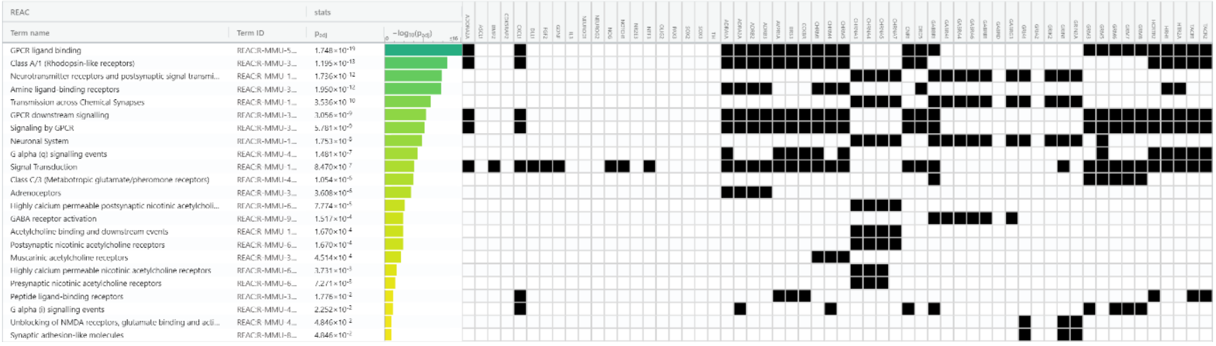

Supplementary material 4 – S4

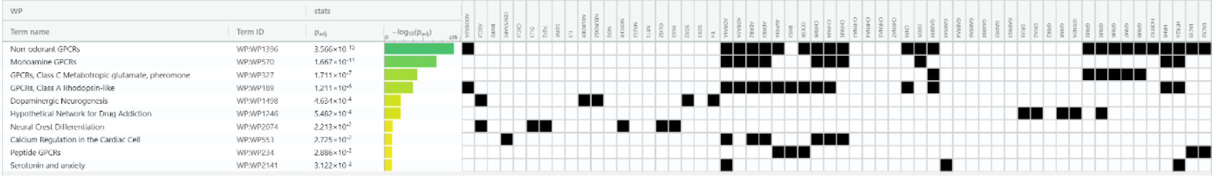

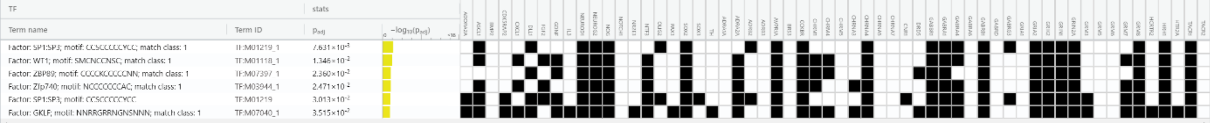

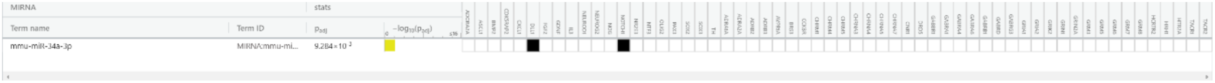

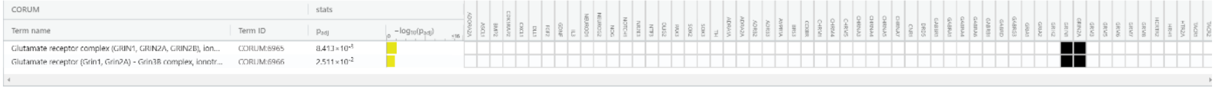

Supplementary material 5 – S5

Enrichment analyzes – upregulated genes in the prefrontal cortices of one-month-old males that were prenatally exposed to the triclocarban

Curated reactome

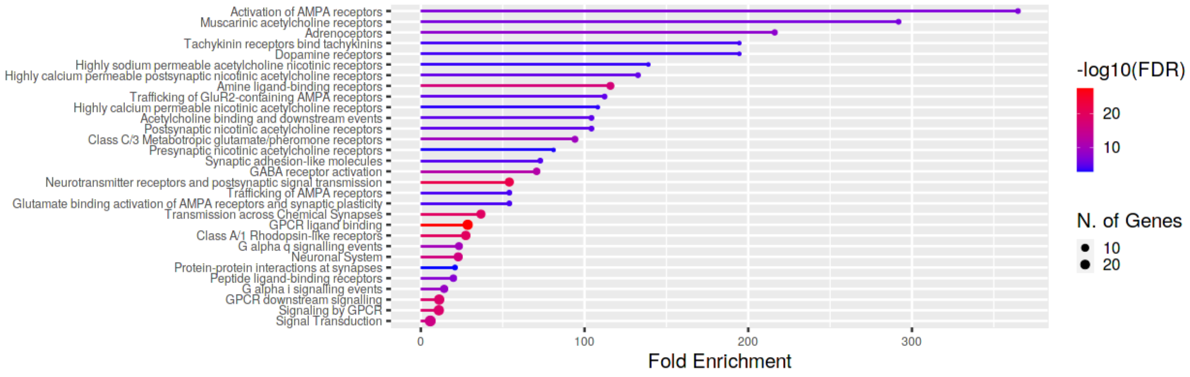

GO Cellular Component

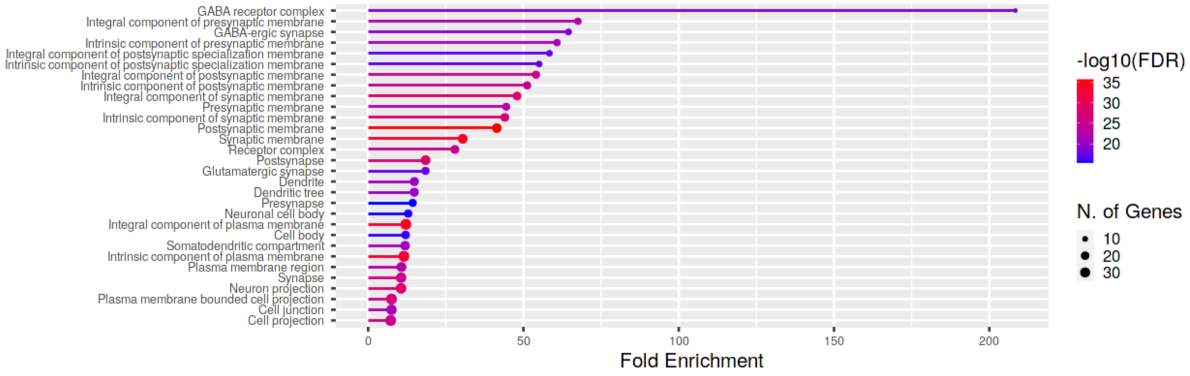

GO Molecular Function

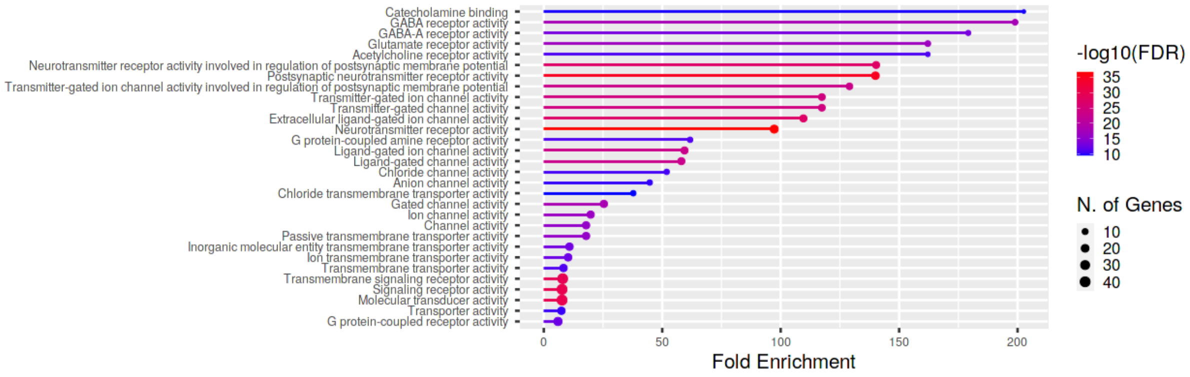

Supplementary material 5 – S5

KEGG

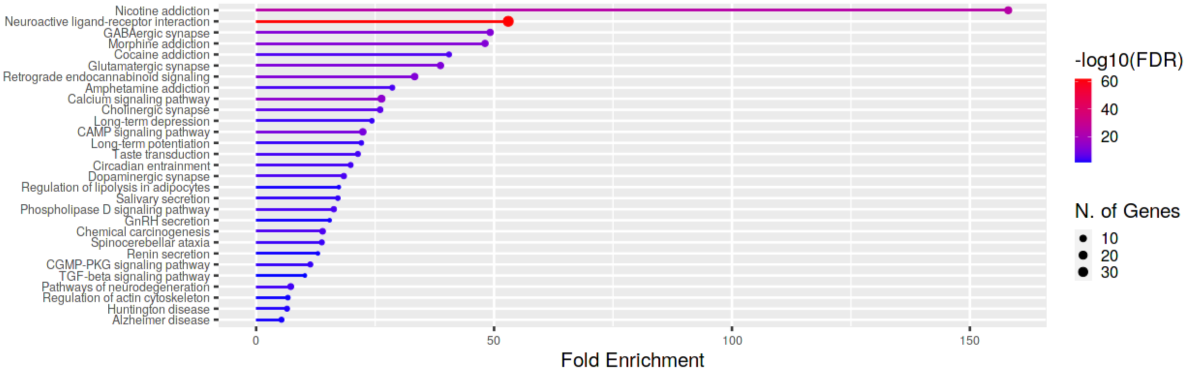

KEGG pathway – Neuroactive ligand-receptor interaction

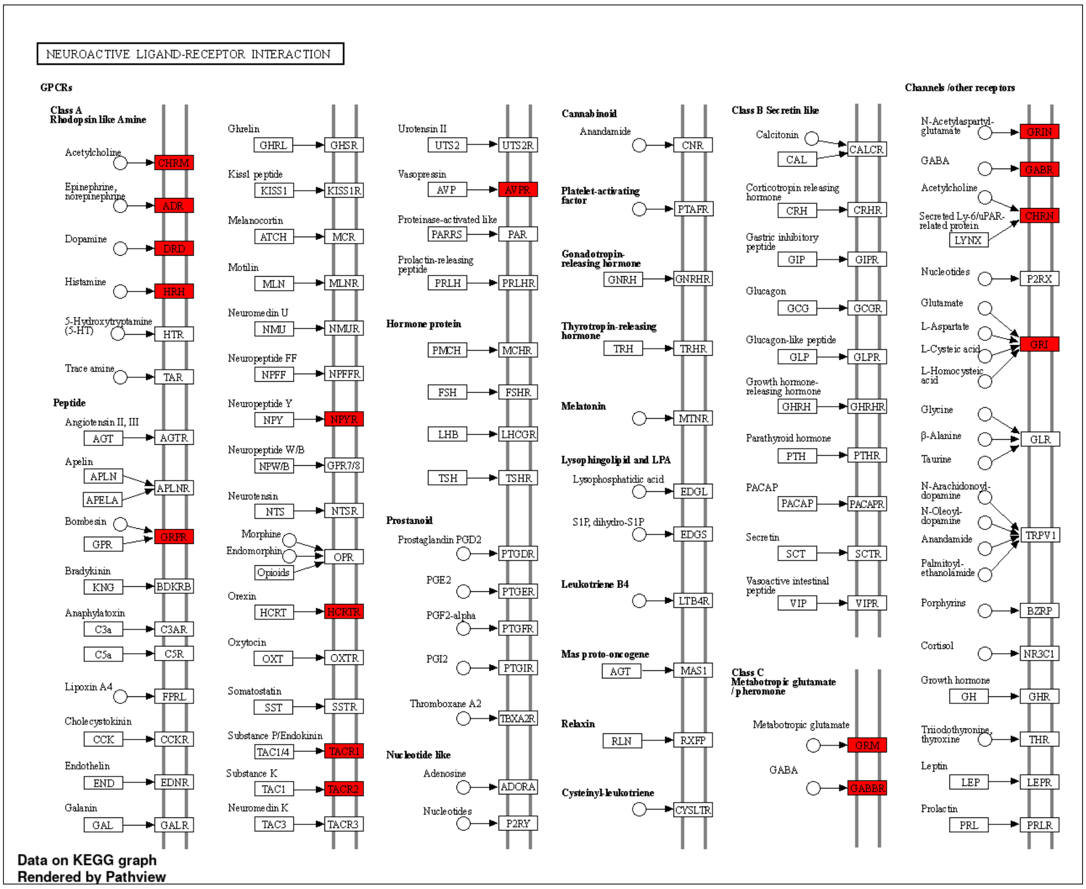

Supplementary material 5 – S5

KEGG pathway – Glutaminergic synapse

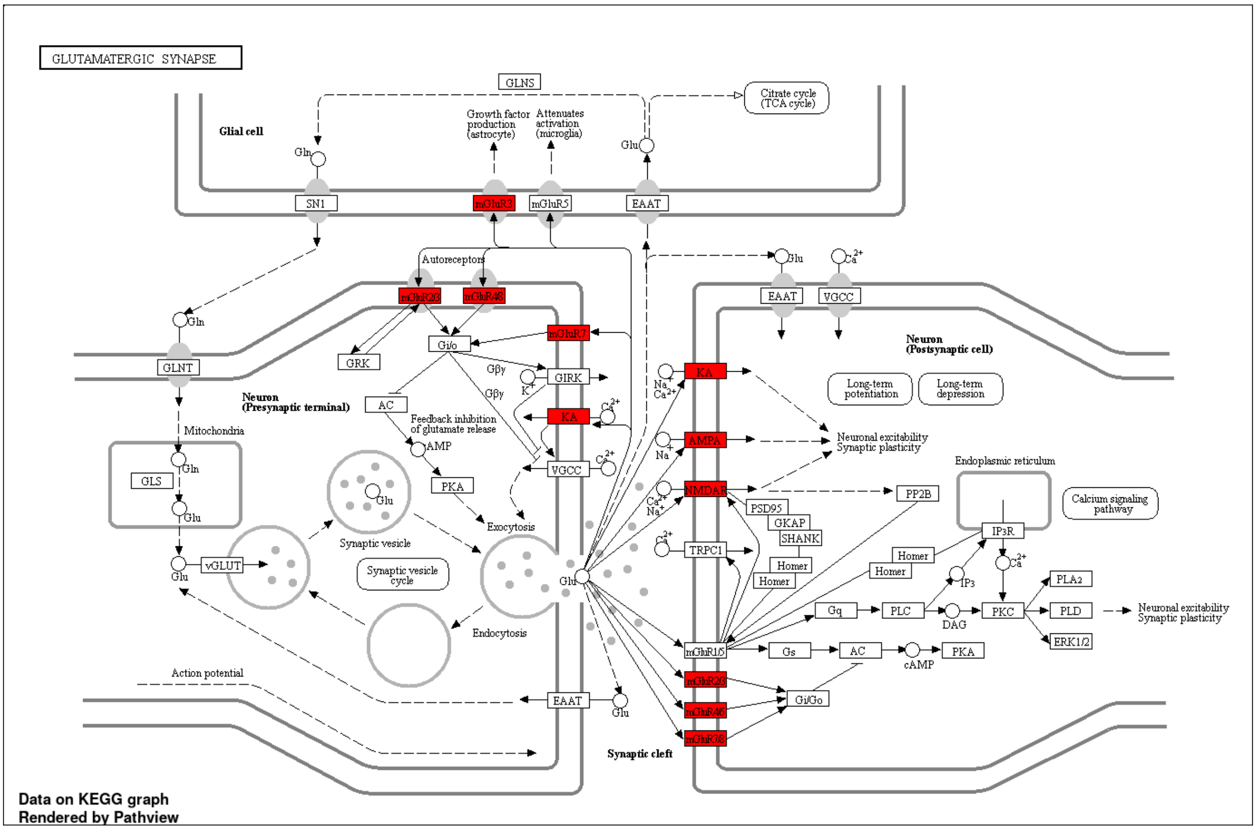

KEGG pathway – GABAergic synapse

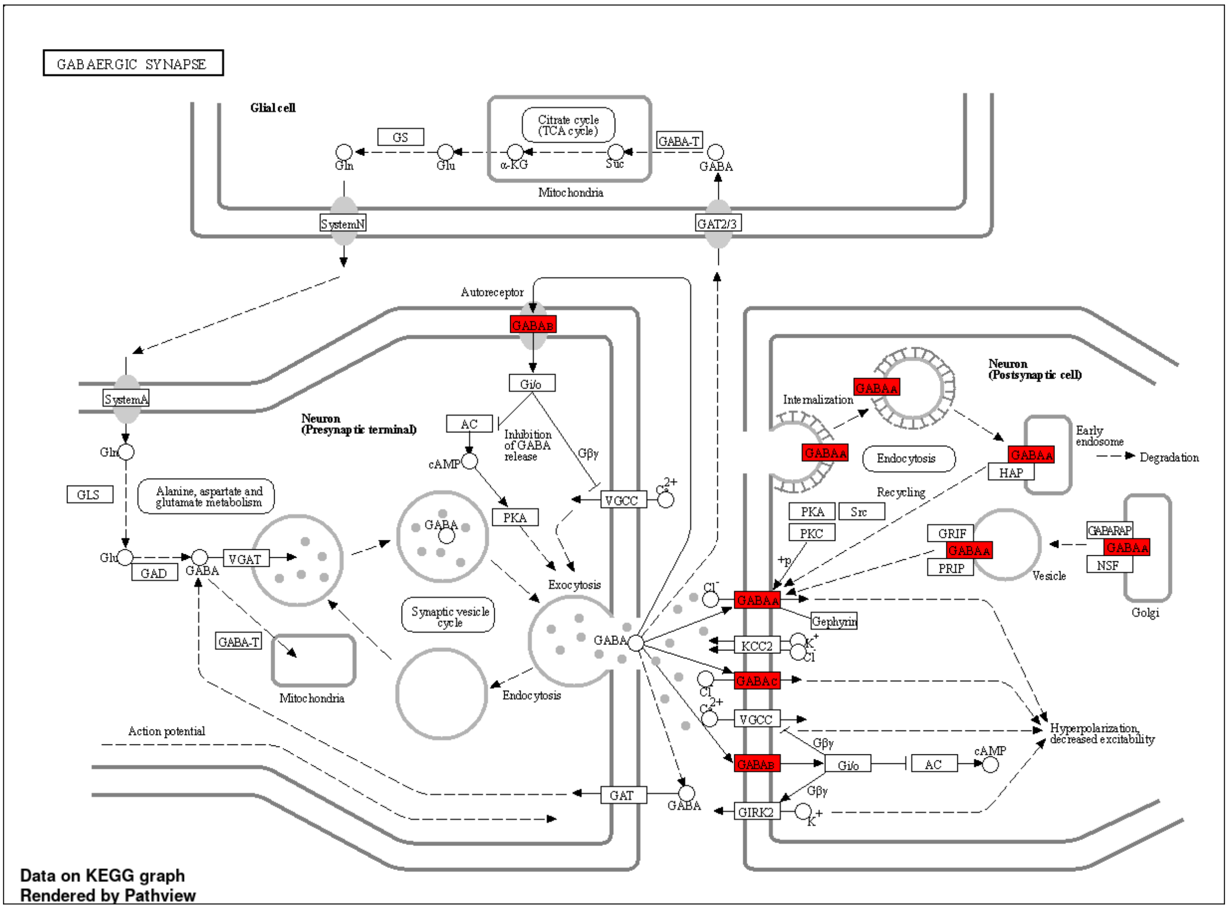

Supplementary material 5 – S5

g:Profiler - g:GOSt

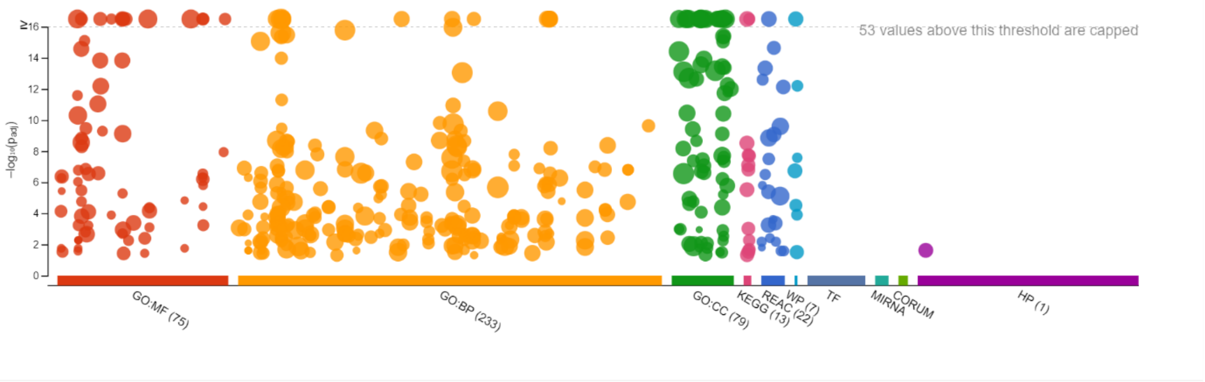

GO:MF — stats

Term name	Term ID	p_{adj}
neurotransmitter receptor activity	GO:0030594	1.273×10^{-36}
postsynaptic neurotransmitter receptor activity	GO:0098960	1.727×10^{-34}
molecular transducer activity	GO:0060089	9.870×10^{-29}
signaling receptor activity	GO:0038023	9.870×10^{-29}
transmembrane signaling receptor activity	GO:0004888	3.057×10^{-28}
neurotransmitter receptor activity involved in regulation of ...	GO:0099529	1.277×10^{-26}
extracellular ligand-gated ion channel activity	GO:0005230	1.288×10^{-26}
transmitter-gated ion channel activity	GO:0022824	1.916×10^{-23}
transmitter-gated channel activity	GO:0022835	1.916×10^{-23}
transmitter-gated ion channel activity involved in regulation...	GO:1904315	3.255×10^{-22}
ligand-gated ion channel activity	GO:0015276	5.144×10^{-22}
ligand-gated channel activity	GO:0022834	7.526×10^{-22}
gated channel activity	GO:0022836	3.961×10^{-17}
GABA receptor activity	GO:0016917	8.409×10^{-17}
glutamate receptor activity	GO:0008066	7.862×10^{-16}
ion channel activity	GO:0005216	2.646×10^{-15}
channel activity	GO:0015267	1.466×10^{-14}
passive transmembrane transporter activity	GO:0022803	1.466×10^{-14}
inorganic molecular entity transmembrane transporter activ...	GO:0015318	6.564×10^{-13}
GABA-A receptor activity	GO:0004890	2.623×10^{-12}
ion transmembrane transporter activity	GO:0015075	9.146×10^{-12}
G protein-coupled receptor activity	GO:0004930	4.982×10^{-11}
G protein-coupled amine receptor activity	GO:0008227	3.442×10^{-10}
acetylcholine receptor activity	GO:0015464	5.152×10^{-10}
transmembrane transporter activity	GO:0022857	7.549×10^{-10}
chloride channel activity	GO:0005254	1.471×10^{-9}
transporter activity	GO:0005215	2.726×10^{-9}
anion channel activity	GO:0005253	4.999×10^{-9}
catecholamine binding	GO:1901338	1.152×10^{-8}
neuropeptide receptor activity	GO:0008188	1.401×10^{-7}
ionotropic glutamate receptor activity	GO:0004970	1.672×10^{-7}
inorganic anion transmembrane transporter activity	GO:0015103	2.630×10^{-7}
ligand-gated anion channel activity	GO:0099095	2.917×10^{-7}
G protein-coupled peptide receptor activity	GO:0008528	2.940×10^{-7}
peptide receptor activity	GO:0001653	4.291×10^{-7}
G protein-coupled glutamate receptor activity	GO:0098988	5.190×10^{-7}
adenylate cyclase inhibiting G protein-coupled glutamate re...	GO:0001640	5.190×10^{-7}
ligand-gated cation channel activity	GO:0099094	6.345×10^{-7}
adrenergic receptor activity	GO:0004935	9.328×10^{-7}
G protein-coupled neurotransmitter receptor activity	GO:0099528	1.552×10^{-6}
excitatory extracellular ligand-gated ion channel activity	GO:0005231	3.338×10^{-6}
group III metabotropic glutamate receptor activity	GO:0001642	3.695×10^{-6}
GABA-gated chloride ion channel activity	GO:0022851	5.260×10^{-6}
norepinephrine binding	GO:0051380	1.476×10^{-5}
inhibitory extracellular ligand-gated ion channel activity	GO:0005237	1.740×10^{-5}
ligand-gated ion channel activity involved in regulation of p...	GO:0099507	3.684×10^{-5}
neurotransmitter binding	GO:0042165	4.348×10^{-5}
peptide binding	GO:0042277	6.974×10^{-5}
amyloid-beta binding	GO:0001540	7.356×10^{-5}
anion transmembrane transporter activity	GO:0008509	7.945×10^{-5}
G protein-coupled acetylcholine receptor activity	GO:0016907	1.286×10^{-4}
cation channel activity	GO:0005261	1.510×10^{-4}
amide binding	GO:0033218	4.142×10^{-4}
serotonin receptor activity	GO:0099589	5.808×10^{-4}
G protein-coupled serotonin receptor activity	GO:0004993	5.808×10^{-4}
benzodiazepine receptor activity	GO:0008503	6.024×10^{-4}
acetylcholine binding	GO:0042166	8.019×10^{-4}
inorganic cation transmembrane transporter activity	GO:0022890	1.124×10^{-3}
adenylate cyclase binding	GO:0008179	1.651×10^{-3}
acetylcholine-gated cation-selective channel activity	GO:0022848	2.029×10^{-3}
cation transmembrane transporter activity	GO:0008324	2.205×10^{-3}
glutamate receptor binding	GO:0035254	4.050×10^{-3}
bombesin receptor activity	GO:0004946	5.355×10^{-3}
G protein-coupled GABA receptor activity	GO:0004965	5.355×10^{-3}
tachykinin receptor activity	GO:0004995	5.355×10^{-3}
AMPA glutamate receptor activity	GO:0004971	5.355×10^{-3}
beta-adrenergic receptor activity	GO:0004939	5.355×10^{-3}
adrenergic receptor binding	GO:0031690	5.529×10^{-3}
peptide YY receptor activity	GO:0001601	1.780×10^{-2}
epinephrine binding	GO:0051379	1.780×10^{-2}
dopamine neurotransmitter receptor activity	GO:0004952	1.780×10^{-2}
alpha-adrenergic receptor activity	GO:0004936	2.666×10^{-2}
G-protein alpha-subunit binding	GO:0001965	2.956×10^{-2}
dopamine binding	GO:0035240	3.728×10^{-2}
PDZ domain binding	GO:0030165	3.775×10^{-2}

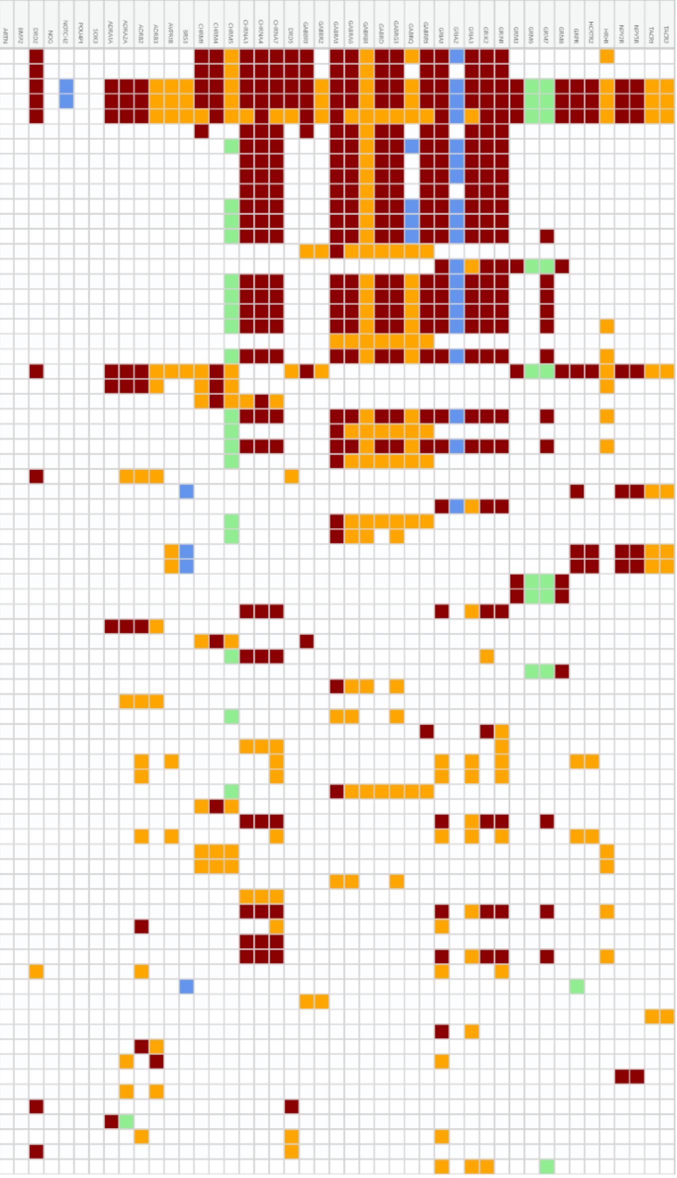

Supplementary material 5 – S5

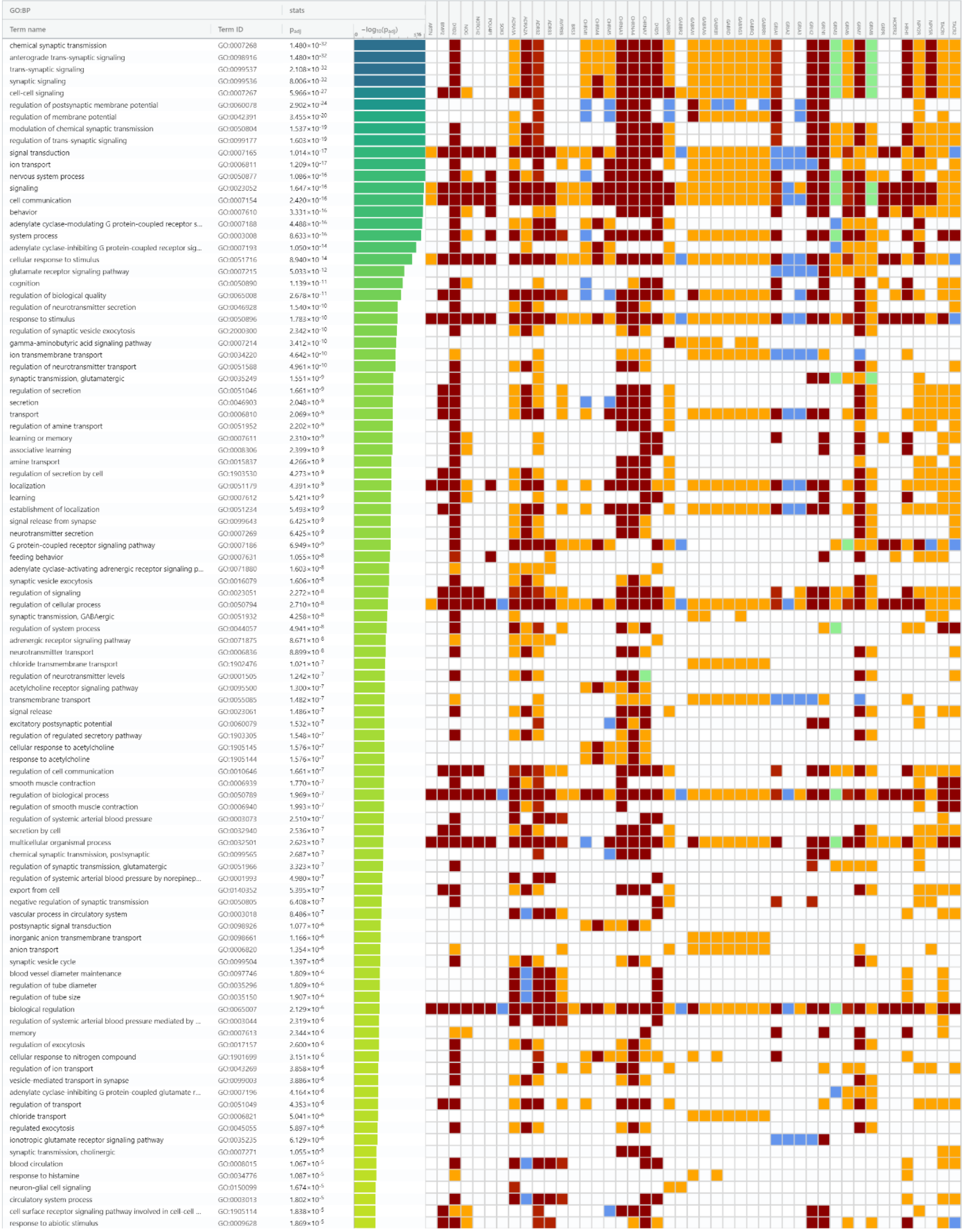

Supplementary material 5 – S5

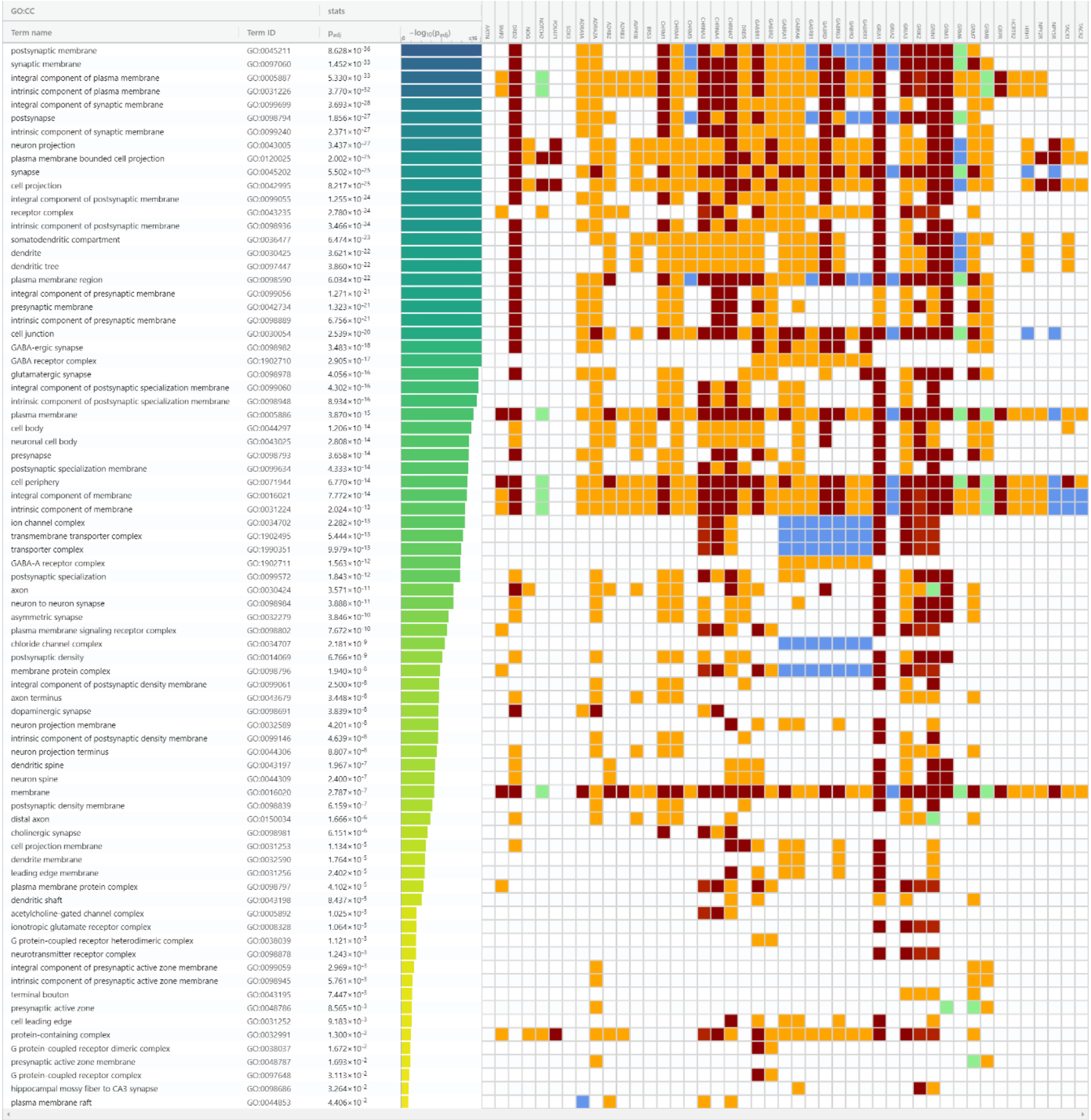

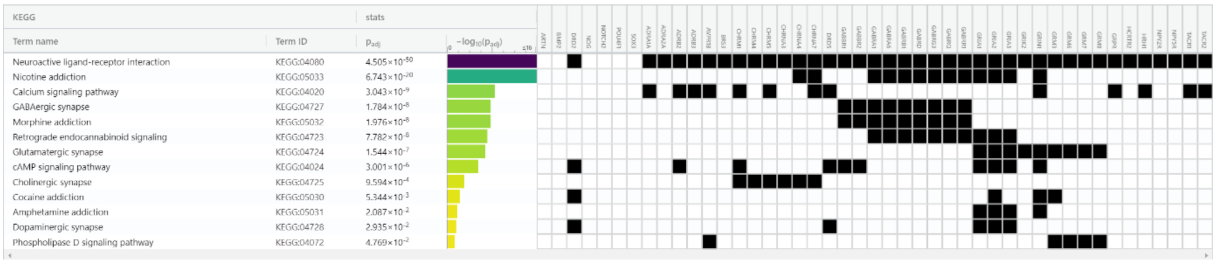

Supplementary material 5 – S5

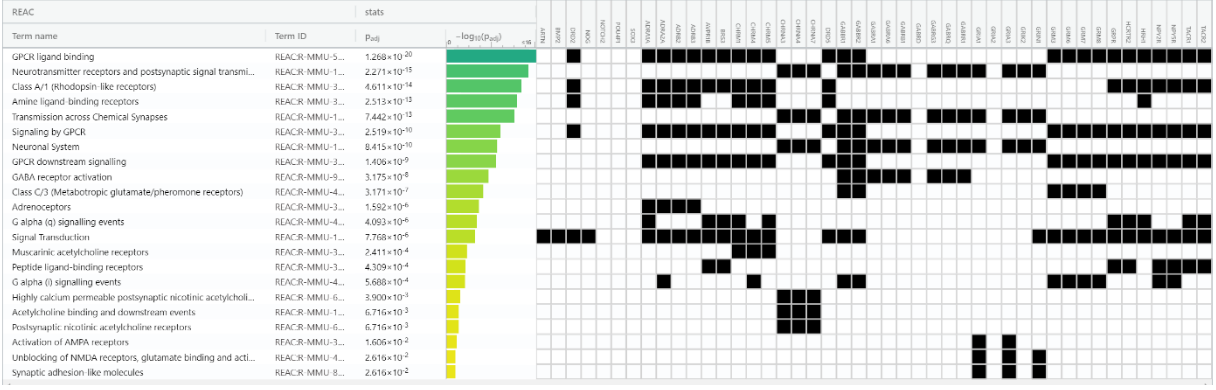

Term name	Term ID	p_{adj}
GPCR ligand binding	REAC:R-MMU-5...	1.268×10^{-20}
Neurotransmitter receptors and postsynaptic signal transmi...	REAC:R-MMU-1...	2.271×10^{-15}
Class A/1 (Rhodopsin-like receptors)	REAC:R-MMU-3...	4.611×10^{-14}
Amine ligand-binding receptors	REAC:R-MMU-3...	2.513×10^{-13}
Transmission across Chemical Synapses	REAC:R-MMU-1...	7.442×10^{-13}
Signaling by GPCR	REAC:R-MMU-3...	2.519×10^{-10}
Neuronal System	REAC:R-MMU-1...	8.415×10^{-10}
GPCR downstream signalling	REAC:R-MMU-3...	1.406×10^{-9}
GABA receptor activation	REAC:R-MMU-9...	3.175×10^{-8}
Class C/3 (Metabotropic glutamate/pheromone receptors)	REAC:R-MMU-4...	3.171×10^{-7}
Adrenoceptors	REAC:R-MMU-3...	1.592×10^{-6}
G alpha (q) signalling events	REAC:R-MMU-4...	4.093×10^{-6}
Signal Transduction	REAC:R-MMU-1...	7.768×10^{-6}
Muscarinic acetylcholine receptors	REAC:R-MMU-3...	2.411×10^{-4}
Peptide ligand-binding receptors	REAC:R-MMU-3...	4.309×10^{-4}
G alpha (i) signalling events	REAC:R-MMU-4...	5.688×10^{-4}
Highly calcium permeable postsynaptic nicotinic acetylcholi...	REAC:R-MMU-6...	3.900×10^{-3}
Acetylcholine binding and downstream events	REAC:R-MMU-1...	6.716×10^{-3}
Postsynaptic nicotinic acetylcholine receptors	REAC:R-MMU-6...	6.716×10^{-3}
Activation of AMPA receptors	REAC:R-MMU-3...	1.606×10^{-2}
Unblocking of NMDA receptors, glutamate binding and acti...	REAC:R-MMU-4...	2.616×10^{-2}
Synaptic adhesion-like molecules	REAC:R-MMU-8...	2.616×10^{-2}

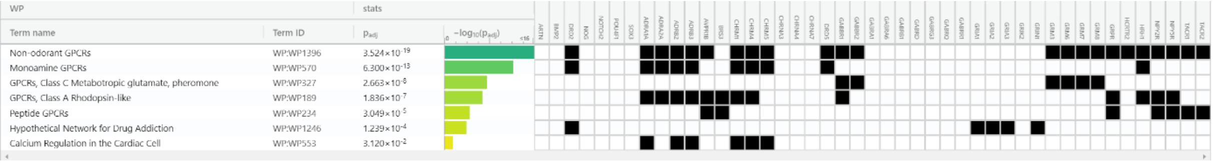

Term name	Term ID	p_{adj}
Non-odorant GPCRs	WP:WP1396	3.524×10^{-19}
Monoamine GPCRs	WP:WP570	6.300×10^{-13}
GPCRs, Class C Metabotropic glutamate, pheromone	WP:WP327	2.663×10^{-8}
GPCRs, Class A Rhodopsin-like	WP:WP189	1.836×10^{-7}
Peptide GPCRs	WP:WP234	3.049×10^{-5}
Hypothetical Network for Drug Addiction	WP:WP1246	1.239×10^{-4}
Calcium Regulation in the Cardiac Cell	WP:WP553	3.120×10^{-2}

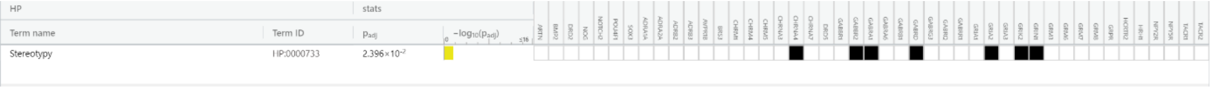

Term name	Term ID	p_{adj}
Stereotypy	HP:0000733	2.396×10^{-2}

Supplementary material 6 – S6

Enrichment analyzes – upregulated genes in the prefrontal cortices of one-month-old females that were prenatally exposed to the triclocarban

Curated reactome

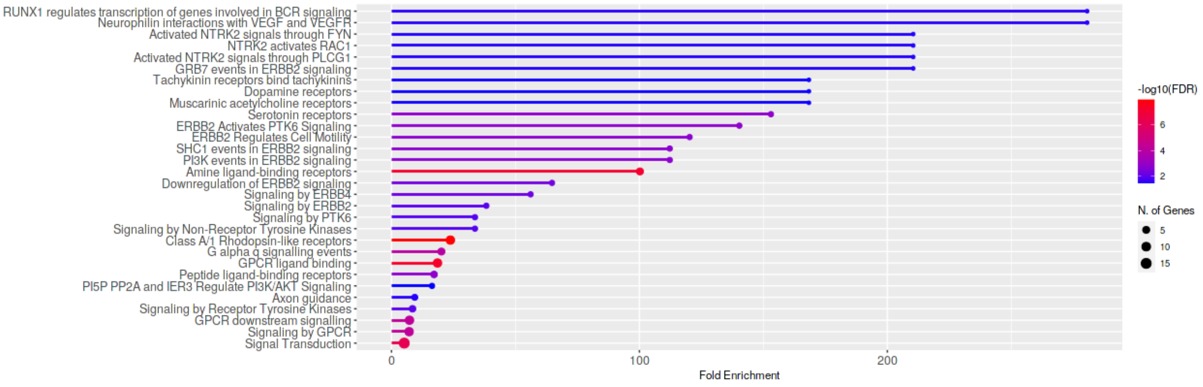

GO Cellular Component

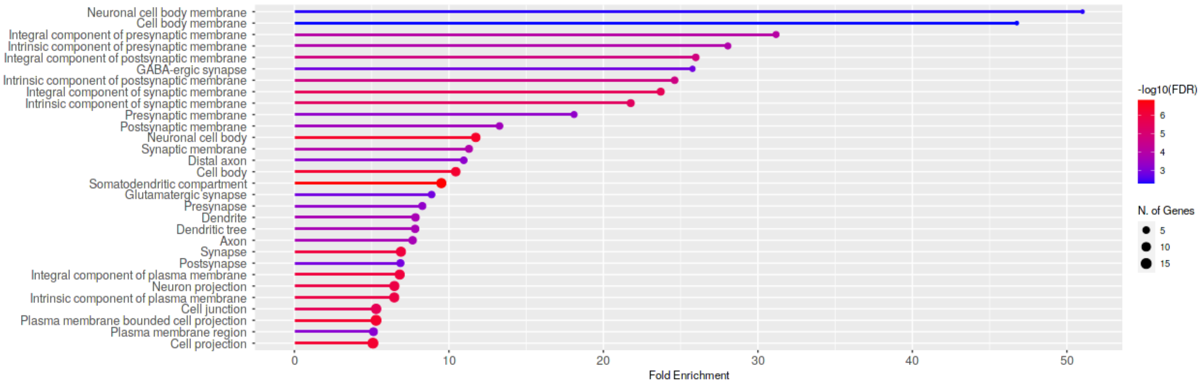

GO Molecular Function

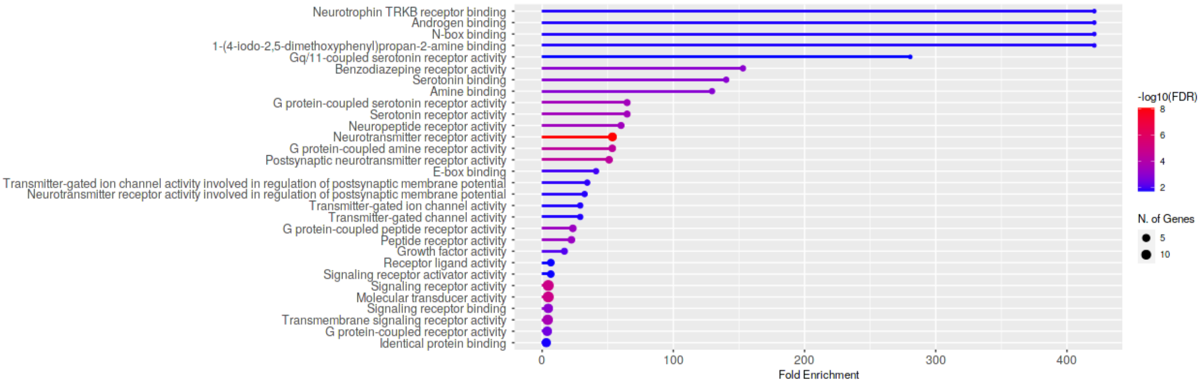

Supplementary material 6 – S6

KEGG

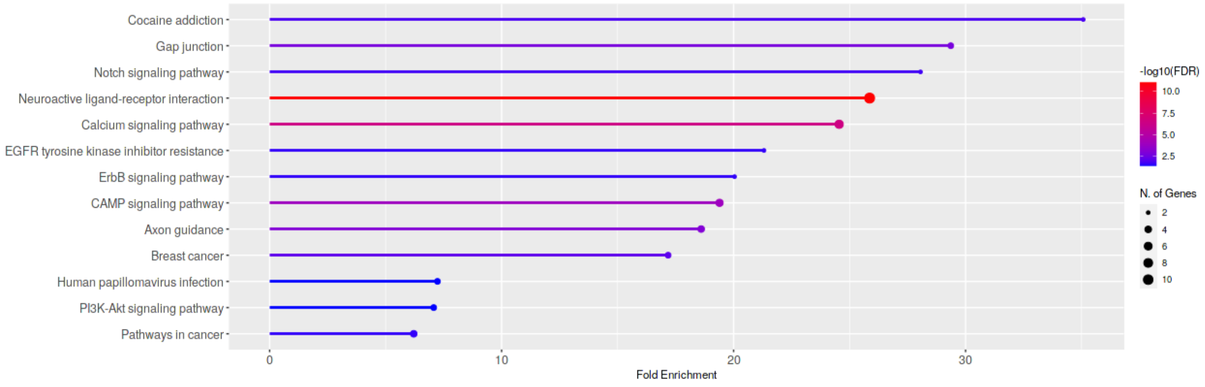

g:Profiler - g:GOSt

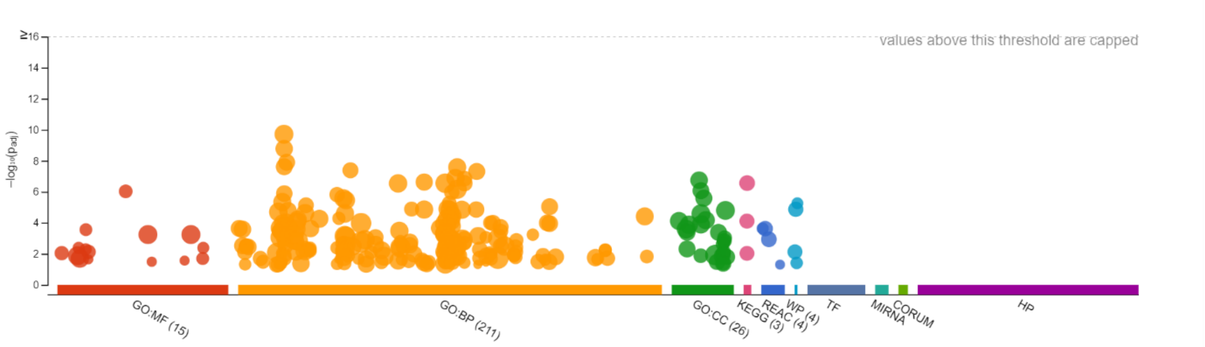

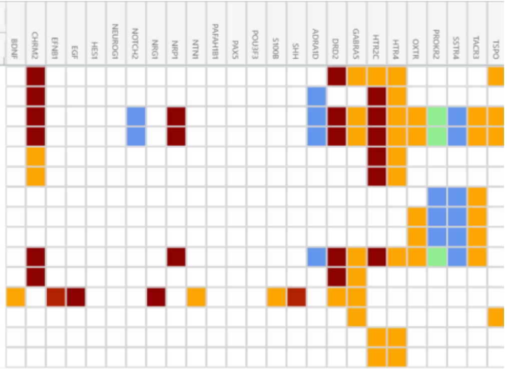

GO:MF		stats
Term name	Term ID	p_{adj}
neurotransmitter receptor activity	GO:0030594	9.421×10^{-7}
G protein-coupled amine receptor activity	GO:0008227	2.791×10^{-4}
signaling receptor activity	GO:0038023	5.751×10^{-4}
molecular transducer activity	GO:0060089	5.751×10^{-4}
serotonin receptor activity	GO:0099589	4.127×10^{-3}
G protein-coupled serotonin receptor activity	GO:0004993	4.127×10^{-3}
neuropeptide receptor activity	GO:0008188	5.171×10^{-3}
G protein-coupled peptide receptor activity	GO:0008528	7.539×10^{-3}
peptide receptor activity	GO:0001653	9.082×10^{-3}
transmembrane signaling receptor activity	GO:0004888	1.345×10^{-2}
postsynaptic neurotransmitter receptor activity	GO:0098960	2.023×10^{-2}
signaling receptor binding	GO:0005102	2.063×10^{-2}
benzodiazepine receptor activity	GO:0008503	2.296×10^{-2}
serotonin binding	GO:0051378	2.753×10^{-2}
amine binding	GO:0043176	3.251×10^{-2}

Supplementary material 6 – S6

GO:BP		stats
Term name	Term ID	p_{adj}
nervous system development	GO:0007399	1.936×10^{-10}
central nervous system development	GO:0007417	1.675×10^{-9}
behavior	GO:0007610	1.260×10^{-8}
brain development	GO:0007420	2.449×10^{-8}
positive regulation of multicellular organismal process	GO:0051240	2.645×10^{-8}
forebrain development	GO:0030900	4.125×10^{-8}
head development	GO:0060322	4.930×10^{-8}
positive regulation of developmental process	GO:0051094	1.281×10^{-7}
positive regulation of nervous system development	GO:0051962	1.409×10^{-7}
positive regulation of cell differentiation	GO:0045597	2.370×10^{-7}
animal organ development	GO:0048513	2.805×10^{-7}
regulation of nervous system development	GO:0051960	2.821×10^{-7}
locomotion	GO:0040011	2.889×10^{-7}
regulation of multicellular organismal process	GO:0051239	6.978×10^{-7}
positive regulation of neurogenesis	GO:0050769	1.085×10^{-6}
sensory organ development	GO:0007423	1.302×10^{-6}
telencephalon development	GO:0021537	1.525×10^{-6}
neurogenesis	GO:0022008	2.663×10^{-6}
neuron differentiation	GO:0030182	3.534×10^{-6}
cell-cell signaling	GO:0007267	4.596×10^{-6}
inner ear development	GO:0048839	5.419×10^{-6}
positive regulation of cell development	GO:0010720	7.330×10^{-6}
synaptic signaling	GO:0099536	9.192×10^{-6}
generation of neurons	GO:0048699	1.279×10^{-5}
ear development	GO:0043583	1.311×10^{-5}
regulation of cell development	GO:0060284	1.400×10^{-5}
regulation of cell differentiation	GO:0045595	1.408×10^{-5}
localization of cell	GO:0051674	1.450×10^{-5}
cell motility	GO:0048870	1.450×10^{-5}
positive regulation of cell population proliferation	GO:0008284	1.675×10^{-5}
movement of cell or subcellular component	GO:0006928	2.023×10^{-5}
positive regulation of cell communication	GO:0010647	2.427×10^{-5}
positive regulation of signaling	GO:0023056	2.549×10^{-5}
regulation of neurogenesis	GO:0050767	3.295×10^{-5}
regulation of developmental process	GO:0050793	3.476×10^{-5}
regulation of multicellular organismal development	GO:2000026	3.879×10^{-5}
nerve development	GO:0021675	5.218×10^{-5}
system development	GO:0048731	5.446×10^{-5}
cell migration	GO:0016477	5.545×10^{-5}
positive regulation of signal transduction	GO:0009967	6.199×10^{-5}
synapse organization	GO:0050808	8.098×10^{-5}
learning or memory	GO:0007611	8.115×10^{-5}
tissue development	GO:0009888	8.943×10^{-5}
neural precursor cell proliferation	GO:0061351	1.005×10^{-4}
anterograde trans-synaptic signaling	GO:0098916	1.016×10^{-4}
chemical synaptic transmission	GO:0007268	1.016×10^{-4}
axon development	GO:0061564	1.030×10^{-4}
developmental process	GO:0032502	1.064×10^{-4}
trans-synaptic signaling	GO:0099537	1.120×10^{-4}
positive regulation of cellular process	GO:0048522	1.165×10^{-4}
synapse assembly	GO:0007416	1.298×10^{-4}
gland development	GO:0048732	1.488×10^{-4}
cognition	GO:0050890	1.669×10^{-4}
ERK1 and ERK2 cascade	GO:0070371	1.844×10^{-4}
signal transduction	GO:0007165	2.160×10^{-4}
cell population proliferation	GO:0008283	2.172×10^{-4}
secretion	GO:0046903	2.180×10^{-4}
MAPK cascade	GO:0000165	2.290×10^{-4}
response to organic cyclic compound	GO:0014070	2.364×10^{-4}
animal organ morphogenesis	GO:0009887	2.494×10^{-4}
cell morphogenesis	GO:0000902	2.654×10^{-4}
regulation of cell population proliferation	GO:0042127	3.321×10^{-4}
smooth muscle contraction	GO:0006939	3.375×10^{-4}
positive regulation of ERK1 and ERK2 cascade	GO:0070374	3.528×10^{-4}
multicellular organism development	GO:0007275	3.602×10^{-4}
negative regulation of biological process	GO:0048519	4.961×10^{-4}
cell morphogenesis involved in neuron differentiation	GO:0048667	5.121×10^{-4}
positive regulation of biological process	GO:0048518	5.374×10^{-4}
cochlea development	GO:0090102	5.845×10^{-4}
feeding behavior	GO:0007631	6.045×10^{-4}
anatomical structure morphogenesis	GO:0009653	6.234×10^{-4}
epithelium development	GO:0060429	6.934×10^{-4}
signaling	GO:0023052	7.354×10^{-4}
cell differentiation	GO:0030154	7.767×10^{-4}
cell junction assembly	GO:0034329	8.474×10^{-4}
tissue morphogenesis	GO:0048729	8.537×10^{-4}
synaptic transmission, GABAergic	GO:0051932	8.621×10^{-4}
cell differentiation involved in kidney development	GO:0061005	8.621×10^{-4}
cell communication	GO:0007154	8.702×10^{-4}
neuron development	GO:0048666	9.242×10^{-4}
cellular developmental process	GO:0048869	1.007×10^{-3}
regulation of biological quality	GO:0065008	1.046×10^{-3}
axonogenesis	GO:0007409	1.050×10^{-3}
regulation of localization	GO:0032879	1.237×10^{-3}
cellular response to stimulus	GO:0051716	1.288×10^{-3}
kidney epithelium development	GO:0072073	1.336×10^{-3}
response to endogenous stimulus	GO:0009719	1.458×10^{-3}
response to organic substance	GO:0010033	1.469×10^{-3}
response to cocaine	GO:0042220	1.595×10^{-3}
regulation of cellular process	GO:0050794	1.664×10^{-3}
regulation of MAPK cascade	GO:0043408	1.725×10^{-3}
multicellular organismal process	GO:0032501	1.768×10^{-3}
telencephalon cell migration	GO:0022029	1.806×10^{-3}
anatomical structure development	GO:0048856	1.825×10^{-3}
negative regulation of neurogenesis	GO:0050768	1.842×10^{-3}
neuroblast proliferation	GO:0007405	1.919×10^{-3}
negative regulation of nervous system development	GO:0051961	2.083×10^{-3}
forebrain cell migration	GO:0021885	2.161×10^{-3}
regulation of secretion	GO:0051046	2.171×10^{-3}
negative regulation of transport	GO:0051051	2.320×10^{-3}

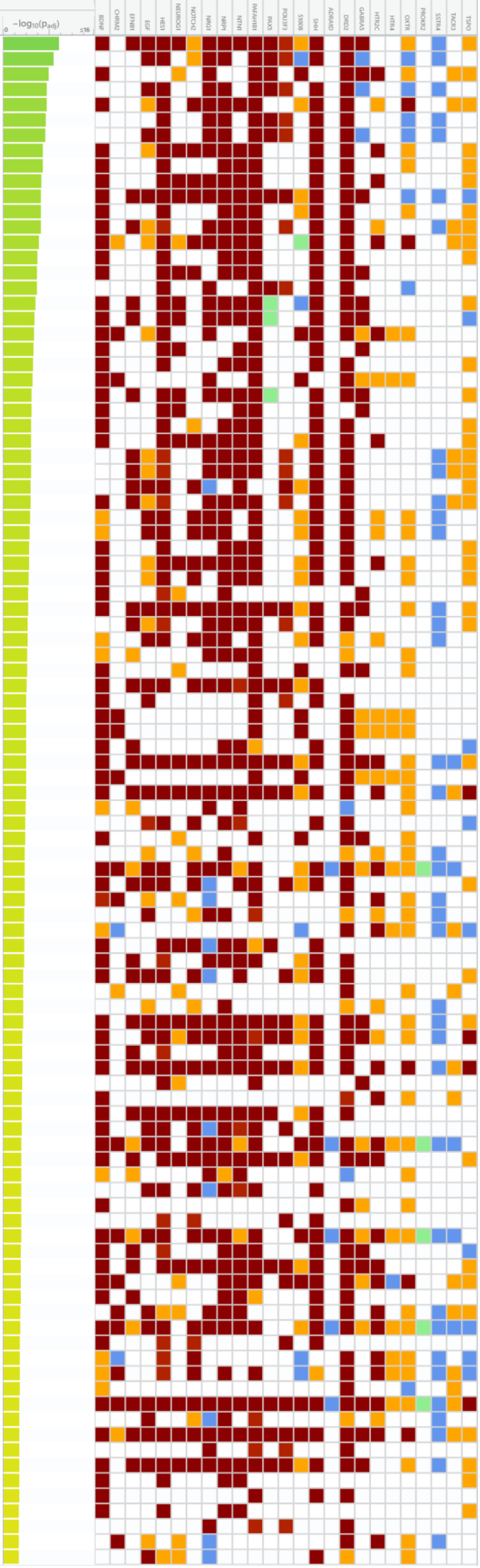

Supplementary material 6 – S6

GO:CC Term name	Term ID	p_{adj}
somatodendritic compartment	GO:0036477	1.797×10^{-7}
neuronal cell body	GO:0043025	8.489×10^{-7}
cell body	GO:0044297	2.596×10^{-6}
plasma membrane bounded cell projection	GO:0120025	1.594×10^{-5}
cell projection	GO:0042995	2.615×10^{-5}
synapse	GO:0045202	6.792×10^{-5}
integral component of plasma membrane	GO:0005887	7.680×10^{-5}
neuron projection	GO:0043005	1.285×10^{-4}
intrinsic component of plasma membrane	GO:0031226	1.309×10^{-4}
cell junction	GO:0030054	2.552×10^{-4}
dendrite	GO:0030425	4.082×10^{-4}
dendritic tree	GO:0097447	4.162×10^{-4}
integral component of synaptic membrane	GO:0099699	9.456×10^{-4}
intrinsic component of synaptic membrane	GO:0099240	1.433×10^{-3}
integral component of presynaptic membrane	GO:0099056	1.580×10^{-3}
intrinsic component of presynaptic membrane	GO:0098889	2.398×10^{-3}
axon	GO:0030424	4.867×10^{-3}
integral component of postsynaptic membrane	GO:0099055	7.804×10^{-3}
intrinsic component of postsynaptic membrane	GO:0098936	9.638×10^{-3}
cell periphery	GO:0071944	1.199×10^{-2}
presynaptic membrane	GO:0042734	1.337×10^{-2}
presynapse	GO:0098793	1.351×10^{-2}
distal axon	GO:0150034	1.605×10^{-2}
synaptic membrane	GO:0097060	3.343×10^{-2}
glutamatergic synapse	GO:0098978	4.301×10^{-2}
GABA-ergic synapse	GO:0098982	4.319×10^{-2}

KEGG Term name	Term ID	p_{adj}
Neuroactive ligand-receptor interaction	KEGG:04080	2.756×10^{-7}
Calcium signaling pathway	KEGG:04020	7.824×10^{-5}
cAMP signaling pathway	KEGG:04024	9.418×10^{-3}

REAC Term name	Term ID	p_{adj}
Amine ligand-binding receptors	REAC:R-MMU-3...	2.323×10^{-4}
Class A/1 (Rhodopsin-like receptors)	REAC:R-MMU-3...	2.379×10^{-4}
GPCR ligand binding	REAC:R-MMU-5...	1.164×10^{-3}
Serotonin receptors	REAC:R-MMU-3...	4.988×10^{-2}

WP Term name	Term ID	p_{adj}
Monoamine GPCRs	WP:WP570	5.491×10^{-6}
Non-odorant GPCRs	WP:WP1396	1.358×10^{-5}
GPCRs, Class A Rhodopsin-like	WP:WP189	7.496×10^{-3}
Id Signaling Pathway	WP:WP512	3.886×10^{-2}

stats: $-\log_{10}(p_{adj})$ 0 … ≤16

Genes: BDNF, CHRM2, EFNB1, EGF, HES1, NEUROG1, NOTCH2, NRG1, NRP1, NTN1, PAFAH1B1, PAX5, POU3F3, S100B, SHH, ADRA1D, DRD2, GABRA5, HTR2C, HTR4, OXTR, PROKR2, SSTR4, TACR3, TSPO

Supplementary material 7 – S7

Specificity description of antibodies used in the western blot. The descriptions are provided by the suppliers and the text is derived from datasheets dedicated to particular antibodies

1. **Rabbit polyclonal anti-BECN1 antibody (sc-11427)**

BECN1 (H-300) is recommended for detection of BECN1 of mouse, rat and human origin by Western Blotting (starting dilution 1:200, dilution range 1:100- 1:1000), immunoprecipitation [1-2 µg per 100-500 µg of total protein (1 ml of cell lysate)], immunofluorescence (starting dilution 1:50, dilution range 1:50-1:500) and immunohistochemistry (including paraffin-embedded sections) (starting dilution 1:50, dilution range 1:50-1:500).

BECN1 (H-300) is also recommended for detection of BECN1 in additional species, including equine, canine, bovine, porcine and avian.

Suitable for use as control antibody for BECN1 siRNA (h): sc-29797, BECN1 siRNA (m): sc-29798, BECN1 shRNA Plasmid (h): sc-29797-SH, BECN1 shRNA Plasmid (m): sc-29798-SH, BECN1 shRNA (h) Lentiviral Particles: sc-29797-V and BECN1 shRNA (m) Lentiviral Particles: sc-29798-V.

Molecular Weight of BECN1: 60 kDa.

Positive Controls: BECN1 (m): 293T Lysate: sc-125053, MCF7 whole cell lysate: sc-2206 or NIH/3T3 whole cell lysate: sc-2210.

2. **Mouse monoclonal anti-β-actin antibody (sc-47778)**

β-Actin (C4) is recommended for detection of β-Actin of mouse, rat, human, avian, bovine, canine, porcine, rabbit, Dictyostelium discoideum and Physarum polycephalum origin by Western Blotting (starting dilution 1:200, dilution range 1:100-1:1000), immunoprecipitation [1-2 µg per 100-500 µg of total protein (1 ml of cell lysate)], immunofluorescence (starting dilution 1:50, dilution range 1:50-1:500), immunohistochemistry (including paraffinembedded sections) (starting dilution 1:50, dilution range 1:50-1:500) and solid phase ELISA (starting dilution 1:30, dilution range 1:30-1:3000); may cross-react with all six known isoforms of Actin in higher vertebrates (including cytoplasmic β- and g-Actin isoforms, skeletal, cardiac, and vascular a-Actin isoforms, and enteric g-Actin isoform).

Suitable for use as control antibody for β-Actin siRNA (h): sc-108069, β-Actin siRNA (m): sc-108070, β-Actin siRNA (r): sc-156106, β-Actin shRNA Plasmid (h): sc-108069-SH, β-Actin shRNA Plasmid (m): sc-108070-SH, β-Actin shRNA Plasmid (r): sc-156106-SH, β-Actin shRNA (h) Lentiviral Particles: sc-108069-V, β-Actin shRNA (m) Lentiviral Particles: sc-108070-V and β-Actin shRNA (r) Lentiviral Particles: sc-156106-V.

β-Actin (C4) X TransCruz antibody is recommended for Gel Supershift and ChIP applications.

Molecular Weight of β-Actin/C-terminal region of β-Actin: 43/15 kDa.

3. **Mouse monoclonal anti-MAP1LC3AB antibody (sc-398822)**

MAP LC3α/b (G-4) is recommended for detection of MAP LC3α and MAP LC3b of mouse, rat and human origin and MAP LC3b2 of human origin by Western Blotting (starting dilution 1:100, dilution range 1:100-1:1000), immunoprecipitation [1-2 µg per 100-500 µg of total protein (1 ml of cell lysate)], immunofluorescence (starting dilution 1:50, dilution range 1:50- 1:500) and solid phase ELISA (starting dilution 1:30, dilution range 1:30- 1:3000).

Suitable for use as control antibody for MAP LC3α/b siRNA (m): sc-156052, MAP LC3α/b shRNA Plasmid (m): sc-156052-SH and MAP LC3α/b shRNA (m) Lentiviral Particles: sc-156052-V.

Molecular Weight of MAP LC3α isoforms: 15/18 kDa.

Molecular Weight of MAP LC3b: 15 kDa.

Positive Controls: HT-1080 whole cell lysate: sc-364183, Saos-2 cell lysate: sc-2235 or U-251-MG whole cell lysate: sc-364176.

4. **Mouse monoclonal anti-nucleoporin p62 antibody (sc-48373)**

Nucleoporin p62 (C-9) is recommended for detection of nucleoporin p62 of mouse, rat and human origin by Western Blotting (starting dilution 1:100, dilution range 1:100-1:1000), immunoprecipitation [1-2 µg per 100-500 µg of total protein (1 ml of cell lysate)], immunofluorescence (starting dilution 1:50, dilution range 1:50-1:500), immunohistochemistry (including paraffinembedded sections) (starting dilution 1:50, dilution range 1:50-1:500) and solid phase ELISA (starting dilution 1:30, dilution range 1:30-1:3000).

Suitable for use as control antibody for nucleoporin p62 siRNA (h): sc-36107, nucleoporin p62 siRNA (m): sc-36108, nucleoporin p62 shRNA Plasmid (h): sc-36107-SH, nucleoporin p62 shRNA Plasmid (m): sc-36108-SH, nucleoporin p62 shRNA (h) Lentiviral Particles: sc-36107-V and nucleoporin p62 shRNA (m) Lentiviral Particles: sc-36108-V.

Molecular Weight of nucleoporin p62: 62 kDa.

5. **Rabbit polyclonal anti-GPER1 antibody (sc-134576)**

GPR30 (H-300) is recommended for detection of GPR30 of mouse, rat and human origin by Western Blotting (starting dilution 1:200, dilution range 1:100-1:1000), immunoprecipitation [1-2 µg per 100-500 µg of total protein (1 ml of cell lysate)], immunofluorescence (starting dilution 1:50, dilution range 1:50-1:500), immunohistochemistry (including paraffin-embedded sections) (starting dilution 1:50, dilution range 1:50-1:500) and solid phase ELISA (starting dilution 1:30, dilution range 1:30-1:3000).

GPR30 (H-300) is also recommended for detection of GPR30 in additional species, including canine and bovine. GPR30 (H-300) is also recommended for detection of GPR30 in additional species, including canine and bovine.

Suitable for use as control antibody for GPR30 siRNA (h): sc-60743, GPR30 siRNA (m): sc-60744, GPR30 siRNA (r): sc-156143, GPR30 shRNA Plasmid (h): sc-60743-SH, GPR30 shRNA Plasmid (m): sc-60744-SH, GPR30 shRNA Plasmid (r): sc-156143-SH, GPR30 shRNA (h) Lentiviral Particles: sc-60743-V, GPR30 shRNA (m) Lentiviral Particles: sc-60744-V and GPR30 shRNA (r) Lentiviral Particles: sc-156143-V.

Molecular Weight of GPR30: 38 kDa.

6. **Mouse monoclonal anti-CYP1A1 antibody (sc-25304)**

CYP1A1 (B-4) is recommended for detection of CYP1A1 of mouse, rat and human origin by Western Blotting (starting dilution 1:100, dilution range 1:100-1:500), immunoprecipitation [1-2 µg per 100-500 µg of total protein (1 ml of cell lysate)], immunofluorescence (starting dilution 1:50, dilution range 1:50-1:500), immunohistochemistry (including paraffin-embedded sections) (starting dilution 1:50, dilution range 1:50-1:500) and solid phase ELISA (starting dilution 1:30, dilution range 1:30-1:3000).

Suitable for use as control antibody for CYP1A1 siRNA (h): sc-41483, CYP1A1 siRNA (m): sc-41484, CYP1A1 siRNA (r): sc-270346, CYP1A1 shRNA Plasmid (h): sc-41483-SH, CYP1A1 shRNA Plasmid (m): sc-41484-SH, CYP1A1 shRNA Plasmid (r): sc-270346-SH, CYP1A1 shRNA (h) Lentiviral Particles: sc-41483-V, CYP1A1 shRNA (m) Lentiviral Particles: sc-41484-V and CYP1A1 shRNA (r) Lentiviral Particles: sc-270346-V.

Molecular Weight of CYP1A1: 56 kDa.

7. Rabbit polyclonal anti-GSK-3β antibody (sc-9166)

GSK-3β (H-76) is recommended for detection of GSK-3β of mouse, rat, human, Xenopus laevis and zebrafish origin by Western Blotting (starting dilution 1:200, dilution range 1:100-1:1000), immunoprecipitation [1-2 µg per 100-500 µg of total protein (1 ml of cell lysate)], immunofluorescence (starting dilution 1:50, dilution range 1:50-1:500), immunohistochemistry (including paraffin-embedded sections) (starting dilution 1:50, dilution range 1:50-1:500) and solid phase ELISA (starting dilution 1:30, dilution range 1:30-1:3000).

GSK-3β (H-76) is also recommended for detection of GSK-3β in additional species, including equine, canine, bovine, porcine and avian.

Suitable for use as control antibody for GSK-3β siRNA (h): sc-35527, GSK-3β siRNA (m): sc-35525, GSK-3β shRNA Plasmid (h): sc-35527-SH, GSK-3β shRNA Plasmid (m): sc-35525-SH, GSK-3β shRNA (h) Lentiviral Particles: sc-35527-V and GSK-3β shRNA (m) Lentiviral Particles: sc-35525-V.

Molecular Weight of GSK-3β: 47 kDa.

Positive Controls: K-562 whole cell lysate: sc-2203, Jurkat whole cell lysate: sc-2204 or HeLa whole cell lysate: sc-2200.

8. Rabbit polyclonal anti-ESR1 antibody (sc-7207)

ERα (H-184) is recommended for detection of estrogen receptor α of mouse, rat and human origin by Western Blotting (starting dilution 1:200, dilution range 1:100-1:1000), immunoprecipitation [1–2 µg per 100–500 µg of total protein (1 ml of cell lysate)], immunofluorescence and immunohistochemistry (including paraffin-embedded sections) (starting dilution 1:50, dilution range 1:50-1:500).

Suitable for use as control antibody for ERα siRNA (h): sc-29305 and ERα siRNA (m): sc-29306.

ERα (H-184) X TransCruz antibody is recommended for Gel Supershift and ChIP applications.

Molecular Weight of ERα: 66 kDa.

Positive Controls: MCF7 whole cell lysate: sc-2206, human breast carcinoma: sc-4260 WB.

9. Goat polyclonal anti-ESR2 antibody (sc-6822)

ERβ (L-20) is recommended for detection of ERβ of human and, to a lesser extent, mouse and rat origin by Western Blotting (starting dilution 1:200, dilution range 1:100-1:1000), immunofluorescence (starting dilution 1:50, dilution range 1:50-1:500) and solid phase ELISA (starting dilution 1:30, dilution range 1:30-1:3000).

ERβ (L-20) is also recommended for detection of ERβ in additional species, including canine.

Suitable for use as control antibody for ERβ siRNA (h): sc-35325, ERβ siRNA (m): sc-35326, ERβ shRNA Plasmid (h): sc-35325-SH, ERβ shRNA Plasmid (m): sc-35326-SH, ERβ shRNA (h) Lentiviral Particles: sc-35325-V and ERβ shRNA (m) Lentiviral Particles: sc-35326-V.

ERβ (L-20) X TransCruz antibody is recommended for Gel Supershift and ChIP applications.

Molecular Weight of ERβ: 56 kDa.

10. Goat polyclonal anti-AHR antibody (sc-8088)

Ah Receptor (N-19) is recommended for detection of Ah Receptor of mouse, rat, human and zebrafish origin by Western Blotting (starting dilution 1:200, dilution range 1:100-1:1000), immunoprecipitation [1-

2 µg per 100-500 µg of total protein (1 ml of cell lysate)], immunofluorescence (starting dilution 1:50, dilution range 1:50-1:500) and solid phase ELISA (starting dilution 1:30, dilution range 1:30-1:3000).

Ah Receptor (N-19) is also recommended for detection of Ah Receptor in additional species, including canine, bovine and porcine.

Suitable for use as control antibody for Ah Receptor siRNA (h): sc-29654, Ah Receptor siRNA (m): sc-29655, Ah Receptor shRNA Plasmid (h): sc-29654-SH, Ah Receptor shRNA Plasmid (m): sc-29655-SH, Ah Receptor shRNA (h) Lentiviral Particles: sc-29654-V and Ah Receptor shRNA (m) Lentiviral Particles: sc-29655-V.

Ah Receptor (N-19) X TransCruz antibody is recommended for Gel Supershift and ChIP applications.

Molecular Weight (predicted) of Ah Receptor: 96 kDa.

Molecular Weight (observed) of Ah Receptor: 122 kDa.

Positive Controls: HeLa whole cell lysate: sc-2200.

11. Rabbit polyclonal anti-ARNT antibody (sc-5580)

Arnt 1 (H-172) is recommended for detection of Arnt 1 of mouse, rat and human origin by Western Blotting (starting dilution 1:200, dilution range 1:100-1:1000), immunoprecipitation [1-2 µg per 100-500 µg of total protein (1 ml of cell lysate)], immunofluorescence (starting dilution 1:50, dilution range 1:50-1:500), immunohistochemistry (including paraffin-embedded sections) (starting dilution 1:50, dilution range 1:50-1:500) and solid phase ELISA (starting dilution 1:30, dilution range 1:30-1:3000).

Arnt 1 (H-172) is also recommended for detection of Arnt 1 in additional species, including equine, canine, bovine and porcine.

Suitable for use as control antibody for Arnt 1 siRNA (h): sc-29733, Arnt 1 siRNA (m): sc-29734, Arnt 1 shRNA Plasmid (h): sc-29733-SH, Arnt 1 shRNA Plasmid (m): sc-29734-SH, Arnt 1 shRNA (h) Lentiviral Particles: sc-29733-V and Arnt 1 shRNA (m) Lentiviral Particles: sc-29734-V.

Arnt 1 (H-172) X TransCruz antibody is recommended for Gel Supershift and ChIP applications.

Molecular Weight of Arnt 1: 95 kDa.

12. Mouse monoclonal anti-BCL-2 antibody (sc-7382)

Bcl-2 (C-2) is recommended for detection of Bcl-2 of mouse, rat and human origin by Western Blotting (starting dilution 1:200, dilution range 1:100-1:1000), immunoprecipitation [1-2 µg per 100-500 µg of total protein (1 ml of cell lysate)], immunofluorescence (starting dilution 1:50, dilution range 1:50-1:500), immunohistochemistry (including paraffin-embedded sections) (starting dilution 1:50, dilution range 1:50-1:500) and flow cytometry (1 µg per 1 x 106 cells).

Suitable for use as control antibody for Bcl-2 siRNA (h): sc-29214, Bcl-2 siRNA (m): sc-29215, Bcl-2 shRNA Plasmid (h): sc-29214-SH, Bcl-2 shRNA Plasmid (m): sc-29215-SH, Bcl-2 shRNA (h) Lentiviral Particles: sc-29214-V and Bcl-2 shRNA (m) Lentiviral Particles: sc-29215-V.

Molecular Weight of Bcl-2: 26 kDa.

13. Mouse monoclonal anti-BAX antibody (sc-7480)

Bax (B-9) is recommended for detection of Baxα and Baxβ of mouse, rat and human origin by Western Blotting (starting dilution 1:200, dilution range 1:100-1:1000), immunoprecipitation [1-2 µg per 100-500 µg of total protein (1 ml of cell lysate)], immunofluorescence (starting dilution 1:50, dilution range 1:50-

1:500), immunohistochemistry (including paraffin-embedded sections) (starting dilution 1:50, dilution range 1:50-1:500) and flow cytometry (1 µg per 1 x 106 cells).

Suitable for use as control antibody for Bax siRNA (h): sc-29212, Bax siRNA (m): sc-29213, Bax shRNA Plasmid (h): sc-29212-SH, Bax shRNA Plasmid (m): sc-29213-SH, Bax shRNA (h) Lentiviral Particles: sc-29212-V and Bax shRNA (m) Lentiviral Particles: sc-29213-V.

Molecular Weight of Bax: 23 kDa.

Positive Controls: Raji whole cell lysate: sc-364236, NAMALWA cell lysate: sc-2234 or Jurkat whole cell lysate: sc-2204.

14. Mouse monoclonal anti-ATG7 antibody (sc-376212)

ATG7 (B-9) is recommended for detection of ATG7 of mouse, rat and human origin by Western Blotting (starting dilution 1:100, dilution range 1:100- 1:1000), immunoprecipitation [1-2 µg per 100-500 µg of total protein (1 ml of cell lysate)], immunofluorescence (starting dilution 1:50, dilution range 1:50-1:500) and solid phase ELISA (starting dilution 1:30, dilution range 1:30- 1:3000).

Suitable for use as control antibody for ATG7 siRNA (h): sc-41447, ATG7 siRNA (m): sc-41448, ATG7 shRNA Plasmid (h): sc-41447-SH, ATG7 shRNA Plasmid (m): sc-41448-SH, ATG7 shRNA (h) Lentiviral Particles: sc-41447-V and ATG7 shRNA (m) Lentiviral Particles: sc-41448-V.

Molecular Weight of ATG7: 71 kDa.

Positive Controls: HeLa whole cell lysate: sc-2200, Jurkat whole cell lysate: sc-2204 or Caki-1 cell lysate: sc-2224.

15. Polyclonal rabbit anti-constitutive androstane receptor antibody (ab-228767)

Product name: Anti-Constitutive androstane receptor antibody

Description: Rabbit polyclonal to Constitutive androstane receptor

Host species: Rabbit Tested applications

Suitable for: WB Species reactivity

Reacts with: Mouse

Predicted to work with: Human, Chimpanzee, Rhesus monkey

Immunogen: Recombinant fragment within Human Constitutive androstane receptor (internal sequence). The exact sequence is proprietary. Database link: Q14994

Positive control WB: Mouse liver lysate.

General notes: The Life Science industry has been in the grips of a reproducibility crisis for a number of years. Abcam is leading the way in addressing this with our range of recombinant monoclonal antibodies and knockout edited cell lines for gold-standard validation. Please check that this product meets your needs before purchasing. If you have any questions, special requirements or concerns, please send us an inquiry and/or contact our Support team ahead of purchase.

Function: binds and transactivates the retinoic acid response elements that control expression of the retinoic acid receptor beta 2 and alcohol dehydrogenase 3 genes. Transactivates both the phenobarbital responsive element module of the human CYP2B6 gene and the CYP3A4 xenobiotic response element.

Tissue specificity: predominantly expressed in liver.

Sequence similarities: belongs to the nuclear hormone receptor family. NR1 subfamily. Contains 1 nuclear receptor DNA-binding domain.

Domain: composed by a short N-terminal domain followed by the DNA binding, hinge, and ligand binding/dimerization domains.

Post-translational modifications: Phosphorylated at Thr-38 by PKC, dephosphorylation of Thr-38 is required for nuclear translocation and activation.

Cellular localization: nucleus. Cytoplasm. Cytoplasm > cytoskeleton. Recruited to the cytoplasm by DNAJC7.
Predicted band size: 40 kDa

16. Rabbit polyclonal anti-aromatase antibody (ab-18995)

Product name: Anti-Aromatase antibody

Description: Rabbit polyclonal to Aromatase

Host species: Rabbit

Tested applications: suitable for: IHC-P, ICC/IF, IHC-Fr, WBmore details

Species reactivity: reacts with: Mouse, Rat, Human

Predicted to work with: Chicken, Cow, Pig

Immunogen: synthetic peptide surrounding amino acid 385 of human Aromatase (Peptide available as ab51924.)

General notes: The Life Science industry has been in the grips of a reproducibility crisis for a number of years. Abcam is leading the way in addressing this with our range of recombinant monoclonal antibodies and knockout edited cell lines for gold-standard validation. Please check that this product meets your needs before purchasing.

Function: catalyzes the formation of aromatic C18 estrogens from C19 androgens.

Tissue specificity: brain, placenta and gonads.

Involvement in disease: defects in CYP19A1 are a cause of aromatase excess syndrome (AEXS) [MIM:139300]; also known as familial gynecomastia. AEXS is characterized by an estrogen excess due to an increased aromatase activity. Defects in CYP19A1 are the cause of aromatase deficiency (AROD) [MIM:107910]. AROD is a rare disease in which fetal androgens are not converted into estrogens due to placental aromatase deficiency. Thus, pregnant women exhibit a hirsutism, which spontaneously resolves after post-partum. At birth, female babies present with pseudohermaphroditism due to virilization of extern genital organs. In adult females, manifestations include delay of puberty, breast hypoplasia and primary amenorrhoea with multicystic ovaries.

Sequence similarities: belongs to the cytochrome P450 family.

Cellular localization: membrane.
Predicted band size: 58 kDa
Observed band size: 58 kDa
Additional bands at: 34 kDa. Supplier is unsure as to the identity of these extra bands.
